# 3-(Pyridine-3-ylmethylene)chroman-4-one and tetralone derivatives: synthesis, *Mycobacterium tuberculosis* CYP121A1 enzyme inhibition and antimycobacterial activity *vs* drug-sensitive and drug-resistant strains

**DOI:** 10.1039/d5md00738k

**Published:** 2026-02-04

**Authors:** Lama A. Alshabani, Jabril M. Abdali, Alistair K. Brown, Damião Pergentino de Sousa, Sam Willcocks, Amit Kumar, Ahmed Y. G. Alhejaili, D. Fernando Estrada, Claire Simons

**Affiliations:** a School of Pharmacy & Pharmaceutical Sciences, Cardiff University Cardiff CF10 3NB UK simonsc@cardiff.ac.uk; b Bioscience Institute, Newcastle University Newcastle upon Tyne NE2 4HH UK; c Department of Pharmaceutical Sciences, Federal University of Paraiba João Pessoa Brazil; d London School of Hygiene & Tropical Medicine London WC1E 7HT UK; e Brunel University London Uxbridge UB83PH UK; f Department of Biochemistry, Jacobs School of Medicine and Biomedical Science, University at Buffalo Buffalo New York-14203 USA

## Abstract

CYP121A1 is a promising cytochrome P450 (CYP) drug target in *Mycobacterium tuberculosis* (*Mtb*) owing to its physiological importance in bacterial cell viability. The continuing rise of multidrug resistant (MDR) and extremely drug resistant (XDR) tuberculosis (TB), offers potential therapeutics with a new mechanism of action to add to the multidrug TB regime. A series of 3-(pyridine-3-ylmethylene)chromanone derivatives (5) with 7-*O*-alkyl/aryl substitutions were explored for CYP121A1 binding and antimycobacterial activity in susceptible and resistant *Mtb* strains. The 3-(pyridine-3-ylmethylene)chroman-4-one derivatives (5) with the 7-*O*-(CH_2_)_3_-phenyl substitution displayed the strongest CYP121A1 binding affinity (*K*_D_ 0.3 to 3.6 μM) compared with the natural substrate (dicyclotyrosine, *K*_D_ 16.8 ± 1.0 μM). Improvements observed in binding affinity from 7-*O*-benzyl to (CH_2_)_2_-phenyl to (CH_2_)_3_-phenyl substitutions are supported by computational studies. Minimum inhibitor concentration (MIC) of the alkyoxyaryl substituted chromanones ranged from 1.5–50 μM (0.5–22.5 μg mL^−1^) against the *H37Rv* wild type strain (*c.f.* isoniazid 1.8 μM (0.2 μg mL^−1^), rifampicin 0.3 μM (0.2 μg mL^−1^), kanamycin 16.1 μM (7.8 μg mL^−1^)) with antimycobacterial activity retained against mono-resistant (isoniazid or rifampicin) and MDR (isoniazid and rifampicin) *Mtb* strains. In contrast, the tetralone derivatives (8) with either the *O*-(CH_2_)_2_-phenyl or *O*-(CH_2_)_3_-phenyl substitutions showed no binding affinity with CYP121A1, possibly owing to binding further away from the haem and failing to displace the 6th axial water ligand, but the *O*-(CH_2_)_3_-phenyl substituted tetralones were the most consistently effective against *H37Rv* strain with MIC of 3 μM (1.1–1.2 μg mL^−1^) and retained activity against the mono-resistant and MDR *Mtb* strains.

## Abstract 2

Chromanone derivatives displayed strong CYP121A1 binding affinity while the tetralone derivatives were very effective antimycobacterial activity against multidrug resistant *Mtb*.

## Graphical abstract

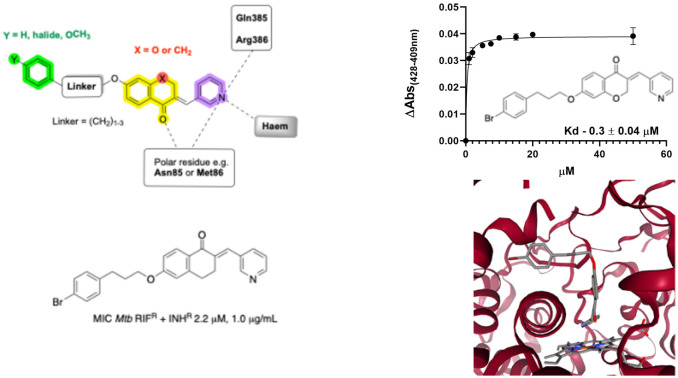

## Introduction

Whilst investigating the *Mtb* genome (*H37Rv*) for the purpose of finding novel drug targets, 20 *Mtb* CYP enzymes were identified.^1^ The density of the *CYP* genes within the *Mtb* genome is approximately 200 times greater than in the human genome, indicative of their requirement for *Mtb* survival and growth.^2,3^ Additionally, the *Mtb* genome encodes a significant number of lipid metabolism genes responsible for lipid synthesis or catabolism^2,3^ and their role in the synthesis and modification of the complex waxy cell wall architectures highlights the significance of lipogenesis in *Mtb*.^1^

CYP121A1 is one of the most promising CYP drug targets in *Mtb*,^2,4^ owing to its physiological importance in bacterial cell viability.^2^ Moreover, CYP121A1 was found to bind tightly with antifungal azoles, including econazole (*K*_D_ 0.027 μM), clotrimazole (*K*_D_ 0.073 μM), and miconazole (*K*_D_ 0.136 μM), the lowest binding affinities observed in comparison with other *Mtb* CYPs.^5^ Furthermore, the order of these azole affinities correlated with their minimum inhibitory concentrations (MIC) potency as antimycobacterial agents (*Mtb* MIC econazole 21.0 μM, 8 μg mL^−1^; clotrimazole 31.9 μM, 11 μg mL^−1^; miconazole 19.2 μM, 8 μg mL^−1^), indicating that CYP121A1 could be an ideal *Mtb* target for the azoles.^6^

The natural substrate of CYP121A1 remained elusive until it was revealed that the transcriptionally linked adjacent gene, *rv2775*, encoded a cyclodipeptide synthase.^2^ This cyclodipeptide synthase is responsible for production of the cyclodipeptide dicyclotyrosine (cYY).^1^ cYY is metabolised by CYP121A1 and converted to mycocyclosin after oxidative intra-molecular C–C cross-linkage between the tyrosyl rings of cYY.^1,2,5,7^ The function of mycocyclosin is undetermined^5^ however, it has been proposed to play an essential role in cell growth or structural stability. Consequently, inhibition of CYP121A1 could result in the loss of an essential function that requires the cYY transformation product, and/or the resulting accumulation of cYY substrate, which may be toxic to *Mtb*, comparable with studies that have shown the effects of cyclic dipeptides on bacterial physiology.^3,8^

**Fig. 1:**
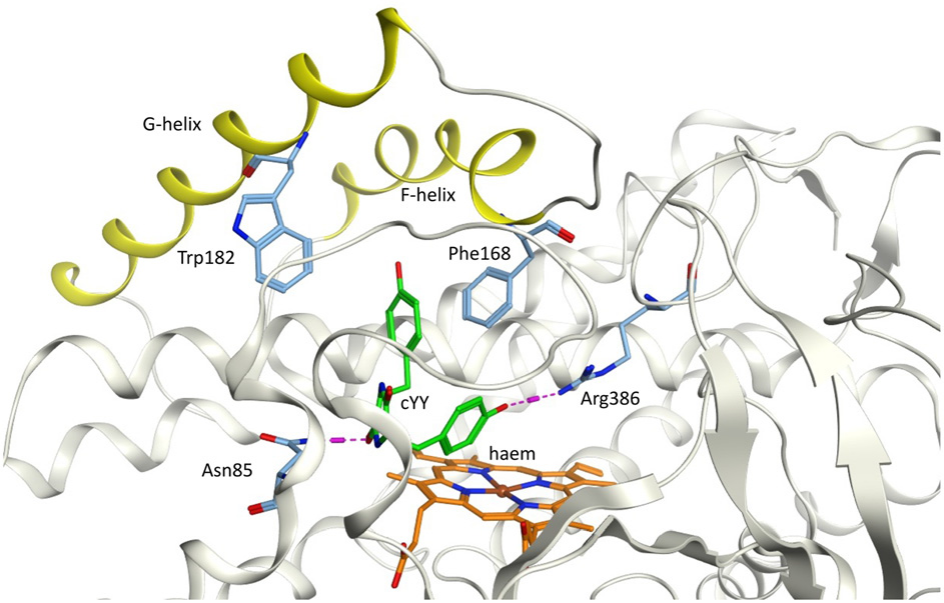
Dicyclotyrosine (cYY) (green) positioned to the side of the haem (orange) in *Mtb* CYP121A1 binding site (pdb: 3G5H) showing the key H-bonding interaction with Arg386 and Asn85. F- and G-helices (yellow) with the aromatic cage amino acids Phe168 and Trp182.

cYY is positioned above and to the side of the haem group and binds within the CYP121A1 active site through multiple direct and water-mediated H-bonding interactions, involving the central diketopiperazine ring and both the phenolic OH groups, one of which binds directly with Arg386 (Fig. 1). On binding of cYY an ‘open’ conformation results with Arg386 displaced initiating CYP121A1 activity.^3^

**Fig. 2:**
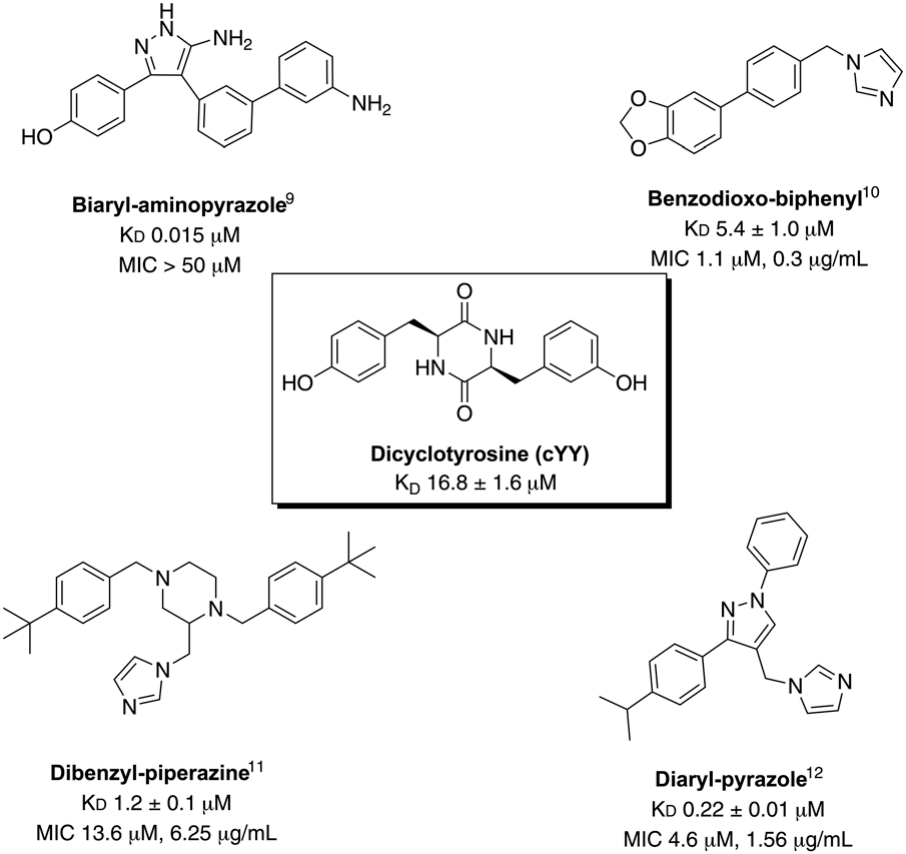
Structures of exemplar *Mtb* CYP121A1 inhibitors and binding affinity (*K*_D_) and antimycobacterial activity (MIC) compared with the natural substrate cYY.

Described *Mtb* CYP121A1 inhibitors all contain a haem binding group that replaces one of the phenol groups in the natural substrate cYY, most commonly the haem binding group is imidazole, which is also present in the antifungal azoles econazole, miconazole and clotrimazole. The haem binding group is then attached to moieties that replace the remaining central diketopiperazine ring and second phenol, for example: biaryl-aminopyrazole,^9^ benzodioxol-biphenyl,^10^ dibenzyl-piperazine^11^ and diaryl-pyrazole^12^ (Fig. 2).

**Fig. 3:**
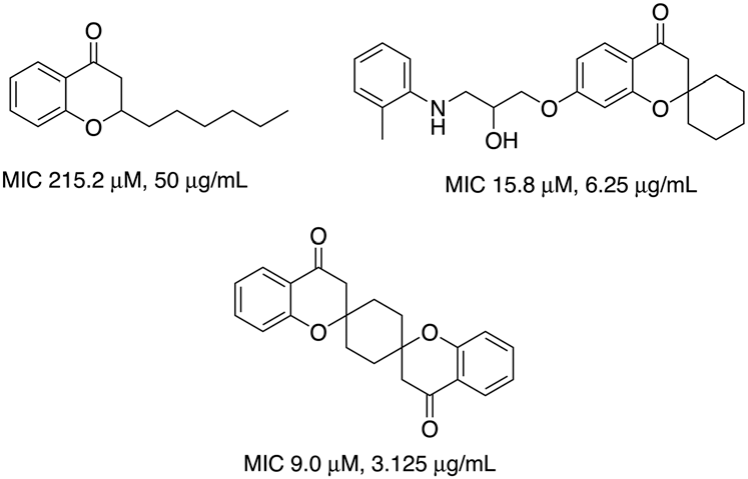
Examples of previously reported chroman-4-ones with chemical structures and antimycobacterial activities (MIC *vs. Mtb H37Rv*).^16,17^

Chroman-4-one represents a promising backbone in drug discovery displaying interesting biological activities including anticancer,^13,14^ antifungal,^15^ antibacterial,^15^ anti-inflammatory,^16^ antidiabetic,^17^ antileishmanial,^18^ insecticidal,^19^ and more crucial to this study, antimycobacterial activity^20,21^ (Fig. 3). Interestingly, a chroman-4-one derivative, 6-hydroxy-2-(3-hydroxyphenyl)chroman-4-one, inhibited a single cytochrome P450 isoform (CYP2C19) at more than 70%.^22^ Whereas other chromanone-like flavonoids, including flavone, flavanone, 5-hydroxyflavone, 5,7-dihydroxyflavone, 2′-, 3′-, and 4′-MeF, and 2′-, 3′-, 4′-, and 6-hydroxylated flavanones have been demonstrated to be substrates of human cytochrome P450s.^23^ However, the biological evaluation of the chroman-4-ones against *Mtb* CYP121A1 has not been explored yet, therefore chroman-4-one compounds were designed to explore inhibitory effects and binding profile in the CYP121A1 enzyme active site and against the organism.

**Fig. 4:**
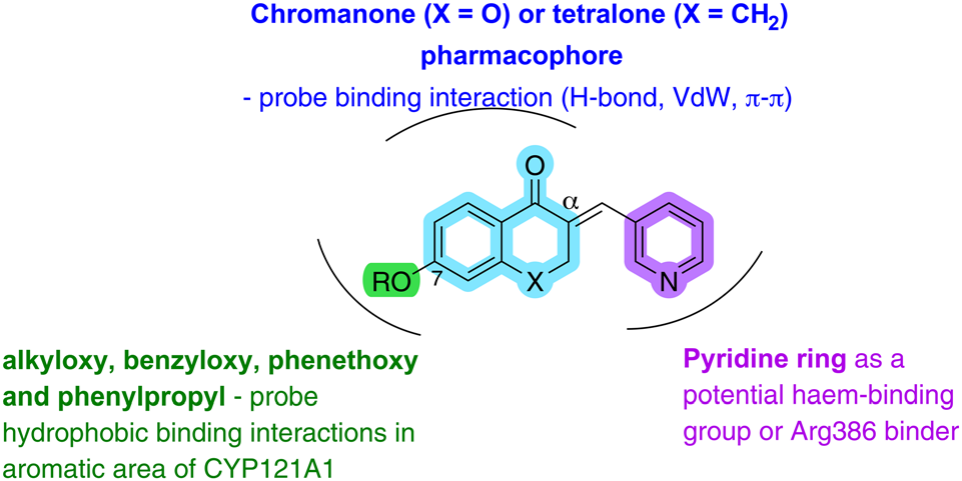
General structure design of chroman-4-one (X = O) and tetralone (X = CH_2_) derivatives.

The general structure design (Fig. 4) includes a chroman-4-one pharmacophore as a mimic of the central diketopiperazine ring of cYY, which may probe additional binding interactions with different amino acids in the active site of CYP121A1. Firstly, H-bonding between the carbonyl O or the O atom at position 1 of the chroman-4-one ring may occur with polar amino acids (*e.g.* Asn85). To explore whether the O in the chroman-4-one is important for binding, tetralone derivatives were also investigated (Fig. 4).

Secondly, a pyridine ring attached to the chroman-4-one or tetralone at the αC through a methylene bridge is proposed to act as a potential haem binding group or to bind with the key Arg386 residue thereby blocking access of the natural substrate cYY, and finally, ethyloxy, benzyloxy, phenethoxy and phenylpropyloxy substituents were added to explore the effect of adding an alkyl/aryl group at C_7_ (Fig. 4) to probe additional hydrophobic binding interactions in the aromatic cage area (*e.g.* Phe168 and Trp182 in the F- and G-helices at the entrance of the access channel – Fig. 1) of the CYP121A1 pocket and to optimise fill of the active site.

## Results and discussion

### Chemistry

The initial study explored C7-derivatives of the chroman-4-one with ethoxy (5b), pentyloxy (5c), unsubstituted and 4-fluorobenzyloxy (5d and 5e) and unsubstituted and 4-fluorophenethoxy (5f and 5g) derivatives prepared and evaluated for CYP121A1 binding affinity and antimycobacterial activity. Based on the biological evaluation of this initial study (Tables 1 and 2), the derivatives were expanded to 4-chloro, 4-bromo and 4-methoxy phenethoxy (5h, 5i and 5j). The equivalent phenylpropoxy derivatives (5k–5o) were also prepared to determine structure activity relationship of the linker length, *i.e.* benzyloxy, phenethoxy and phenylpropoxy and the comparable phenethoxy (8a–8e) and phenylpropyloxy (8f–8j) tetralone derivatives were prepared to determine whether X = O or CH_2_ (Fig. 4) has any beneficial role with respect to biological activity.

**Scheme 1:**
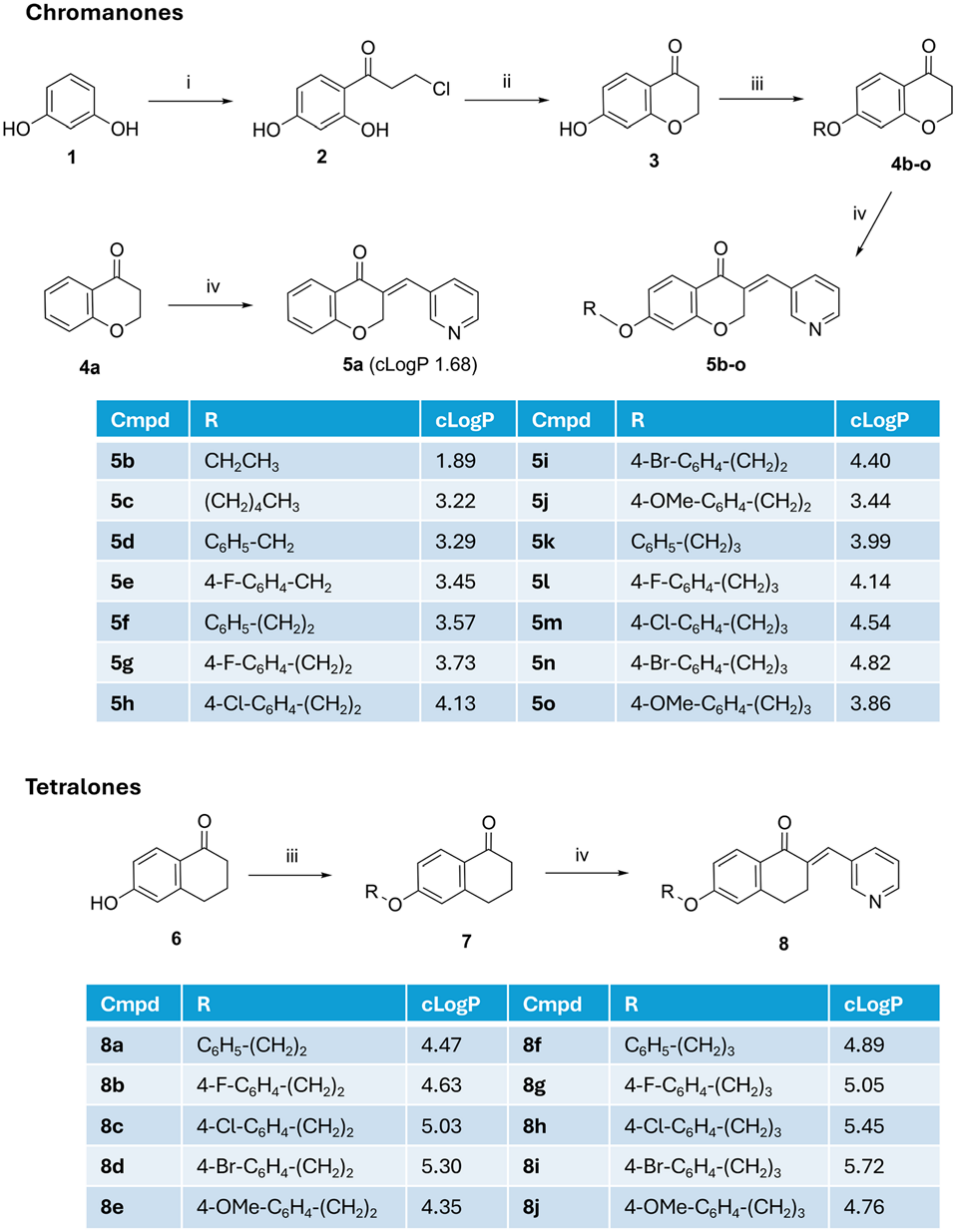
Synthesis of 3-(pyridine-3-ylmethylene)chroman-4-one (5) and tetralone (8) derivatives. *Reagents and conditions*: (i) 3-chloropropionic acid, CF_3_SO_3_H, 85 °C, 1.5 h, 62%; (ii) 2 M aq. NaOH, 0 °C, 3 h, 92%; (iii) alkyl/benzyl/phenethyl/phenylpropyl halide, DMF, K_2_CO_3_, 80 °C, 3 h, 29–95%; (iv) 3-pyridine carboxaldehyde, piperidine, 150 °C, 1–6 h, 26–87%. [clog *P* from ChemDraw – Crippen's fragmentation].

Synthesis of the chroman-4-one final compounds (5) followed a four-step synthetic route (Scheme 1). The Friedel–Crafts product (2) was afforded by reacting resorcinol (1) with 3-chloropropionic acid in trifluoromethane sulfonic acid (CF_3_SO_3_H) for 1.5 h with heating at 85 °C as previously reported.^24^ Reaction of 3-chloro-1-(2,4-dihydroxyphenyl)propan-1-one (2) with 2 M aq. NaOH and stirring for 3 h in an ice-bath resulted in cyclisation to the 7-hydroxychroman-4-one (3).^24^ The simple unsubstituted chromanone (4a) was commercially available while the 7-*O*-substituted chroman-4-one derivatives (4b–o) were prepared by Williamson ether synthesis, by alkylating the 7-hydroxy chroman-4-one (3) with the appropriate alkyl/aryl halide in the presence of K_2_CO_3_ as a base in dry DMF and heating at 80 °C for 3 h (ref. 25) with products obtained in yields of 29–95%. Lower yields were obtained for the phenethoxy derivatives owing to a competing elimination reaction of the phenethyl halides generating styrene byproducts. The 3-(pyridine-3-ylmethylene)chroman-4-one derivatives (5a–o) were prepared by Knoevenagel condensation of chroman-4-one (4a) or 7-alkoxy/aryloxy-chroman-4-one (4b–o) derivatives with 3-pyridine carboxaldehyde in the presence of piperidine as a catalytic base and solvent with heating at 150 °C for 1–6 h.^26^ The tetralone derivatives (8) were prepared *via* a 2-step synthesis with alkylation of 6-hydroxytetralone (6) followed by introduction of the pyridine *via* Knoevenagel condensation of the ethers (7) with 3-pyridine carboxaldehyde as described for the chromanones (Scheme 1). The final pyridine compounds were obtained in yields ranging from 26–87% (Table S1). All final products were confirmed by ^1^H NMR where the olefinic proton peak appeared ∼7.66 ppm, and at 136 ppm in ^13^C NMR.

### CYP121A1 binding affinity (*K*_D_)

CYP121A1 binding affinity was measured using a UV-vis spectral assay.^5^ Binding of the natural substrate cYY leads to a blue shift in the Soret band, indicating type I (indirect) binding resulting in a type I spectral change (417 to 395 nm). In contrast, all the chroman-4-ones, except for the ethyloxy derivative (5b), which showed no spectral change, displayed a red shift of the haem Soret band to a longer wavelength, suggesting type II (direct ligation) binding to the haem iron with displacement of the 6th ligand water molecule^5,27^ (Table 1).

**Table 1:** Soret band shift and *K*_D_ values of pyridine chroman-4-one (5) and tetralone (8) derivatives

Cmpd	Soret shift (nm)	*K*_D_ (μM)	Cmpd	Soret shift (nm)	*K*_D_ (μM)
5a	415 to 416	105 ± 47.3	5n	415 to 420	0.3 ± 0.04
5b	415 to 416	ND	5o	415 to 420	0. 3 ± 0.04
5c	416 to 417	9.2 ± 2.5	8a	415 to 416	ND
5d	416 to 418	5.5 ± 2.0	8b	415 to 416	ND
5e	416 to 418	3.3 ± 1.5	8c	415 to 416	ND
5f	415 to 420	11.0 ± 2.5	8d	No shift	ND
5g	415 to 420	5.9 ± 1.2	8e	No shift	ND
5h	416 to 421	1.1 ± 0.6	8f	415 to 417	ND
5i	415 to 420	1.45 ± 0.3	8g	415 to 416	ND
5j	416 to 422	1.3 ± 0.5	8h	415 to 416	ND
5k	415 to 420	3.6 ± 0.6	8i	415 to 416	ND
5l	415 to 420	1.8 ± 0.02	8j	No shift	ND
5m	415 to 420	0.5 ± 0.01	*cYY*	*417 to 395*	*16.8 ± 1.0*

**Fig. 5:**
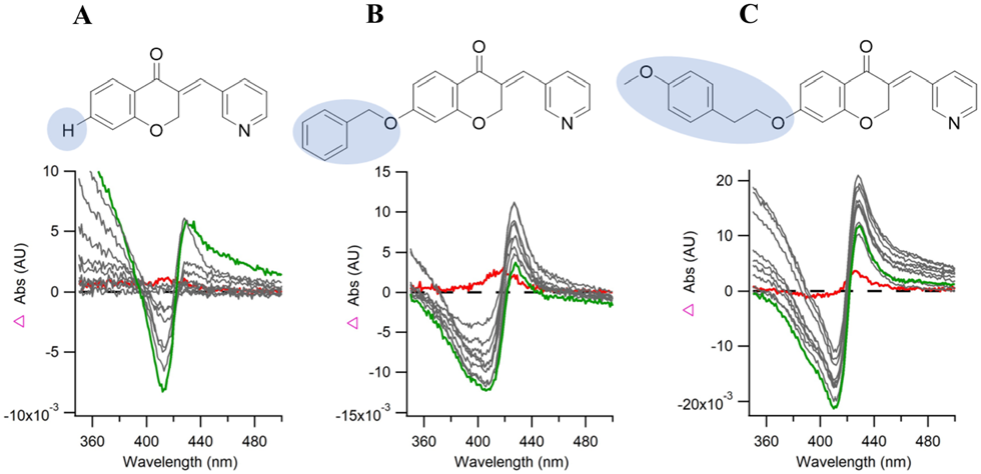
An overlaid difference spectra of compounds (A) 5a, (B) 5d and (C) 5j formed by subtraction of the free-ligand spectrum from each succeeding ligand-bound spectrum collected by titration.

From the initial series (5a–5g) the simple chromanone (5a) and alkoxy derivatives (5b and 5c) showed none or a very small Soret shift (0–1 nm). The unsubstituted and 4-fluorobenzyloxy (5d and 5e) showed a modest 2 nm Soret shift (416 to 418 nm), while the unsubstituted and 4- fluorophenethoxy (5f and 5g) showed 5 nm shift (415–420 nm). This optimal shift was observed with all the phenethoxy and phenylpropoxy derivatives. UV-vis spectra for chroman-4-ones 5a, 5d and 5j are shown in Fig. 5 for illustration of increased Soret shift in the UV-vis assay.

The tightest binding (*K*_D_) was observed with the phenylpropyloxy derivatives 5m (4-Cl phenylpropyloxy, *K*_D_ 0.5 ± 0.01 μM), 5n (4-Br phenylpropyloxy, *K*_D_ 0.3 ± 0.04 μM) and 5o (4-OCH_3_ phenylpropyloxy, *K*_D_ 0.32 ± 0.04 μM) followed by the phenethoxy derivatives 5h (4-Cl phenethoxy, *K*_D_ 1.1 ± 0.6 μM), 5i (4-Br phenethoxy, *K*_D_ 1.45 ± 0.25 μM) and 5j (4-OCH_3_ phenethoxy, *K*_D_ 1.3 ± 0.5 μM) (Table 2). Except for the simple chromanone 5a, all the designed chroman-4-ones (5) exhibited tighter binding compared with the natural substrate (cYY, *K*_D_ 16.8 ± 1.0 μM).

All phenethoxy (8a–8e) and phenylpropyloxy (8f–8j) tetralone derivatives elicited weak or no shift in the Soret band (0–2 nm), comparable with the simple and alkoxy chroman-4-ones (5a–5c) and did not show a sufficient difference signal to quantify binding affinity (Table 1). The difference spectra of the tetralone derivatives exhibited an atypical shape with no peak and only a trough. The UV-vis spectral binding assay specifically measures binding affinity (type I and type II) with the haem, however as CYP121A1 has a large active site cavity (1350 Å^3^)^8^ inhibition of CYP121A1 metabolism of cYY to mycocyclosin by the tetralone derivatives is still the most likely mechanism of action owing to the similarity in structure between the chroman-4-ones (5) and tetralones (8). The tetralones may still block binding of cYY through interaction with an amino acid residue near the haem *e.g.* Ser237, Gly385 or Arg386, without binding directly or indirectly with the haem iron, competitively inhibiting CYP121A1.

### Antimycobacterial activity (MIC) against *Mtb H37Rv* and resistant strains

The 3-(pyridine-3-ylmethylene)chroman-4-one (5) and tetralone (8) derivatives were screened against *Mtb H37Rv* by SPOTi assay.^28^ The MIC_90_ are reported in Table 2 (μM) and Table S2 (μg mL^−1^). As a general trend, all the chromanone compounds (except for the simple chromanone 5a, which had no observable inhibitory activity at the maximum concentration, and the ethoxy derivative 5b) displayed good antimycobacterial activity with optimal activity observed with the benzyloxy (5d, MIC_90_ 1.5 μM, 0.5 μg mL^−1^) and 4-methoxy phenethoxy (5j MIC_90_ 5.2 μM, 2.0 μg mL^−1^) derivatives (Tables 2 and S2). The activity of most of the derivatives (except for 5a and 5b) was comparable with kanamycin (KAN) (MIC_90_ 16.1 μM, 7.8 μg mL^−1^), which was used as a control for *Mtb* growth inhibition. Whereas the more potent derivatives (5d and 5j) were more effective than the standard kanamycin, with activity comparable with the first line agent isoniazid (INH) (MIC_90_ 1.8 μM, 0.24 μg mL^−1^). The least lipophilic derivatives: the ethoxy 5b (clog *P* 2.38, Scheme 1) showed the weakest inhibitory activity (5b, MIC_90_ 266 μM, 75 μg mL^−1^) (Tables 2 and S2), which may be related to reduced drug uptake across the bacterial cell wall, or they may be more susceptible to efflux.

**Table 2:** MIC (μM) of final pyridine chromanones (5) and tetralone (8) derivatives against *Mtb* wild-type and resistant strains

Cmpd	MIC^a^ (μM)					
	*H37Rv*	mc^2^7902	mc^2^8247	mc^2^8245	mc^2^8250	mc^2^8256
5a	ND	ND	ND	ND	ND	ND
5b	266.6	113.8	113.8	113.8	113.8	113.8
5c	25.1	16.9	16.9	16.9	16.9	16.9
5d	1.5	11.7	5.8	11.7	11.7	5.8
5e	11.1	5.5	2.8	5.5	5.5	2.8
5f	12.5	11.2	11.2	11.2	11.2	11.2
5g	25	21.2	10.7	10.7	10.7	10.7
5h	20.8	10.2	5.1	5.1	5.1	5.1
5i	25	9.2	4.6	4.6	4.6	9.2
5j	5.2	20.7	5.2	5.2	5.2	5.2
5k	25	21.5	5.4	10.8	10.8	10.8
5l	25	10.3	5.1	5.1	5.1	5.1
5m	50	9.9	4.9	4.9	4.9	4.9
5n	50	17.8	4.4	8.9	8.9	8.9
5o	25	5.0	2.5	5.0	5.0	5.0
8a	25	22.5	5.6	5.6	2.8	5.6
8b	3	21.4	5.4	5.4	10.7	5.4
8c	25	10.3	2.6	2.6	10.3	2.6
8d	12.5	18.4	2.3	2.3	4.6	2.3
8e	25	20.8	5.2	10.4	2.6	10.4
8f	3	21.7	2.7	2.7	5.4	2.7
8g	3	20.7	2.6	5.2	5.2	5.2
8h	3	19.8	2.5	5.0	5.0	2.5
8i	25	8.9	2.2	2.2	2.2	2.2
8j	3	20.0	10.0	10.0	10.0	10.0
INH	1.8	0.7	0.7	—	—	—
RIF	0.3	0.1	—	0.1	—	—
EMB	—	2.5	4.9	4.9	4.9	2.5
ETH	—	6.0	6.0	6.0	6.0	6.0
KAN	16.1	—	—	—	—	—

a Results are the average of two independent experiments. ND: not detected. INH^R^ – isoniazid resistant, RIF^R^ – rifampicin resistant. *H37Rv* wild type; mc^2^7902 drug susceptible; mc^2^8247 (RIF^R^); mc^2^8245 (INH^R^); mc^2^8250 and mc^2^8256 (RIF^R^ + INH^R^). INH – isoniazid, RIF – rifampicin, EMB – ethambutol, ETH – ethionamide, KAN – kanamycin.

The 3-(pyridine-3-ylmethylene)chroman-4-one derivatives (5) were also screened against *Mtb* mutant strains (susceptible, mono-resistant and MDR mutants (Table S3)),^29^ by REMA assay.^30^ The mc^2^ strains are pantothenate-leucine–arginine (Δ*panCD* Δ*leuCD* Δ*argB*) triple auxotrophic strains of *Mtb H37Rv*, which retain the characteristics of *Mtb* but are devoid of lethality *in vivo* allowing them to be used in a biosafety level 2 (BSL2) laboratory.^29^ Mutations or deletions of *katG* and/or *rpoB* result in the isoniazid (INH) and/or rifampicin (RIF) resistant strains.

An important finding was that all the derivatives (except 5a) retained activity against the resistant strains (Tables 2 and S2), suggesting a lack of cross-resistance with clinically relevant antibiotics. 4-Fluorobenzyl derivative 5e and the phenylpropyloxy derivatives 5l, 5m and 5o showed optimal antimycobacterial activity across all TB strains including *Mtb* resistant strains (5e, R = 4-fluorobenzyl, MIC 2.8–5.5 μM, 1–2 μg mL^−1^; 5l, R = 4-fluorophenylpropyloxy, MIC 5.1–10.2 μM, 2–4 μg mL^−1^; 5m, R = 4-chlorophenylpropyloxy, MIC 4.9–9.9 μM, 2–4 μg mL^−1^; 5o, R = 4-methoxylphenylpropyloxy, MIC 2.5–5.0 μM, 1–2 μg mL^−1^). Moreover, it was obvious that the presence of an aromatic sidechain with a propyloxy extended linker, independent of the nature of the substitution on the aromatic ring, is beneficial for the activity against the INH and RIF resistant strains.

Against the *H37Rv* reference strain, the phenylpropyloxy linked tetralones: the unsubstituted 8f (MIC_90_ 3 μM, 1.1 μg mL^−1^), 4-fluoro 8g (MIC_90_ 3 μM, 1.2 μg mL^−1^), 4-chloro 8h (MIC_90_ 3 μM,1.2 μg mL^−1^) and 4-methoxy 8j (MIC_90_ 3 μM,1.2 μg mL^−1^) derivatives, showed optimal inhibitory activity and all tetralones showed activity against the drug-susceptible triple auxotrophic strain, mc^2^7902 and retained inhibitory activity against the resistant strains (Tables 2 and S2) with the 4-bromo 8i (MIC_90_ 2.2 μM, 1.0 μg mL^−1^) the most effective against all the resistant strains.

### Minimum bacterial concentrations (MBC)

The minimum bactericidal concentrations (MBC_95_) were determined as the lowest concentration of an antimicrobial compound that resulted in 95% killing of the bacterial populations. To define whether an antibacterial agent is bactericidal or bacteriostatic *in vitro*, the MBC/MIC ratio is commonly used.^31^ If the MBC/MIC ratio is ≤4, the effect is considered bactericidal, whereas a ratio >4 denotes a bacteriostatic effect. Control compounds RIF, INH and linezolid (LZD) were used as cidal, cidal, static controls, respectively. As excepted, our data was consistent with previous reports demonstrating that RIF and INH are mycobactericidal and LZD is mycobacteristatic.^32–34^ The MBC_95_ values of the most potent compounds, 5o and 8i, were assessed against *Mtb* mc^2^7902. In both, the regrowth-based assay and the CFU mL^−1^-based MBC determination, the compounds displayed mycobacteristatic properties, with MBC/MIC ratios exceeding 10-fold, indicating a lack of bactericidal activity under the conditions tested (Table 3).

**Table 3:** MBC of 5o and 8i compared with RIF, INH and LZD

	Antibacterial (μM)		
	MIC_95_	MBC_95_ CFU mL^−1^	MBC_95_ regrowth
5o	8.19	237.47	244.21
8i	9.92	87.97	181.58
**RIF**	0.04	0.32	0.22
**INH**	1.84	4.14	2.78
**LZD**	1.18	61.64	10.36

### Cytotoxicity

The cytotoxicity of the most potent compounds, 5o and 8i, were evaluated against uninfected RAW264.7 macrophages, and A549 alveolar type II epithelial cells, following 96-hour exposure; cell viability was measured using standard MTS analysis.^35^ The half-maximal inhibitory concentrations (IC_50_) were calculated to assess potential toxicity, drug efficacy, and corresponding selectivity indices (SI) (Table 4). Compound 5o showed IC_50_ values of 63.74 μM and 42.76 μM against RAW264.7 and A549 cells, respectively, whereas 8i exhibited IC_50_ values of >200 μM and 33.76 μM, respectively. Considering their bacterial MIC_95_ values (5o, 8.19 μM; 8i, 9.92 μM), both compounds displayed favourable SI values in macrophages (7.78 and >20, respectively).^36^ However, the SI for the A549 cells were less encouraging, with values of 5.22 for 5o and 3.4 for 8i, indicating a narrower safety margin. Although 8i demonstrated the most promising macrophage SI, the reduced SI of 8i against A549 cells suggest the MIC lies too close to the cytotoxic threshold.^37^ Therefore, this narrow therapeutic window implies that even minor increases in concentration or exposure duration could potentially result in adverse effects. Nonetheless, the promising macrophage-selective efficacy data is indicative of 8i as a promising lead that merits further structural optimisation to preserve antimicrobial potency while minimising cytotoxicity.

**Table 4:** Antibacterial, cytotoxicity and selectivity indices of 5o and 8i

	MIC_95_ (μM)	Cytotoxicity (IC_50_)		S.I. (IC_50_/MIC_95_)	
	mc^2^7902	A549	RAW264.7	A549	RAW264.7
5o	8.19	42.76	63.74	5.22	7.78
8i	9.92	33.76	>200	3.40	>20.17

### Computational studies

CYP121A1 has been shown to function in its dimeric form with studies showing partial loss of specificity for cYY when the intramolecular contacts on the surface of the dimers are disrupted, resulting in an approximate 75% decrease in catalysis.^38^ However, all crystal structures of CYP121A1 form as monomeric units which may limit the value of these crystal structures for design. However, computational molecular dynamic (MD) simulations allow greater flexibility of the protein and previous computational analysis of CYP121A1 protein–ligand complexes have provided insight into ligand interactions, supporting the use of MD simulations as a predictive tool in computational design and analysis.^9–12,27^

The crystal structure of *Mtb* CYP121A1 complexed with one of our previously described piperazine azole derivatives^39^ (pdb 5O4K) was employed for the initial docking study. This crystal structure was chosen as the piperazine azole derivative displayed a direct (type II) binding interaction with the haem Fe, so it provided a closer representation of the chromanone derivatives based on the UV-vis spectral data (Table 1). The CYP121A1 protein-ligand complexes of exemplar chromanone 4-chloro and 4-methoxy-phenylpropyloxy derivatives (5m and 5o) and the corresponding 4-chloro and 4-methoxy tetralone phenylpropyloxy derivatives (8h and 8j) were generated by molecular docking of derivatives with the crystal structure using molecular operating environment (MOE).^40^ The optimal poses based on binding interactions and energy value were then subjected to 200 ns MD simulations using the Desmond programme of Schrödinger software.^41^

**Fig. 6:**
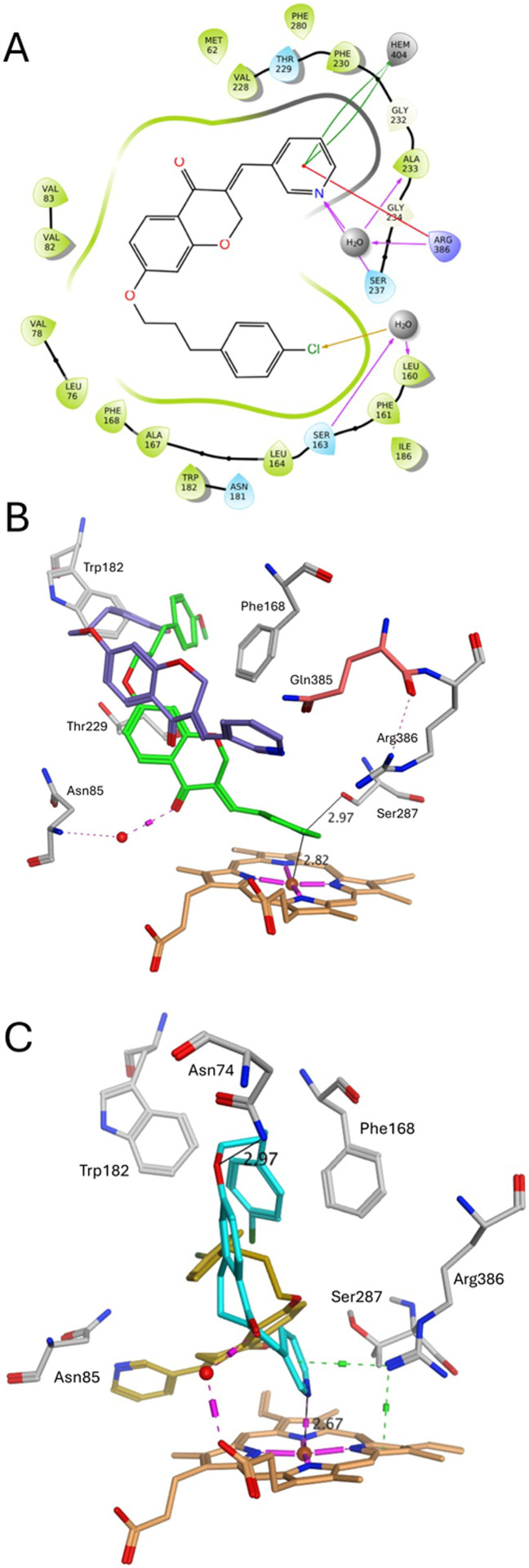
(A) Binding interactions of *Mtb* CYP121A1-chromanone 5m complex after 200 ns MD simulation (B) overlap of *Mtb* CYP121A1-chromanone 5o complex at 90 ns (green, showing binding interactions) and 200 ns (purple) (C) overlap of *Mtb* CYP121A1-tetralone 8h complex at 90 ns (cyan, showing binding interactions) and 200 ns (gold). [Haem, orange; water molecules, red sphere; H-bonds, pink; πι-bonds, green].

The 4-chlorophenylpropyloxy chromanone 5m showed binding interaction between the pyridine N and the haem Fe for 91% of the 200 ns simulation (Fig. S1), with a N–Fe distance of 2.80 Å in the final frame of the simulation. Additional binding interactions were observed for the pyridine of 5m with Arg386 and Ser237, and a water mediated halide binding interaction between the chlorobenzene and Leu160 (Fig. 6A). The 4-methoxyphenylpropyloxy chromanone 5o showed binding interaction between the pyridine N and either the haem of Fe (2.82 Å) or Ser237-OH for 43% and 33% of the 200 ns simulation, respectively (Fig. S1). Overlap of the *Mtb* CYP121A1-chromanone 5o complex after 90 and 200 ns MD simulation show pyridine N–Fe haem binding at 90 ns, while at 200 ns the pyridine moved away from direct binding with the haem but is positioned close to Gln385 to form a binding interaction (direct or H_2_O-mediated) with pyridine N to block access to the haem catalytic site (Fig. 6B).

For both tetralone derivatives (8h and 8j) a similar shift from pyridine N–Fe haem binding was observed around 90 ns of the simulation time (Fig. S1, S2 and 6C), however for both compounds the pyridine flips away from the haem, resulting in a binding interaction between the pyridine N and Asn85.This computational prediction may explain the reduced shift in the Soret band (Table 1), and subsequent unusual difference spectra observed, for the tetralone derivatives.

## Conclusions

Biological evaluation of the chromanone series (5a–o) indicated that addition of an aryl group to the linker improved binding affinity and extension of the linker from CH_2_ to (CH_2_)_2–3_ was also beneficial, possibly owing to the ability to form different rotational conformations to optimise fit within the active site cavity and perhaps more importantly extend towards the opening of the access channel and formation of π–π interactions with the aromatic cage residues Phe168 and Trp182 (Fig. 1 and 6). The benzyloxy (5d–5e), phenethoxy (5f–j) and phenylpropyloxy (5k–o) chromanone derivatives generally showed antimycobacterial activity (MIC) comparable with kanamycin (16.1 μM) and notably retained antibacterial activity against drug resistant strains, including MDR (rifampicin and isoniazid resistant) strains.

In comparison, the phenylpropyloxy chromanone derivatives (5k–o) *versus* the phenylpropyloxy tetralone derivatives (8f–8j) did show a difference, with improved MIC observed for the tetralone derivatives (Table 2) and the phenylpropyloxy derivatives (8f–8j), with MIC comparable with ethambutol (MIC 2.5 μM), generally outperformed the phenethoxy tetralone derivatives (8a–e) against the *Mtb* wild-type *H37Rv* strain. In contrast no CYP121A1 binding was observed experimentally (Table 1) for the tetralone derivatives (8), although computational studies predicted the tetralones would bind with CYP121A1 in a comparable manner to the chromanones (Fig. 6), suggesting the binding assay may not be sensitive enough for the tetralone pyridines or they may experimentally complex with the CYP121A1 protein in a manner that results in the unusual difference spectra, which does not allow for a calculation of *K*_D_. The tetralones all retained antibacterial activity against mono-drug resistant and MDR drug resistant *Mtb* strains.

We have previously shown a correlation between antimycobacterial activity and lipophilicity, with improved MIC observed for more lipophilic derivatives, presumably owing to increased uptake across the lipophilic mycobacterial outer membrane.^12,17^ This would apply to the finding here of improved MIC for the more lipophilic tetralone derivatives compared with the chromanones derivatives (clog *P* values in Scheme 1). However, a balance is needed between lipophilicity and drug-like properties. All chromanone derivatives (5) and the unsubstituted, fluoro and methoxy tetralone derivatives (8a–b, 8e–f and 8j) fulfil Lipinski's Ro5, while the chloro and bromo tetralone derivatives (8c–d, 8h–i) and the 4-fluoro-phenylpropoxy derivative (8g) had one violation (clog *P* > 5 Scheme 1). Further studies will focus on combining the (CH_2_)_2–3_ linkers with a range of ‘chromanone-like’ pharmacophores while adhering to drug-like properties as well as exploring alternative haem/Arg386 binding moieties (*e.g.* imidazole and triazole) to derive further SAR to optimise structure design for CYP121A1 binding and antimycobacterial activity with a focus on improving antimicrobial potency, in particular against MDR *Mtb* strains, while minimising toxicity.

## Experimental

### General

All chemicals, reagents and solvents were purchased from Sigma-Aldrich, Alfa Aesar, VWR, Acros and Fluka and dried prior to use over molecular sieves (4 Å). For column chromatography silica gel (Fluka Kieselgel 60) was used and TLC was performed on pre-coated silica plates (dimension 20 × 20 cm) (ALUGRAM^®^ SIL G/UV_254_) with visualisation *via* UV light (254 nm). Melting points were determined on an electrothermal instrument (Gallenkamp) and are uncorrected. ^1^H, ^13^C (DEPT), and ^19^F NMR spectra were recorded on a Bruker Advance DP500 spectrometer operating at 500, 125 and 470 MHz, respectively using either CDCl_3_ or DMSO-*d*_6_. Chemical shifts are given in parts per million (ppm) relative to the internal standard tetramethylsilane (Me_4_Si); coupling constants (*J* values) were given in Hertz (Hz). All NMR characterisations were made by comparison with previous NMR spectra of the appropriate structure class and/or predictions from ChemDraw. HPLC-HRMS spectra were performed at the University of Bath, UK on a Zorbax Eclipse Plus C18 Rapid Resolution 2.1 × 50 mm, 1.8 μm particle size using a 7.5-minute gradient method 5 : 95 v/v water: methanol with 0.1% formic acid as additive. Microanalysis was performed by MEDAC Ltd (Chobham, UK). Experimental methods for all intermediate compounds (2, 3, 4 and 7), antimycobacterial minimum inhibition concentration (MIC), CYP121A1 binding affinity (*K*_D_) and computational methods can be found in the SI. Computational clog *P* obtained from ChemDraw (Crippen's fragmentation).

### Chemistry

#### General method for the preparation of pyridine products 5 and 8

A mixture of alkylated chroman-4-one (5) or alkylated tetralone (7) (1 equiv.), 3-pyridine-carboxaldehyde (1.5 equiv.) and piperidine (2 equiv.) was stirred at 150 °C for 1 h. Then, the reaction mixture was diluted with EtOAc (40 mL mmol^−1^) and washed with H_2_O (3 × 20 mL) and brine (3 × 20 mL), dried (MgSO_4_) and evaporated under reduced pressure. The crude product was purified by gradient column chromatography.

#### (*E*)-3-(Pyridin-3-ylmethylene)chroman-4-one (5a)

Prepared from chroman-4-one (4a) (0.14 mL, 1.5 mmol). The product was eluted with EtOAc to give the product (5a) as a beige solid. Yield: 0.16 g (65%), m.p: 152–154 °C (lit. m.p.^24^ 120–122 °C), TLC (EtOAc) *R*_f_ 0.32. ^1^H NMR (CDCl_3_): *δ* 8.59 (s, 1H, Ar), 8.49 (d, *J* = 4.0 Hz, 1H, Ar), 8.23 (d, *J* = 8.0 Hz, 1H, Ar), 7.77 (s, 1H, C=CH), 7.72 (d, *J* = 8.0 Hz, 1H, Ar), 7.67 (m, 1H, Ar), 7.46 (d, *J* = 8.0 Hz, 1H, Ar), 7.41 (m, 1H, Ar), 7.25 (dd, *J* = 4.5, 7.5 Hz, 1H, Ar), 3.83 (s, 2H, CH_2_). Anal. calcd: C 75.94%, H 4.67%, N 5.90%. Found: C 75.94%, H 4.58%, N 5.90%.

#### (*E*)-7-Ethoxy-3-(pyridin-3-ylmethylene)chroman-4-one (5b)

Prepared from 7-ethoxychroman-4-one (4b) (0.19 g, 1.0 mmol). The product was eluted with EtOAc to give the product (5b) as a brown solid. Yield: 0.15 g (39%), m.p: 118–120 °C, TLC (EtOAc) *R*_f_ 0.32. ^1^H NMR (CDCl_3_): *δ* 8.60 (s, 1H, Ar), 8.50 (d, *J* = 3.5 Hz, 1H, Ar), 8.12 (d, *J* = 9.0 Hz, 1H, Ar), 7.80 (d, *J* = 8.0 Hz, 1H, Ar), 7.70 (s, 1H, C=CH), 7.31 (dd, *J* = 5.0, 7.5 Hz, 1H, Ar), 6.97 (dd, *J* = 2.0, 9.0 Hz, 1H, Ar), 6.81 (d, *J* = 2.5 Hz, 1H, Ar), 4.13 (q, *J* = 7.0 Hz, 2H, CH_2_), 3.82 (s, 2H, CH_2_), 1.48 (t, *J* = 7.0 Hz, 3H, CH_3_). ^13^C NMR (CDCl_3_): *δ* 176.61 (C=O), 163.45 (C, Ar), 158.28 (C, Ar), 152.49 (CH, Ar), 150.17 (CH, Ar), 147.93 (CH, Ar), 136.55 (C=CH), 134.58 (C, Ar), 127.24 (CH, Ar), 123.44 (CH, Ar), 123.41 (C, Ar), 117.63 (C, Ar), 114.95 (CH, Ar), 100.56 (CH, Ar), 64.24 (CH_2_), 29.07 (CH_2_), 14.54 (CH_3_). Anal. calcd: C 72.12%, H 5.34%, N 4.95%. Found: C 72.08%, H 5.40%, N 4.86%.

#### (*E*)-7-(Pentyloxy)-3-(pyridin-3-ylmethylene)chroman-4-one (5c)

Prepared from 7-pentyloxychroman-4-one (4c) (0.23 g, 1.0 mmol). The product was eluted with EtOAc to give the product (5c) as a beige solid. Yield: 0.20 g (63%), m.p: 94–96 °C, TLC (EtOAc) *R*_f_ 0.40. ^1^H NMR (CDCl_3_): *δ* 8.62 (s, 1H, Ar), 8.51 (d, *J* = 3.5 Hz, 1H, Ar), 8.12 (d, *J* = 7.0 Hz, 1H, Ar), 7.83 (d, *J* = 6.5 Hz, 1H, Ar), 7.72 (s, 1H, C=CH), 7.33 (dd, *J* = 4.5, 6.5 Hz, 1H, Ar), 6.98 (dd, *J* = 2.0, 7.5 Hz, 1H, Ar), 6.82 (d, *J* = 2.0 Hz, 1H, Ar), 4.05 (t, *J* = 5.5 Hz, 2H, CH_2_), 3.83 (s, 2H, CH_2_), 1.85 (quintet, *J* = 5.5 Hz, 2H, CH_2_), 1.48 (m, 2H, CH_2_), 1.42 (*m*, 2H, CH_2_), 0.96 (t, *J* = 6.0 Hz, 3H, CH_3_). ^13^C NMR (CDCl_3_): *δ* 175.96 (C=O), 163.58 (C, Ar), 158.33 (C, Ar), 154.38 (CH, Ar), 150.27 (CH, Ar), 147.79 (CH, Ar), 136.44 (C=CH), 135.65 (C, Ar), 126.87 (CH, Ar), 123.81 (CH, Ar), 122.87 (C, Ar), 117.50 (C, Ar), 115.44 (CH, Ar), 101.49 (CH, Ar), 68.91 (CH_2_), 28.64 (CH_2_), 28.53 (CH_2_), 28.06 (CH_2_), 22.30 (CH_2_), 14.35 (CH_3_). Anal. calcd: C 74.28%, H 6.54%, N 4.33%. Found: C 74.23%, H 6.60%, N 4.14%.

#### (*E*)-7-(Benzyloxy)-3-(pyridin-3-ylmethylene)chroman-4-one (5d)

Prepared from 7-benzyloxychroman-4-one (4d) (0.24 g, 1.0 mmol). The product was eluted with EtOAc to give the product (5d) as a beige solid. Yield: 0.16 g (48%), m.p: 152–154 °C, TLC (EtOAc) *R*_f_ 0.32. ^1^H NMR (CDCl_3_): *δ* 8.60 (s, 1H, Ar), 8.50 (d, *J* = 3.5 Hz, 1H, Ar), 8.15 (d, *J* = 7.5 Hz, 1H, Ar), 7.79 (d, *J* = 6.5 Hz, 1H, Ar), 7.71 (s, 1H, C=CH), 7.45 (m, 5H, Ar), 7.31 (dd, *J* = 4.0, 6.5 Hz, 1H, Ar), 7.07 (dd, *J* = 2.0, 7.0 Hz, 1H, Ar), 6.91 (d, *J* = 2.0 Hz, 1H, Ar), 5.17 (s, 2H, CH_2_), 3.82 (s, 2H, CH_2_). ^13^C NMR (CDCl_3_): *δ* 175.98 (C=O), 163.03 (C, Ar), 158.21 (C, Ar), 154.46 (CH, Ar), 150.22 (CH, Ar), 147.80 (CH, Ar), 136.55 (C, Ar), 136.45 (C=CH), 135.63 (C, Ar), 129.02 (2 × CH, Ar), 128.64 (CH, Ar), 128.42 (2 × CH, Ar), 126.97 (CH, Ar), 123.82 (CH, Ar), 122.90 (C, Ar), 117.78 (C, Ar), 115.69 (CH, Ar), 102.02 (CH, Ar), 70.66 (CH_2_), 28.38 (CH_2_). Anal. calcd: C 76.95%, H 4.99%, N 4.08%. Found: C 77.04%, H 4.92%, N 3.88%.

#### (*E*)-7-((4-Fluorobenzyl)oxy)-3-(pyridin-3-ylmethylene)chroman-4-one (5e)

Prepared from 7-((4-fluorobenzyl)oxy)chroman-4-one (4e) (0.10 g, 0.4 mmol). The product was eluted with EtOAc to give the product (5e) as a white solid. Yield: 0.08 g (54%), m.p: 152–154 °C, TLC (EtOAc) *R*_f_ 0.42. ^1^H NMR (CDCl_3_): *δ* 8.58 (s, 1H, Ar), 8.48 (s, 1H, Ar), 8.13 (d, *J* = 9.0 Hz, 1H, Ar), 7.68 (d, *J* = 8.0 Hz, 1H, Ar), 7.66 (s, 1H, C=CH), 7.42 (dd, *J* = 5.0, 9.0 Hz, 2H, Ar), 7.23 (dd, *J* = 4.5, 7.5 Hz, 1H, Ar), 7.10 (t, *J* = 8.5 Hz, 2H, Ar), 7.03 (dd, *J* = 2.5, 9.0 Hz, 1H, Ar), 6.87 (d, *J* = 2.5 Hz, 1H, Ar), 5.10 (s, 2H, CH_2_), 3.79 (s, 2H, CH_2_). ^19^F NMR (CDCl_3_): *δ* −113.29 (Ar–F). ^13^C NMR (CDCl_3_): *δ* 176.54 (C=O), 163.72 and 161.75 (d, ^1^*J*_CF_ = 246.8 Hz, C–F, Ar), 162.90 (C, Ar), 158.15 (C, Ar), 152.56 (CH, Ar), 150.11 (CH, Ar), 147.91 (CH, Ar), 136.61 (C=CH), 134.55 (C, Ar), 131.43 and 131.41 (d, ^4^*J*_CF_ = 2.5 Hz, C, Ar), 129.49 and 129.43 (d, ^3^*J*_CF_ = 7.5 Hz, 2 × CH, Ar), 127.47 (CH, Ar), 123.52 (C, Ar), 123.48 (CH, Ar), 118.05 (C, Ar), 115.84 and 115.67 (d, ^2^*J*_CF_ = 21.4 Hz, 2 × CH, Ar), 115.05 (CH, Ar), 101.25 (CH, Ar), 69.87 (CH_2_), 29.08 (CH_2_). Anal. calcd: C 73.12%, H 4.46%, N 3.87%. Found: C 72.85%, H 4.43%, N 3.78%.

#### (*E*)-7-Phenethoxy-3-(pyridin-3-ylmethylene)chroman-4-one (5f)

Prepared from 7-phenethoxychroman-4-one (4f) (0.21 g, 0.8 mmol). The product was eluted with EtOAc to give the product (5i) as a light beige solid. Yield: 0.12 g (40%), m.p: 130–132 °C, TLC (petroleum ether – EtOAc 1 : 1 v/v) *R*_f_ 0.15. ^1^H NMR (CDCl_3_): *δ* 8.85 (s, 1H, Ar), 8.49 (d, *J* = 4.4 Hz, 1H, Ar), 8.12 (d, *J* = 9.0 Hz, 1H, Ar), 7.68 (d, *J* = 7.9 Hz, 1H, Ar), 7.65 (s, 1H, C=CH), 7.35 (t, *J* = 7.3 Hz, 2H, Ar), 7.28 (m, 5H, Ar), 6.98 (d, *J* = 8.9 Hz, 1H, Ar), 6.81 (s, 1H, Ar), 4.26 (t, *J* = 7.0 Hz, 2H, CH_2_), 3.80 (s, 2H, CH_2_), 3.16 (t, *J* = 7.0 Hz, 2H, CH_2_). ^13^C NMR (CDCl_3_): *δ* 176.55 (C=O), 163.24 (C, Ar), 158.2 (C, Ar), 152.5 (CH, Ar), 150.13 (CH, Ar), 147.9 (CH, Ar), 137.56 (CH, Ar), 136.55 (C=CH), 134.54 (C, Ar), 128.96 (2 × CH, Ar), 128.61 (2 × CH, Ar), 127.29 (CH, Ar), 126.75 (CH, Ar), 123.43 (C, Ar), 123.42 (CH, Ar), 117.77 (C, Ar), 114.92 (CH, Ar), 100.72 (CH, Ar), 69.29 (OCH_2_), 35.48 (CH_2_), 29.06 (CH_2_). Anal. calcd: C 77.29%, H 5.36%, N 3.92%. Found: C 76.78%, H 5.51%, N 3.84%.

#### (*E*)-7-(4-Fluorophenethoxy)-3-(pyridin-3-ylmethylene)chroman-4-one (5g)

Prepared from 7-(4-fluorophenethoxy)chroman-4-one (4g) (0.23 g, 0.8 mmol). The product was eluted with EtOAc to give the product (5g) as a white solid. Yield: 0.15 g (50%), m.p: 140–142 °C, TLC (petroleum ether – EtOAc 1 : 1 v/v) *R*_f_ 0.18. ^1^H NMR (CDCl_3_): *δ* 8.57 (s, 1H, Ar), 8.49 (d, *J* = 4.5 Hz, 1H, Ar), 8.12 (d, *J* = 9.0 Hz, 1H, Ar), 7.68 (d, *J* = 7.8 Hz, 1H, Ar), 7.65 (s, 1H, C=CH), 7.25 (m, 4H, Ar), 7.03 (t, *J* = 8.6 Hz, 2H, Ar), 6.96 (dd, *J* = 2.0, 8.9 Hz, 1H, Ar), 6.80 (d, *J* = 1.9 Hz, 1H, H-8), 4.23 (t, *J* = 6.8 Hz, 2H, CH_2_), 3.8 (s, 2H, CH_2_), 3.12 (t, *J* = 6.8 Hz, 2H, CH_2_). ^19^F NMR: *δ* −116.23 (Ar–F). ^13^C NMR (CDCl_3_): *δ* 176.53 (C=O), 163.14 (C, Ar), 161.8 (d, ^1^*J*_CF_ = 244.7 Hz, C-F, Ar), 158.19 (C, Ar), 152.50 (CH, Ar), 150.15 (CH, Ar), 147.93 (CH, Ar), 136.54 (C=CH), 134.50 (CH, Ar), 133.30 (d, ^4^*J*_CF_ = 3.6 Hz, C, Ar), 130.40 (d, ^3^*J*_CF_ = 7.9 Hz, 2 × CH, Ar), 127.34 (CH, Ar), 123.47 (CH, Ar), 123.42 (CH, Ar) 117.83 (C, Ar), 115.41 (d, ^2^*J*_CF_ = 21 Hz, 2 × CH, Ar), 114.84 (CH, Ar), 100.74 (CH, Ar), 69.17 (CH_2_), 34.68 (CH_2_), 29.05 (CH_2_). Anal. calcd: C 73.59%, H 4.83%, N 3.73%. Found: C 73.58%, H 4.83%, N 3.47%.

#### (*E*)-7-(4-Chlorophenethoxy)-3-(pyridin-3-ylmethylene)chroman-4-one (5h)

Prepared from 7-(4-chlorophenethoxy)chroman-4-one (4h) (0.08 g, 0.26 mmol). The product was eluted with EtOAc to give the product (5h) as a beige solid. Yield: 0.03 g (32%), m.p: 117–119 °C, TLC (EtOAc) *R*_f_ 0.31. ^1^H NMR (CDCl_3_): *δ* 8.58 (s, 1H, Ar), 8.50 (s, 1H, Ar), 8.13 (d, *J* = 9.0 Hz, 1H, Ar), 7.72 (d, *J* = 7.5 Hz, 1H, Ar), 7.66 (s, 1H, C=CH), 7.32 (d, *J* = 8.5 Hz, 2H, Ar), 7.28 (s, 1H, Ar), 7.24 (d, *J* = 8.5 Hz, 2H, Ar), 6.97 (d, *J* = 8.5 Hz, 1H, Ar), 6.80 (s, 1H, Ar), 4.25 (t, *J* = 7.0 Hz, 2H, CH_2_), 3.80 (s, 2H, CH_2_), 3.13 (t, *J* = 7.0 Hz, 2H, CH_2_).^13^CNMR (CDCl_3_): *δ* 176.54 (C=O), 163.10 (C, Ar), 158.24 (C, Ar), 152.54 (CH, Ar), 149.99 (CH, Ar), 147.76 (CH, Ar), 136.74 (C=CH), 136.14 (C, Ar), 134.65 (C, Ar), 132.62 (C, Ar), 130.33 (2 × CH, Ar), 128.73 (2 × CH, Ar), 127.36 (CH, Ar), 123.52 (CH, Ar), 123.42 (C, Ar), 117.87 (C, Ar), 114.85 (CH, Ar), 100.76 (CH, Ar), 68.92 (CH_2_), 34.83 (CH_2_), 29.09 (CH_2_). Anal. calcd: C 70.50%, H 4.63%, N 3.57%. Found: C 70.40%, H 4.80%, N 3.38%.

#### (*E*)-7-(4-Bromophenethoxy)-3-(pyridin-3-ylmethylene)chroman-4-one (5i)

Prepared from 7-(4-bromophenethoxy)chroman-4-one (4i) (0.2 g, 0.57 mmol). The product was eluted with EtOAc to give the product (5i) as a white solid. Yield: 0.115 g (46%), m.p: 100–102 °C, TLC (petroleum ether – EtOAc 1 : 1 v/v) *R*_f_ 0.15. ^1^H NMR (CDCl_3_): *δ* 8.58 (s, 1H, Ar), 8.5 (s, 1H, Ar), 8.12 (d, *J* = 9.0 Hz, 1H, Ar), 7.68 (dt, *J* = 1.8, 7.8 Hz, 1H, Ar), 7.65 (t, *J* = 1 Hz, 1H, C=CH), 7.47 (m, 2H, Ar), 7.25 (dd, *J* = 4.8, 7.7 Hz, 1H, Ar), 7.19 (m, 2H, Ar), 6.96 (dd, *J* = 2.4, 8.9 Hz, 1H, Ar), 6.79 (d, *J* = 2.4 Hz, 1H, Ar), 4.24 (t, *J* = 6.7 Hz, 2H, CH_2_), 3.80 (s, 2H, CH_2_), 3.12 (t, *J* = 6.7 Hz, 2H, CH_2_). ^13^C NMR (CDCl_3_): *δ* 176.48 (C=O), 163.14 (C, Ar), 158.20 (C, Ar), 152.61 (C=CH), 148.68 (CH, Ar), 146.44 (CH, Ar), 138.10 (CH, Ar), 136.63 (C, Ar), 135.43 (C, Ar), 131.68 (2 × CH, Ar), 130.70 (2 × CH, Ar), 127.31 (CH, Ar), 123.88 (CH, Ar), 123.03 (C, Ar), 120.66 (C, Ar), 117.83 (C, Ar), 114.92 (CH, Ar), 100.76 (CH, Ar), 68.83 (CH_2_), 34.88 (CH_2_), 29.17 (CH_2_). HPLC: 100%, RT = 4.49 min. HRMS (ESI, *m*/*z*): theoretical mass: 436.0543 [M(^79^Br) + H]^+^, 438.0523 [M(^81^Br) + H]^++^, observed mass: 436.0541 [M(^79^Br) + H]^+^, 438.0524 [M(^81^Br) + H]^+^.

#### (*E*)-7-(4-Methoxyphenethoxy)-3-(pyridin-3-ylmethylene)chroman-4-one (5j)

Prepared from 7-(4-methoxyphenethoxy)chroman-4-one (4j) (0.08 g, 0.26 mmol). The product was eluted with EtOAc to give the product (5j) as a white solid. Yield: 0.05 g (47%), m.p: 110–112 °C, TLC (EtOAc) *R*_f_ 0.34. ^1^H NMR (CDCl_3_): *δ* 8.58 (s, 1H, Ar), 8.48 (d, *J* = 5.0 Hz, 1H, Ar), 8.14 (d, *J* = 9.0 Hz, 1H, Ar), 7.68 (dd, *J* = 2.0, 7.5 Hz, 1H, Ar), 7.64 (s, 1H, C=CH), 7.24 (d, *J* = 5.0 Hz, 1H, Ar), 7.22 (m, 2H, Ar), 6.97 (dd, *J* = 2.5, 9.0 Hz, 1H, Ar), 6.89 (m, 2H, Ar), 6.80 (d, *J* = 2.5 Hz, 1H, Ar), 4.23 (t, *J* = 7.0 Hz, 2H, CH_2_), 3.81 (s, 3H, CH_3_), 3.80 (s, 2H, CH_2_), 3.09 (t, *J* = 7.0 Hz, 2H, CH_2_).^13^CNMR (CDCl_3_): *δ* 176.37 (C=O), 163.33 (C, Ar), 158.48 (C, Ar), 158.24 (C, Ar), 152.54 (CH, Ar), 149.82 (CH, Ar), 147.64 (CH, Ar), 136.82 (C=CH), 134.76 (C, Ar), 129.89 (2 × CH, Ar), 129.52 (C, Ar), 127.20 (CH, Ar), 123.55 (CH, Ar), 123.33 (C, Ar), 117.74 (C, Ar), 114.93 (CH, Ar), 114.05 (2 × CH, Ar), 100.72 (CH, Ar), 69.55 (CH_2_), 55.23 (CH_3_), 34.47 (CH_2_), 29.04 (CH_2_). Anal. calcd: C 74.40%, H 5.46%, N 3.61%. Found: C 74.23%, H 5.35%, N 3.50%.

#### (*E*)-7-(3-Phenylpropoxy)-3-(pyridin-3-ylmethylene)chroman-4-one (5k)

Prepared from 7-(3-phenylpropoxy)chroman-4-one (4k) (0.5 g, 1.77 mmol). The product was eluted with EtOAc to give the product (5k) as a white solid. Yield: 0.49 g (74%), m.p: 120–122 °C, TLC (petroleum ether – EtOAc 1 : 1 v/v) *R*_f_ 0.13. ^1^H NMR (CDCl_3_): *δ* 8.63 (s, 1H, Ar), 8.51 (s, 1H, Ar), 8.11 (d, *J* = 9 Hz, 1H, Ar), 7.87 (d, *J* = 7.8 Hz, 1H, Ar), 7.74 (s, 1H, C=CH), 7.36 (t, *J* = 6.3 Hz, 2H, Ar), 7.28 (m, 5H, Ar), 6.98 (dd, *J* = 2.3, 8.9 Hz, 1H, Ar), 6.79 (d, *J* = 2.1 Hz, 1H, Ar), 4.05 (t, *J* = 6.3 Hz, 2H, CH_2_), 3.84 (s, 2H, CH_2_), 2.84 (t, *J* = 7.4 Hz, 2H, CH_2_), 2.17 (quintet, *J* = 7.5 Hz, 2H, CH_2_). ^13^C NMR (CDCl_3_): *δ* 176.51 (C=O), 163.57 (C, Ar), 158.29 (C, Ar), 152.64 (CH, Ar), 148.17 (CH, Ar), 145.87 (CH, Ar), 140.98 (C, Ar), 138.62 (C=CH), 135.82 (C, Ar), 128.51 (2 × CH, Ar), 128.48 (2 × CH, Ar), 127.19 (CH, Ar), 126.12 (CH, Ar), 124.03 (CH, Ar), 122.83 (C, Ar), 117.64 (C, Ar), 115.08 (CH, Ar), 100.66 (CH, Ar), 67.53 (CH_2_), 31.97 (CH_2_), 30.44 (CH_2_), 29.21 (CH_2_). Anal. calcd: C 77.61%, H 5.70%, N 3.77%. Found: C 77.49%, H 5.82%, N 3.55%.

#### (*E*)-7-(3-(4-Fluorophenyl)propoxy)-3-(pyridin-3-ylmethylene)chroman-4-one (5l)

Prepared from 7-(3-(4-fluorophenyl)propoxy)chroman-4-one (4l) (0.25 g, 0.83 mmol). The product was eluted with EtOAc to give the product (5l) as a white solid. Yield: 0.28 g (87%), m.p: 126–128 °C, TLC (petroleum ether – EtOAc 1 : 1 v/v) *R*_f_ 0.13. ^1^H NMR (CDCl_3_): *δ* 8.58 (s, 1H, Ar), 8.51 (d, *J* = 3.9 Hz, 1H, Ar), 8.13 (d, *J* = 8.9 Hz, 1H, Ar), 7.69 (ddd, *J* = 1.7, 2.4, 7.8 Hz, 1H, Ar), 7.66 (t, *J* = 1.0 Hz, 1H, C=CH), 7.25 (m, 1H, Ar), 7.19 (m, 2H, Ar), 7.00 (m, 2H, Ar), 6.98 (dd, *J* = 2.5, 8.9 Hz, 1H, Ar), 6.78 (d, *J* = 2.4 Hz, 1H, Ar), 4.03 (t, *J* = 6.2 Hz, 2H, CH_2_), 3.81 (s, 2H, CH_2_), 2.82 (t, *J* = 7.5 Hz, 2H, CH_2_), 2.15 (m, 2H, CH_2_). ^13^C NMR (CDCl_3_): *δ* 176.49 (C=O), 163.50 (C, Ar), 161.39 (d, ^1^*J*_CF_ = 244.2 Hz, C–F, Ar), 158.28 (C, Ar), 152.50 (CH, Ar), 152.67 (CH, Ar), 147.91 (CH, Ar), 145.62 (C=CH), 138.89 (CH, Ar), 136.56 (d, ^4^*J*_CF_ = 2.9 Hz, C, Ar), 129.81 (d, ^3^*J*_CF_ = 8.1 Hz, 2 × CH, Ar), 127.23 (CH, Ar), 124.10 (CH, Ar), 122.78 (C, Ar) 117.68 (C, Ar), 115.25 (d, ^2^*J*_CF_ = 21.0 Hz, 2 × CH, Ar), 115.03 (CH, Ar), 100.65 (CH, Ar), 67.34 (CH_2_), 31.18 (CH_2_), 30.56 (CH_2_), 29.23 (CH_2_). ^19^F NMR (CDCl_3_): *δ* −117.23 (Ar–F). Anal. calcd: C 74.02%, H 5.18%, N 3.60%. Found: C 74.12%, H 5.44%, N 3.57%.

#### (*E*)-7-(3-(4-Chlorophenyl)propoxy)-3-(pyridin-3-ylmethylene)chroman-4-one (5m)

Prepared from 7-(3-(4-chlorophenyl)propoxy)chroman-4-one (4m) (0.5 g, 1.58 mmol). The product was eluted with EtOAc to give the product (5m) as a white solid. Yield: 0.22 g (35%), m.p: 129–131 °C, TLC (petroleum ether – EtOAc 1 : 1 v/v) *R*_f_ 0.13. ^1^H NMR (CDCl_3_): *δ* 8.64 (s, 1H, Ar), 8.51 (s, 1H, Ar), 8.11 (d, *J* = 8.9 Hz, 1H, Ar), 7.87 (d, *J* = 7.8 Hz, 1H, Ar), 7.76 (s, 1H, C=CH), 7.37 (t, *J* = 6 Hz, 2H, Ar), 7.27 (d, *J* = 8.4 Hz, 2H, Ar), 7.15 (d, *J* = 8.3 Hz, 2H, Ar), 6.97 (dd, *J* = 2.2, 8.9 Hz, 1H, Ar), 6.78 (d, *J* = 2.1, Hz, 1H, Ar), 4.03 (t, *J* = 6.2 Hz, 2H, CH_2_), 3.84 (s, 2H, CH_2_), 2.81 (t, *J* = 7.5 Hz, 2H, CH_2_), 2.14 (quintet, *J* = 7.5 Hz, 2H, CH_2_). ^13^C NMR (CDCl_3_): *δ* 176.50 (C=O), 163.45 (C, Ar), 158.27 (C, Ar), 152.67 (CH, Ar), 148.11 (CH, Ar), 145.82 (CH, Ar), 139.40 (C, Ar), 138.67 (C=CH), 134.54 (C, Ar), 131.87 (C, Ar), 129.82 (2 × CH, Ar), 128.61 (2 × CH, Ar), 127.24 (CH, Ar), 124.06 (CH, Ar), 122.85 (C, Ar), 117.71 (C, Ar), 115.00 (CH, Ar), 100.65 (CH, Ar), 67.27 (CH_2_), 31.35 (CH_2_), 30.36 (CH_2_), 29.21 (CH_2_). Anal. calcd: C 71.02%, H 4.97%, N 3.45%. Found: C 71.26%, H 5.13%, N 3.10%.

#### (*E*)-7-(3-(4-bromophenyl)propoxy)-3-(pyridin-3-ylmethylene)chroman-4-one (5n)

Prepared from 7-(3-(4-bromophenyl)propoxy)chroman-4-one (4n) (0.355 g, 0.98 mmol). The product was eluted with EtOAc to give the product (5n) as a white solid. Yield: 0.17 g (39%), m.p: 120–122 °C, TLC (petroleum ether – EtOAc 1 : 1 v/v) *R*_f_ 0.13. ^1^H NMR (CDCl_3_): *δ* 8.58 (d, *J* = 1.5 Hz, 1H, Ar), 8.49 (d, *J* = 1.2, 4.7 Hz, 1H, Ar), 8.13 (d, *J* = 8.9 Hz, 1H, Ar), 7.69 (ddd, *J* = 1.7, 2.2, 7.8 Hz, 1H, Ar), 7.66 (t, *J* = 1.1 Hz, 1H, C=CH), 7.43 (m, 2H, Ar), 7.25 (ddd, *J* = 0.6, 4.8, 7.8 Hz, 1H, Ar), 7.10 (m, 2H, Ar), 6.98 (dd, *J* = 2.4, 8.9 Hz, 1H, Ar), 6.77 (d, *J* = 2.3, Hz, 1H, Ar), 4.03 (t, *J* = 6.2 Hz, 2H, CH_2_), 3.81 (s, 2H, CH_2_), 2.81 (t, *J* = 7.5 Hz, 2H, CH_2_), 2.14 (m, 2H, CH_2_). ^13^C NMR (CDCl_3_): *δ* 176.47 (C=O), 163.46 (C, Ar), 158.27 (C, Ar), 152.71 (CH, Ar), 147.65 (CH, Ar), 145.35 (CH, Ar), 139.92 (C, Ar), 139.17 (C=CH), 136.11 (C, Ar), 131.57 (2 × CH, Ar), 130.23 (2 × CH, Ar), 127.24 (CH, Ar), 124.18 (CH, Ar), 122.72 (C, Ar), 119.89 (C, Ar), 117.70 (C, Ar), 115.02 (CH, Ar), 100.66 (CH, Ar), 67.26 (CH_2_), 31.42 (CH_2_), 30.29 (CH_2_), 29.25 (CH_2_). Anal. calcd: C 64.01%, H 4.48%, N 3.11%. Found: C 64.33%, H 4.57%, N 3.09%.

#### (*E*)-7-(3-(4-Methoxyphenyl)propoxy)-3-(pyridin-3-ylmethylene)chroman-4-one (5o)

Prepared from 7-(3-(4-methoxyphenyl)propoxy)chroman-4-one (4o) (0.5 g, 1.6 mmol). The product was eluted with EtOAc to give the product (5o) as a white solid. Yield: 0.37 g (58%), m.p: 115–117 °C, TLC (petroleum ether – EtOAc 1 : 1 v/v) *R*_f_ 0.25. ^1^H NMR (CDCl_3_): *δ* 8.63 (s, 1H, Ar), 8.50 (d, *J* = 4.3 Hz, 1H, Ar), 8.11 (d, *J* = 8.9 Hz, 1H, Ar), 7.86 (d, *J* = 7.9 Hz, 1H, Ar), 7.74 (s, 1H, C=CH), 7.35 (t, *J* = 6.4 Hz, 2H, Ar), 7.13 (d, *J* = 8.6 Hz, 2H, Ar), 6.98 (dd, *J* = 2.3, 8.9 Hz, 1H, Ar), 6.85 (d, *J* = 8.6 Hz, 2H, Ar), 6.79 (d, *J* = 2.3 Hz, 1H, Ar), 4.03 (t, *J* = 6.3 Hz, 2H, CH_2_), 3.84 (s, 2H, CH_2_), 3.8 (s, 3H, CH_3_), 2.78 (t, *J* = 7.5 Hz, 2H, CH_2_), 2.13 (quintet, *J* = 7.1 Hz, 2H, CH_2_). ^13^C NMR (CDCl_3_): *δ* 176.52 (C=O), 163.6 (C, Ar), 158.28 (C, Ar), 157.97 (C, Ar), 152.64 (CH, Ar), 148.24 (CH, Ar), 145.94 (CH, Ar), 138.55 (C=CH), 135.82 (C, Ar), 132.98 (C, Ar), 129.38 (2 × CH, Ar), 127.18 (2 × CH, Ar), 124.00 (CH, Ar), 122.85 (C, Ar), 117.62 (CH, Ar), 115.06 (CH, Ar), 113.91 (CH, Ar), 100.65 (CH, Ar), 67.50 (CH_2_), 55.26 (CH_3_), 31.02 (CH_2_), 30.64 (CH_2_), 29.20 (CH_2_). Anal. calcd: C 74.80%, H 5.77%, N 3.49%. Found: C 74.71%, H 5.61%, N 3.34%.

#### (*E*)-6-Phenethoxy-2-(pyridin-3-ylmethylene)-3,4-dihydronaphthalen-1(2*H*)-one (8a)

Prepared from 6-phenethoxy-3,4-dihydronaphthalen-1(2*H*)-one (7a) (0.25 g, 0.93 mmol). The product was eluted with petroleum ether – EtOAc 40 : 60 v/v to give the product (8a) as a beige solid. Yield: 0.14 g (42%), m.p: 90–92 °C, TLC (petroleum ether – EtOAc 1 : 1 v/v) *R*_f_ 0.26. ^1^H NMR (CDCl_3_): *δ* 8.71 (s, 1H, Ar), 8.59 (s, 1H, Ar), 8.13 (d, *J* = 8.5 Hz, 1H, Ar), 7.78 (s, 1H, C=CH), 7.74 (d, *J* = 8.0 Hz, 1H, Ar), 7.37 (m, 2H, Ar), 7.34 (s, 1H, Ar), 7.29 (m, 3H, Ar), 6.90 (d, *J* = 8.5 Hz, 1H, Ar), 6.73 (s, 1H, Ar), 4.27 (t, *J* = 7.0 Hz, 2H, CH_2_), 3.15 (t, *J* = 7.0 Hz, 2H, CH_2_), 3.10 (t, *J* = 6.0 Hz, 2H, CH_2_), 2.94 (t, *J* = 6.0 Hz, 2H, CH_2_). ^13^C NMR (CDCl_3_): *δ* 186.14 (C=O), 163.09 (C, Ar), 150.56 (CH, Ar), 149.09 (CH, Ar), 145.66 (C, Ar), 137.80 (C, Ar), 137.70 (C, Ar), 136.71 (C=CH), 131.94 (C, Ar), 131.90 (CH, Ar), 130.91 (CH, Ar), 128.99 (2 × CH, Ar), 128.59 (2 × CH, Ar), 126.75 (C, Ar), 126.69 (CH, Ar), 123.29 (CH, Ar), 113.99 (CH, Ar), 112.91 (CH, Ar), 68.89 (CH_2_), 35.65 (CH_2_), 29.19 (CH_2_), 27.24 (CH_2_). Anal. calcd: C 81.10%, H 5.95%, N 3.94%. Found: C 80.93%, H 5.90%, N 3.86%.

#### (*E*)-6-(4-Fluorophenethoxy)-2-(pyridin-3-ylmethylene)-3,4-dihydronaphthalen-1(2*H*)-one (8b)

Prepared from 6-(4-fluorophenethoxy)-3,4-dihydronaphthalen-1(2*H*)-one (7b) (0.25 g, 0.88 mmol). The product was eluted with petroleum ether – EtOAc 40 : 60 v/v to give the product (8b) as a light brown solid. Yield: 0.15 g (46%), m.p: 95–97 °C, TLC (petroleum ether – EtOAc 1 : 1 v/v) *R*_f_ 0.30. ^1^H NMR (CDCl_3_): *δ* 8.73 (s, 1H, Ar), 8.63 (s, 1H, Ar), 8.12 (d, *J* = 9.0 Hz, 1H, Ar), 7.90 (d, *J* = 8.0 Hz, 1H, Ar), 7.76 (s, 1H, C=CH), 7.54 (t, *J* = 6.0 Hz, 1H, Ar), 7.26 (t, *J* = 8.5 Hz, 2H, Ar), 7.0 (t, *J* = 8.5 Hz, 2H, Ar), 6.90 (d, *J* = 9.0 Hz, 1H, Ar), 6.72 (s, 1H, Ar), 4.25 (t, *J* = 7.0 Hz, 2H, CH_2_), 3.12 (t, *J* = 7.0 Hz, 2H, CH_2_), 3.09 (t, *J* = 6.5 Hz, 2H, CH_2_), 2.95 (t, *J* = 6.5 Hz, 2H, CH_2_). ^19^F NMR (CDCl_3_): *δ* −116.41 (Ar–F). ^13^C NMR (CDCl_3_): *δ* 185.65 (C=O), 163.16 (C, Ar), 162.76 and 160.81 (d, ^1^*J*_CF_ = 245.2 Hz, C–F, Ar), 147.60 (CH, Ar), 146.26 (CH, Ar), 145.60 (C, Ar), 139.33 (C=CH), 138.96 (C, Ar), 138.96 (C, Ar), 133.52 (d, ^4^*J*_CF_ = 3.3 Hz, C, Ar), 131.03 (CH, Ar), 130.45 (d, ^3^*J*_CF_ = 8.0 Hz, 2 × CH, Ar), 130.34 (CH, Ar), 126.60 (C, Ar), 124.35 (CH, Ar), 115.47 and 115.30 (d, ^2^*J*_CF_ = 21.2 Hz, 2 × CH, Ar), 114.11 (CH, Ar), 112.94 (CH, Ar), 68.82 (CH_2_), 34.82 (CH_2_), 29.04 (CH_2_), 27.25 (CH_2_). Anal. calcd: C 77.19%, H 5.40%, N 3.75%. Found: C 76.98%, H 5.29%, N 3.69%.

#### (*E*)-6-(4-Chlorophenethoxy)-2-(pyridin-3-ylmethylene)-3,4-dihydronaphthalen-1(2*H*)-one (8c)

Prepared from 6-(4-chlorophenethoxy)-3,4-dihydronaphthalen-1(2*H*)-one (7c) (0.22 g, 0.73 mmol). The product was eluted with petroleum ether – EtOAc 40 : 60 v/v to give the product (8c) as a biege solid. Yield: 0.11 g (39%), m.p: 86–88 °C, TLC (petroleum ether – EtOAc 1 : 1 v/v) *R*_f_ 0.23. ^1^H NMR (CDCl_3_): *δ* 8.73 (s, 1H, Ar), 8.64 (s, 1H, Ar), 8.12 (d, *J* = 9.0 Hz, 1H, Ar), 7.92 (d, *J* = 8.0 Hz, 1H, Ar), 7.76 (s, 1H, C=CH), 7.55 (t, *J* = 6.0 Hz, 1H, Ar), 7.31 (d, *J* = 8.0 Hz, 2H, Ar), 7.24 (d, *J* = 8.0 Hz, 2H, Ar), 6.90 (d, *J* = 9.0 Hz, 1H, Ar), 6.72 (s, 1H, Ar), 4.25 (t, *J* = 7.0 Hz, 2H, CH_2_), 3.12 (t, *J* = 7.0 Hz, 2H, CH_2_), 3.09 (t, *J* = 6.5 Hz, 2H, CH_2_), 2.95 (t, *J* = 6.5 Hz, 2H, CH_2_). ^13^C NMR (CDCl_3_): *δ* 185.62 (C=O), 163.11 (C, Ar), 147.48 (CH, Ar), 146.14 (CH, Ar), 145.59 (C, Ar), 139.44 (CH, Ar), 139.0 (C, Ar), 136.34 (C, Ar), 133.27 (C=CH, Ar), 132.56 (C, Ar), 131.04 (CH, Ar), 130.33 (2 × CH, Ar), 130.28 (CH, Ar), 128.69 (2 × CH, Ar), 126.63 (C, Ar), 124.37 (CH, Ar), 114.10 (CH, Ar), 112.96 (CH, Ar), 68.57 (CH_2_), 34.97 (CH_2_), 29.03 (CH_2_), 27.25 (CH_2_). Anal. calcd: C 73.94%, H 5.17%, N 3.59%. Found: C 73.74%, H 5.01%, N 3.48%.

#### (*E*)-6-(4-Bromophenethoxy)-2-(pyridin-3-ylmethylene)-3,4-dihydronaphthalen-1(2*H*)-one (8d)

Prepared from 6-(4-bromophenethoxy)-3,4-dihydronaphthalen-1(2*H*)-one (7d) (0.29 g, 0.84 mmol). The product was eluted with petroleum ether – EtOAc 40 : 60 v/v to give the product (8d) as a biege solid. Yield: 0.09 g (26%), m.p: 90–92 °C, TLC (petroleum ether – EtOAc 1 : 1 v/v) *R*_f_ 0.23. ^1^H NMR (CDCl_3_): *δ* 8.71 (s, 1H, Ar), 8.60 (s, 1H, Ar), 8.14 (d, *J* = 9.0 Hz, 1H, Ar), 7.78 (s, 1H, C=CH), 7.75 (d, *J* = 8.0 Hz, 1H, Ar), 7.48 (d, *J* = 8.0 Hz, 2H, Ar), 7.39 (dd, *J* = 5.0, 8.0 Hz, 1H, Ar), 7.20 (d, *J* = 8.5 Hz, 2H, Ar), 6.90 (dd, *J* = 2.5, 9.0 Hz, 1H, Ar), 6.71 (d, *J* = 2.5 Hz, 1H, Ar), 4.25 (t, *J* = 6.5 Hz, 2H, CH_2_), 3.10 (t, *J* = 6.5 Hz, 2H, CH_2_), 3.09 (t, *J* = 6.5 Hz, 2H, CH_2_), 2.94 (t, *J* = 6.5 Hz, 2H, CH_2_). ^13^C NMR (CDCl_3_): *δ* 186.14 (C=O), 162.90 (C, Ar), 150.48 (CH, Ar), 149.05 (CH, Ar), 145.68 (C, Ar), 137.66 (C, Ar), 136.91 (C, Ar), 136.81 (C=CH), 131.96 (CH, Ar), 131.93 (C, Ar), 131.65 (2 × CH, Ar), 130.95 (CH, Ar), 130.74 (2 × CH, Ar), 126.84 (C, Ar), 123.34 (CH, Ar), 120.58 (C, Ar), 113.90 (CH, Ar), 112.92 (CH, Ar), 68.43 (CH_2_), 35.05 (CH_2_), 29.17 (CH_2_), 27.23 (CH_2_). Anal. calcd: C 66.37%, H 4.64%, N 3.22%. Found: C 66.63%, H 4.74%, N 3.20%.

#### (*E*)-6-(4-Methoxyphenethoxy)-2-(pyridin-3-ylmethylene)-3,4-dihydronaphthalen-1(2*H*)-one (8e)

Prepared from 6-(4-methoxyphenethoxy)-3,4-dihydronaphthalen-1(2*H*)-one (7e) (0.2 g, 0.67 mmol). The product was eluted with petroleum ether – EtOAc 40 : 60 v/v to give the product (8e) as a brown oil. Yield: 0.08 g (31%), TLC (petroleum ether – EtOAc 1 : 1 v/v) *R*_f_ 0.24. ^1^H NMR (CDCl_3_): *δ* 8.70 (s, 1H, Ar), 8.58 (s, 1H, Ar), 8.12 (d, *J* = 9.0 Hz, 1H, Ar), 7.77 (s, 1H, C=CH), 7.74 (d, *J* = 8.0 Hz, 1H, Ar), 7.37 (t, *J* = 6.0 Hz, 1H, Ar), 7.23 (d, *J* = 8.0 Hz, 2H, Ar), 6.89 (m, 2H, Ar), 6.88 (m, 1H, Ar), 6.72 (s, 1H, Ar), 4.23 (t, *J* = 7.0 Hz, 2H, CH_2_), 3.81 (s, 3H, CH_3_), 3.09 (m, 2H, CH_2_), 3.08 (t, *J* = 6.5 Hz, 2H, CH_2_), 2.94 (t, *J* = 6.5 Hz, 2H, CH_2_). ^13^C NMR (CDCl_3_): *δ* 186.14 (C=O), 163.14 (C, Ar), 158.42 (C, Ar), 150.52 (CH, Ar), 149.06 (CH, Ar), 145.66 (C, Ar), 137.73 (C, Ar), 136.73 (C=CH), 131.95 (C, Ar), 131.87 (CH, Ar), 130.90 (CH, Ar), 129.96 (2 × CH, Ar), 129.81 (C, Ar), 126.70 (CH, Ar), 123.30 (CH, Ar), 114.02 (2 × CH, Ar), 114.02 (CH, Ar), 112.91 (CH, Ar), 68.57 (CH_2_), 55.29 (CH_3_), 34.97 (CH_2_), 29.03 (CH_2_), 27.25 (CH_2_). HPLC: 100%, RT = 4.81 min. HRMS (ESI, *m*/*z*): theoretical mass: 386.1710 [M + H]^+^, observed mass: 386.1710 [M + H]^+^.

#### (*E*)-6-(3-Phenylpropoxy)-2-(pyridin-3-ylmethylene)-3,4-dihydronaphthalen-1(2*H*)-one (8f)

Prepared from 6-phenylpropoxy-3,4-dihydronaphthalen-1(2*H*)-one (7f) (0.5 g, 1.78 mmol). The product was eluted with petroleum ether – EtOAc 40 : 60 v/v to give the product (8f) as a biege solid. Yield: 0.21 g (32%), m.p: 113–115 °C, TLC (petroleum ether – EtOAc 1 : 1 v/v) *R*_f_ 0.32. ^1^H NMR (CDCl_3_): *δ* 8.73 (s, 1H, Ar), 8.63 (s, 1H, Ar), 8.13 (d, *J* = 8.5 Hz, 1H, Ar), 7.87 (d, *J* = 8.0 Hz, 1H, Ar), 7.78 (s, 1H, C=CH), 7.49 (s, 1H, Ar), 7.32 (t, *J* = 7.0 Hz, 2H, Ar), 7.24 (m, 2H, Ar), 7.23 (m, 1H, Ar), 6.91 (d, *J* = 8.5 Hz, 1H, Ar), 6.72 (s, 1H, Ar), 4.06 (t, *J* = 6.0 Hz, 2H, CH_2_), 3.10 (t, *J* = 6.0 Hz, 2H, CH_2_), 2.96 (t, *J* = 6.0 Hz, 2H, CH_2_), 2.85 (t, *J* = 7.5 Hz, 2H, CH_2_), 2.16 (quintet, *J* = 6.5 Hz, 2H, CH_2_). ^13^C NMR (CDCl_3_): *δ* 185.75 (C=O), 163.48 (C, Ar), 158.42 (C, Ar), 148.10 (CH, Ar), 146.71 (CH, Ar), 145.61 (C, Ar), 141.14 (C, Ar), 138.89 (C=CH), 138.84 (C, Ar), 130.98 (CH, Ar), 130.51 (CH, Ar), 128.51 (4 × CH, Ar), 126.48 (C, Ar), 126.09 (CH, Ar), 124.15 (CH, Ar), 114.17 (CH, Ar), 112.87 (CH, Ar), 67.14 (CH_2_), 32.02 (CH_2_), 30.59 (CH_2_), 29.09 (CH_2_), 27.25 (CH_2_). Anal. calcd: C 81.27%, H 6.27%, N 3.79%. Found: C 80.96%, H 5.99%, N 3.75%.

#### (*E*)-6-(3-(4-Fluorophenyl)propoxy)-2-(pyridin-3-ylmethylene)-3,4-dihydronaphthalen-1(2*H*)-one (8g)

Prepared from 6-(3-(4-fluorophenyl)propoxy)-3,4-dihydronaphthalen-1(2*H*)-one (7g) (0.56 g, 1.8 mmol). The product was eluted with petroleum ether – EtOAc 40 : 60 v/v to give the product (8g) as an orange solid. Yield: 0.26 g (38%), m.p: 88–90 °C, TLC (petroleum ether – EtOAc 1 : 1 v/v) *R*_f_ 0.37. ^1^H NMR (CDCl_3_): *δ* 8.74 (s, 1H, Ar), 8.64 (s, 1H, Ar), 8.14 (d, *J* = 8.5 Hz, 1H, Ar), 7.89 (d, *J* = 7.5 Hz, 1H, Ar), 7.77 (s, 1H, C=CH), 7.51 (s, 1H, Ar), 7.18 (dd, *J* = 5.5, 8.5 Hz, 2H, Ar), 6.98 (t, *J* = 8.5 Hz, 2H, Ar), 6.91 (dd, *J* = 2.5, 8.5 Hz, 1H, Ar), 6.72 (d, *J* = 2.5 Hz, 1H, Ar), 4.04 (t, *J* = 6.5 Hz, 2H, CH_2_), 3.10 (t, *J* = 6.0 Hz, 2H, CH_2_), 2.96 (t, *J* = 6.0 Hz, 2H, CH_2_), 2.82 (t, *J* = 7.5 Hz, 2H, CH_2_), 2.13 (quintet, *J* = 6.5 Hz, 2H, CH_2_). ^19^F NMR (CDCl_3_): *δ* −117.31 (Ar-F). ^13^C NMR (CDCl_3_): *δ* 185.81 (C=O), 163.37 (C, Ar), 162.36 and 160.42 (d, ^1^*J*_CF_ = 244.0 Hz, C–F, Ar), 148.39 (CH, Ar), 147.01 (CH, Ar), 145.65 (C, Ar), 138.65 (C=CH), 138.63 (C, Ar), 138.63 (C, Ar), 136.74 (d, ^4^*J*_CF_ = 3.2 Hz, C, Ar), 130.99 (CH, Ar), 130.99 (CH, Ar), 130.74 (CH, Ar), 129.87 (d, ^3^*J*_CF_ = 7.8 Hz, 2 × CH, Ar), 126.55 (C, Ar), 115.33 and 115.16 (d, ^2^*J*_CF_ = 21.3 Hz, 2 × CH, Ar), 114.11 (CH, Ar), 112.83 (CH, Ar), 66.93 (CH_2_), 31.22 (CH_2_), 30.72 (CH_2_), 29.10 (CH_2_), 27.26 (CH_2_). Anal. calcd: C 77.50%, H 5.72%, N 3.61%. Found: C 77.38%, H 5.71%, N 3.63%.

#### (*E*)-6-(3-(4-Chlorophenyl)propoxy)-2-(pyridin-3-ylmethylene)-3,4-dihydronaphthalen-1(2*H*)-one (8h)

Prepared from 6-(3-(4-chlorophenyl)propoxy)-3,4-dihydronaphthalen-1(2*H*)-one (7h) (0.5 g, 1.6 mmol). The product was eluted with petroleum ether – EtOAc 40 : 60 v/v to give the product (8h) as a white solid. Yield: 0.22 g (34%), m.p: 97–99 °C, TLC (petroleum ether – EtOAc 1 : 1 v/v) *R*_f_ 0.26. ^1^H NMR (CDCl_3_): *δ* 8.73 (s, 1H, Ar), 8.63 (s, 1H, Ar), 8.14 (d, *J* = 8.5 Hz, 1H, Ar), 7.89 (d, *J* = 8.0 Hz, 1H, Ar), 7.77 (s, 1H, C=CH), 7.52 (t, *J* = 6.0 Hz, 1H, Ar), 7.28 (d, *J* = 7.0 Hz, 2H, Ar), 7.16 (d, *J* = 8.0 Hz, 2H, Ar), 6.90 (d, *J* = 9.0 Hz, 1H, Ar), 6.71 (s, 1H, Ar), 4.04 (t, *J* = 6.0 Hz, 2H, OCH_2_), 3.10 (t, *J* = 6.0 Hz, 2H, CH_2_), 2.96 (t, *J* = 6.0 Hz, 2H, CH_2_), 2.82 (t, *J* = 7.5 Hz, 2H, CH_2_), 2.13 (quintet, *J* = 6.5 Hz, 2H, CH_2_). ^13^C NMR (CDCl_3_): *δ* 185.70 (C=O), 163.35 (C, Ar), 147.98 (CH, Ar), 146.62 (CH, Ar), 145.62 (C, Ar), 139.56 (C, Ar), 138.95 (C=CH), 138.83 (C, Ar), 138.83 (C, Ar), 131.86 (C, Ar), 131.00 (CH, Ar), 130.51 (CH, Ar), 129.88 (CH, Ar), 129.85 (2 × CH, Ar), 128.60 (2 × CH, Ar), 126.57 (C, Ar), 114.13 (CH, Ar), 112.84 (CH, Ar), 66.89 (CH_2_), 31.40 (CH_2_), 30.50 (CH_2_), 29.08 (CH_2_), 27.26 (CH_2_). Anal. calcd: C 74.34%, H 5.49%, N 3.47%. Found: C 74.12%, H 5.26%, N 3.40%.

#### (*E*)-6-(3-(4-Bromophenyl)propoxy)-2-(pyridin-3-ylmethylene)-3,4-dihydronaphthalen-1(2*H*)-one (8i)

Prepared from 6-(3-(4-bromophenyl)propoxy)-3,4-dihydronaphthalen-1(2*H*)-one (7i) (0.82 g, 2.28 mmol). The product was eluted with petroleum ether – EtOAc 40 : 60 v/v to give the product (8i) as a beige solid. Yield: 0.47 g (47%), m.p: 102–104 °C, TLC (petroleum ether – EtOAc 1 : 1 v/v) *R*_f_ 0.36. ^1^H NMR (CDCl_3_): *δ* 8.71 (s, 1H, Ar), 8.60 (s, 1H, Ar), 8.14 (d, *J* = 8.5 Hz, 1H, Ar), 7.79 (s, 1H, C=CH), 7.75 (d, *J* = 8.0 Hz, 1H, Ar), 7.44 (d, *J* = 8.0 Hz, 2H, Ar), 7.39 (dd, *J* = 5.0, 7.5 Hz, 1H, Ar), 7.12 (d, *J* = 8.0 Hz, 2H, Ar), 6.91 (dd, *J* = 2.5, 8.5 Hz, 1H, Ar), 6.71 (d, *J* = 2.5 Hz, 1H, Ar), 4.04 (t, *J* = 6.5 Hz, 2H, OCH_2_), 3.11 (t, *J* = 6.5 Hz, 2H, CH_2_), 2.95 (t, *J* = 6.5 Hz, 2H, CH_2_), 2.81 (t, *J* = 7.5 Hz, 2H, CH_2_), 2.13 (quintet, *J* = 7.0 Hz, 2H, CH_2_). ^13^C NMR (CDCl_3_): *δ* 186.16 (C=O), 163.19 (C, Ar), 150.49 (CH, Ar), 149.05 (CH, Ar), 145.71 (C, Ar), 140.12 (C, Ar), 137.71 (C=CH), 136.80 (C, Ar), 136.62 (C, Ar), 131.95 (C, Ar), 131.91 (CH, Ar), 131.56 (CH, Ar), 130.93 (2 × CH, Ar), 130.29 (2 × CH, Ar), 126.72 (C, Ar), 123.34 (CH, Ar), 119.84 (C, Ar), 113.98 (CH, Ar), 112.80 (CH, Ar), 66.80 (CH_2_), 31.47 (CH_2_), 30.46 (CH_2_), 29.20 (CH_2_), 27.24 (CH_2_). Anal. calcd: C 66.97%, H 4.95%, N 3.12%. Found: C 67.25%, H 4.90%, N 3.16%.

#### (*E*)-6-(3-(4-Methoxyphenyl)propoxy)-2-(pyridin-3-ylmethylene)-3,4-dihydronaphthalen-1(2*H*)-one (8j)

Prepared from 6-(3-(4-methoxyphenyl)propoxy)-3,4-dihydronaphthalen-1(2*H*)-one (7j) (0.45 g, 1.45 mmol). The product was eluted with petroleum ether – EtOAc 40 : 60 v/v to give the product (8j) as a white solid. Yield: 0.20 g (34%), m.p: 88–90 °C, TLC (petroleum ether – EtOAc 1 : 1 v/v) *R*_f_ 0.25. ^1^H NMR (CDCl_3_): *δ* 8.73 (s, 1H, Ar), 8.63 (s, 1H, Ar), 8.14 (d, *J* = 8.5 Hz, 1H, Ar), 7.86 (d, *J* = 8.0 Hz, 1H, Ar), 7.77 (s, 1H, C=CH), 7.49 (s, 1H, Ar), 7.15 (d, *J* = 7.5 Hz, 2H, Ar), 6.91 (d, *J* = 8.5 Hz, 1H, Ar), 6.87 (d, *J* = 8.0 Hz, 2H, Ar), 6.71 (s, 1H, Ar), 4.04 (t, *J* = 5.5 Hz, 2H, CH_2_), 3.81 (s, 3H, CH_3_), 3.10 (t, *J* = 6.5 Hz, 2H, CH_2_), 2.96 (t, *J* = 5.0 Hz, 2H, CH_2_), 2.79 (t, *J* = 7.5 Hz, 2H, CH_2_), 2.12 (t, *J* = 6.5 Hz, 2H, CH_2_). ^13^C NMR (CDCl_3_): *δ* 185.79 (C=O), 163.48 (C, Ar), 157.99 (C, Ar), 148.48 (CH, Ar), 147.07 (CH, Ar), 145.61 (C, Ar), 138.67 (C, Ar), 138.67 (C, Ar), 138.52 (C=CH), 133.15 (C, Ar), 130.96 (CH, Ar), 130.70 (CH, Ar), 129.40 (2 × CH, Ar), 126.50 (C, Ar), 124.03 (CH, Ar), 114.15 (CH, Ar), 113.93 (2 × CH, Ar), 112.87 (CH, Ar), 67.11 (CH_2_), 55.28 (CH_3_), 31.07 (CH_2_), 30.79 (CH_2_), 29.11 (CH_2_), 27.27 (CH_2_). Anal. calcd: C 78.17%, H 6.31%, N 3.50%. Found: C 78.0%, H 6.29%, N 3.47%.

### Biological assays

Antimycobacterial activity was determined using either the Spot culture growth inhibition (SPOTi) assay^28^ or the resazurin microtiter assay (REMA) assay^30^ with full details provided in the SI. The different *Mtb* strains used are detailed in Table S3.

Full details for the UV-vis CYP121A1 spectral binding assay^5,27^ for *K*_D_ determination are reported in the SI.

#### Bacterial minimum bactericidal concentrations (MBC_95_) determination

Stock solutions of the tested compounds were prepared in sterile dimethyl sulfoxide (DMSO), then diluted in 7H9OPALPen^1^Cyc^10^(Kan^25^) to obtain a final drug concentration range of 200–0.2 μM. A suspension of the test *Mycobacterium* was cultured in 7H9OPALPen^1^Cyc^10^(Kan^25^) containing 0.05% v/v Tween^80^ for one week at 37 °C, 5% CO_2_. The bacterial suspension was adjusted to 0.5 McFarland and diluted in 7H9OPALPen^1^Cyc^10^(Kan^25^) 1 : 25. 100 μL of the inoculum was added to each well of a 96-well microplate together with 100 μL of the compound titration. The plate was incubated at 37 °C, 5% CO_2_ for 5 days. MBC_95_ were determined *via* REMA assessment of regrowth and CFU mL^−1^. 10 μL of the MIC plate was transferred to a corresponding well of a 96-well microplate containing 190 μL of fresh 7H9OPALPen^1^Cyc^10^(Kan^25^) containing 0.05% v/v Tween^80^ and re-incubated for 5 days at 37 °C, 5% CO_2_. After incubation, 10.5 μL 0.1% (w/v) sterile resazurin (solubilised in sterile PBS containing 0.02% v/v Tween^80^) was added. Reduced resazurin was detected using fluorescence (Ex/Em 530/590 nm) in a FLUOstar Optima, BMG Labtech. A further 20 μL of each well of the MIC plates were 10-fold serial dilute in 7H9OPALPen^1^Cyc^10^(Kan^25^) containing 0.05% v/v Tween^80^ to 10^−6^. 5 μL of the resulting dilution series were plated on 7H11OPALPen^1^Cyc^10^(Kan^25^) and incubated for 3 weeks at 37 °C, 5% CO_2_. Colonies were enumerated. MBCs were determined as the minimal antibiotic concentration required to kill 95%. All REMA titre measurements or CFU mL^−1^ were plotted in SigmaPlot™ and 4-parameter logistic (4PL) model regressions conducted. The relative MBC_95_ of each curve was calculated and averaged. Testing was performed in duplicate with two independent biological repeats. Known antitubercular agents used in this test were RIF, INH, LZD.

#### Cytotoxicity

Compounds were tested in octuplet against two independent biological repeats. RAW264.7 cell lines (ATCC TIB-71™) and human lung epithelial cell line derived from a lung adenocarcinoma A549 (ATCC CCL-185™) were purchased from American Type Culture Collection Cells and cultured in Dulbecco's Modified Eagle's Medium – high glucose (DMEM-HG). Growth media was replaced every 2–3 days and the cells washed with Hanks' balanced salt solution (HBSS) after the removal of old media and before the addition of fresh media. Once the cell culture was grown to confluence, cells were lifted with Accutase (RAW264.7) or trypsin/EDTA (A549) and harvested for subculturing. Cells were cultured in the correct growth medium supplemented with 10% v/v foetal bovine serum (FBS), 2 mM l-glutamine and 1% penicillin/streptomycin, at 37 °C in a humidified incubator containing 5% CO_2_. Cells were passaged after reaching 90% confluence. Cells in logarithmic growth were plated in 96-well plates at a density of 1 × 10^4^ cells per well in 200 μL of media and incubated for 24 h. The media was removed and replaced with 180 μL of fresh media. To each respective well, 20 μL of the titrated test compound was added, resulting in a final drug titration of 200–1.56 μM. In all wells the final concentration of DMSO did not exceed 0.1%. Following incubation for a further 96 h at 37 °C in a humidified incubator containing 5% CO_2_, all media was removed and replaced with 100 μL of fresh media. To each well, 20 μL of 3-(4,5-dimethylthiazol-2-yl)-5-(3-carboxymethoxyphenyl)-2-(4-sulfo-phenyl)-2*H*-tetrazolium, inner salt (MTS) assay solution (Promega, Madison, WI, USA) was added and incubated in the dark at 37 °C for 4 h. The absorbance of each well was recorded at 490 nm using a microplate reader, FLUOstar Optima, BMG Labtech. Measurements were plotted in SigmaPlot™ and 4-parameter logistic (4PL) model regressions conducted. The relative IC_50_ of each compound was determined. The selectivity index (SI) was determined for the most potent compounds. (SI = IC_50_ of mammalian cell line/MIC_95_ on *Mtb*).

### Computational studies

Molecular docking was performed using molecular operating environment (MOE 2024.0601) software^40^ to generate protein–ligand complexes, which were then subject to molecular dynamics simulations using the Desmond programme of Schrödinger,^41^ the full methods are provided in the SI.

## Author contributions

LAA, JMA and DPdS performed the chemistry supervised by CS. LAA performed the computational studies supervised by CS. AK performed the CYP121A1 UV-vis binding studies supervised by DFE. SW performed the SPOTi assay. AKB performed the REMA and MBC assays while AYGA performed the MTS assay supervised by AKB. CS, LAA and AKB wrote the manuscript, and all authors revised and approved the final manuscript.

## Conflicts of interest

There are no conflicts to declare.

## Supplementary Material

MD-017-D5MD00738K-s001

## Data Availability

The data supporting this article is included in the main text or have been included as part of the supplementary information (SI). Supplementary information: Fig. S1, protein–ligand schematic for compounds 5m, 5o, 8h and 8j; Fig. S2. Protein ligand RMSD of *Mtb* CYP121A1 and (A) 5m (B) 5o (C) 8h (D) 8j over 200 ns molecular dynamics simulation; Table S1, yields and mp of final products; Table S2, MIC in μg mL^−1^; Table S3, *Mtb* strains used in study; experimental methods; NMR spectra for final compounds. See DOI: https://doi.org/10.1039/d5md00738k.
