# Supplementary material for: 3-(Pyridine-3-ylmethylene)chroman-4-one and tetralone derivatives: synthesis, Mycobacterium tuberculosis CYP121A1 enzyme inhibition and antimycobacterial activity vs drug-sensitive and drug-resistant strains
Source: RSC Med Chem. 2026 Feb 4;17(3):1672–86. doi: 10.1039/d5md00738k (PMC12895674; doi:10.1039/d5md00738k)
Supplement: MD-017-D5MD00738K-s001 [file MD-017-D5MD00738K-s001.pdf]

Supplementary Information (SI) for RSC Medicinal Chemistry.
This journal is © The Royal Society of Chemistry 2026

Supporting information

3-(Pyridine-3-ylmethylene)chroman-4-one and tetralone derivatives: synthesis, *Mycobacterium tuberculosis* CYP121A1 enzyme inhibition and antimycobacterial activity *vs* drug-sensitive and drug-resistant strains

Lama A. Alshabani, Jabril M. Abdali, Alistair K. Brown, Damião Pergentino de Sousa, Sam Wilcocks, Amit Kumar, Ahmed Y.G. Alhejaili, D. Fernando Estrada, Claire Simons

**Figure S1**. A schematic of detailed ligand atom interactions with the protein residues (A) **5m** (B) **5o** (C) **8h** (D) **8j** over 200 ns molecular dynamics simulation	S2
**Figure S2**. Protein ligand RMSD of *Mtb* CYP121A1 and (A) **5m** (B) **5o** (C) **8h** (D) **8j** over 200 ns molecular dynamics simulation.	S3
**Table S1:** Yield and m.p. of final chroman-4-one (**5**) and tetralone (**8**) pyridine derivatives	S4
**Table S2**. MIC (μg/mL) of chroman-4-ones (**5**) and tetralones (**8**) against *Mtb* wild-type and resistant strains.	S5
**Table S3:** Different *Mtb* strains used in the study	S6
Experimental methods	S7-S25
NMR Spectra for final compounds	S26-S55
References	S56-S57

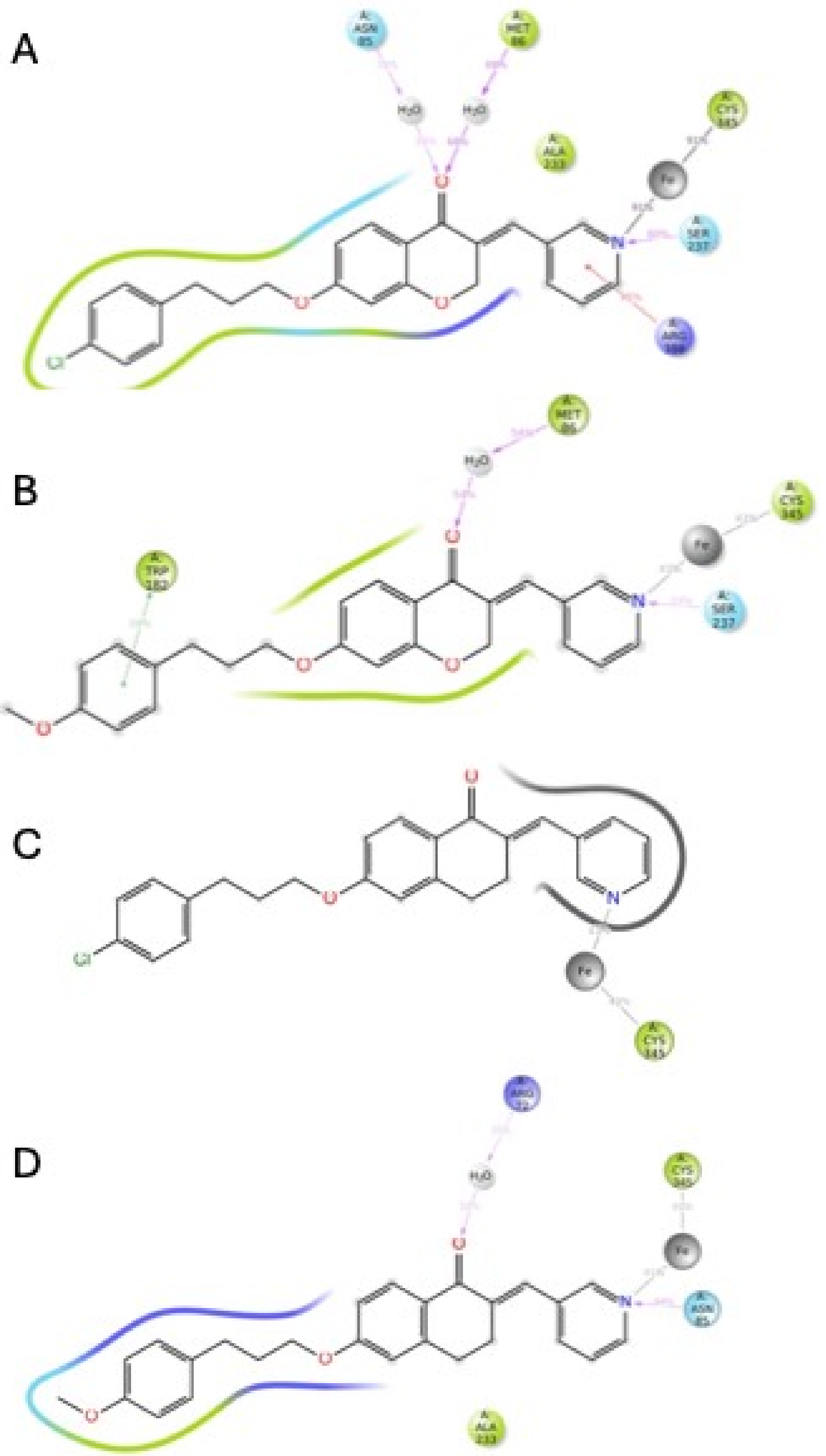

Figure S1. A schematic of detailed ligand atom interactions with the protein residues (A) **5m** (B) **5o** (C) **8h** (D) **8j** over 200 ns molecular dynamics simulation. Interactions that occur more than 30% of the simulation time in the selected trajectory (0 through 200 ns) are shown.

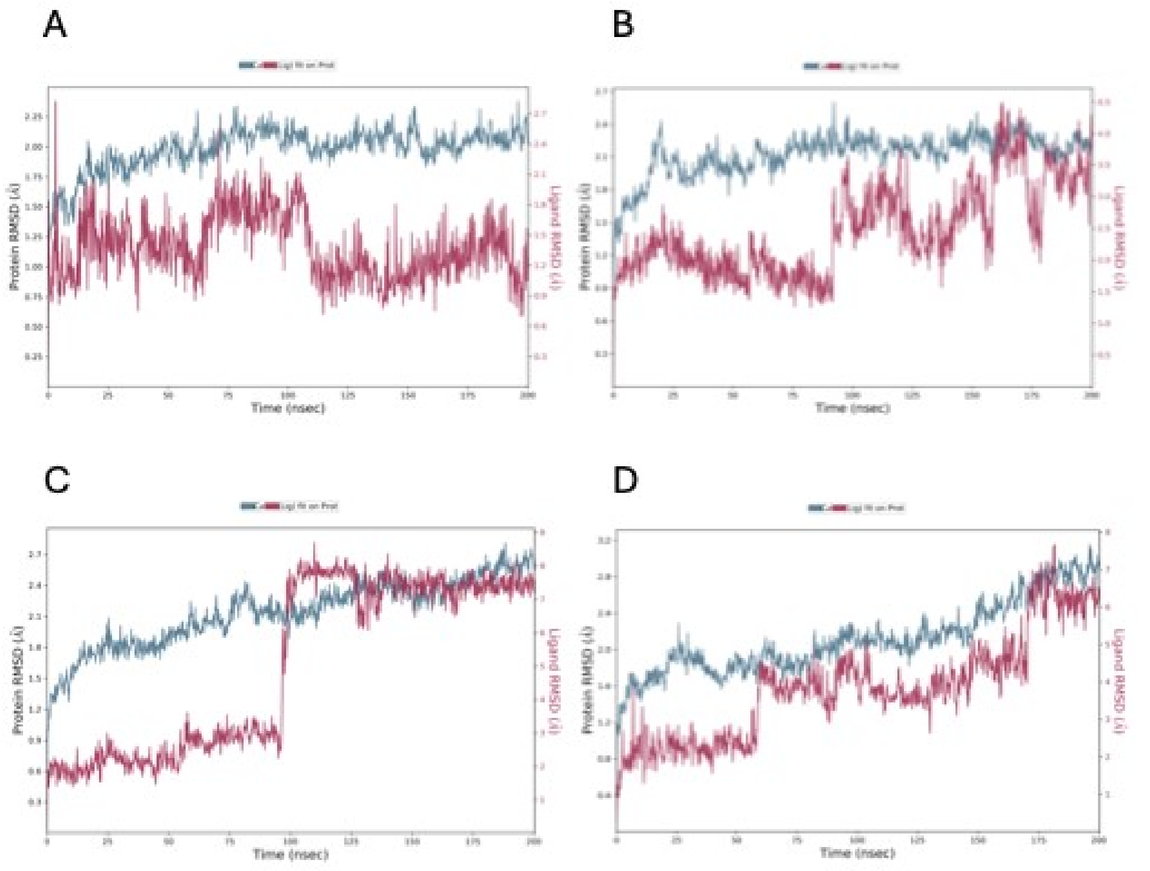

Figure S2. Protein ligand RMSD of *Mtb* CYP121A1 and (A) **5m** (B) **5o** (C) **8h** (D) **8j** over 200 ns molecular dynamics simulation.

Table S1. Yield and m.p. of chroman-4-one (**5**) and tetralone (**8**) pyridine derivatives

Cmpd	Yield (%)	m.p. (°C)	Cmpd	Yield (%)	m.p. (°C)
**5a**	65	152-154	**5n**	35	120-122
**5b**	39	118-120	**5o**	58	115-117
**5c**	63	94-96	**8a**	42	90-92
**5d**	48	152-154	**8b**	46	95-97
**5e**	54	152-154	**8c**	39	86-66
**5f**	40	130-132	**8d**	26	90-92
**5g**	50	140-142	**8e**	31	Oil
**5h**	32	117-119	**8f**	32	113-115
**5i**	46	100-102	**8g**	38	88-90
**5j**	47	110-112	**8h**	34	97-99
**5k**	74	120-122	**8i**	47	102-104
**5l**	87	126-128	**8j**	34	88-90
**5m**	39	129-131			

*complex mixture with challenging purification

Table S2. MIC (µg/mL) of final pyridine chromanones (**5**) and tetralone derivatives (**8**) against *Mtb* wild-type and resistant strains.

	**MIC[a] (µg/mL)**					
**Cmpd**	*H37Rv* (wild type)	$mc^2$7902 (susceptible)	$mc^2$8247 (RIF^R)	$mc^2$8245 (INH^R)	$mc^2$8250 (RIF^R & INH^R)	$mc^2$8256 (RIF^R & INH^R)
**5a**	ND	ND	ND	ND	ND	ND
**5b**	75	32	32	32	32	32
**5c**	8.1	4	4	4	4	4
**5d**	0.5	4	2	4	4	2
**5e**	4	2	1	2	2	1
**5f**	4.5	4	4	4	4	4
**5g**	9.4	8	4	4	4	4
**5h**	8.1	4	2	2	2	2
**5i**	10.9	4	2	2	2	4
**5j**	2	8	2	2	2	2
**5k**	9.3	8	2	4	4	4
**5l**	9.7	4	2	2	2	2
**5m**	20.3	4	2	2	2	2
**5n**	22.5	8	2	4	4	4
**5o**	10.0	2	1	2	2	2
**8a**	8.9	8	2	2	1	2
**8b**	1.1	8	2	2	4	2
**8c**	9.7	4	1	1	4	1
**8d**	5.4	8	1	1	2	1
**8e**	9.6	8	2	4	1	4
**8f**	1.1	8	1	1	2	1
**8g**	1.2	8	1	2	2	2
**8h**	1.2	8	1	2	2	1
**8i**	11.2	4	1	1	1	1
**8j**	1.2	8	4	4	4	4
INH	0.2	0.1	0.1	-	-	-
RIF	0.2	0.05	-	0.05	-	-
EMB	-	0.5	1	1	1	0.5
ETH	-	1.0	1	1	1	1
KAN	7.8	-	-	-	-	-

[a] Results are the average of two independent experiments. ND: not detected. INH^R – isoniazid resistant, RIF^R – rifampicin resistant. INH – isoniazid, RIF – rifampicin, EMB – ethambutol, ETH – ethionamide, KAN – kanamycin

Table S3: Different *Mtb* strains used in the study[1]

**Strains**	**Description**
H37Rv (**wild- type**)	Pantothenate and Leucine auxotrophic (Δ*panCD* Δ*leuCD*)
Mtb mc^{2}7902 (**Drug- susceptible**)	Pantothenate, Leucine and arginine triple auxotroph (Δ*leuCD* Δ*panCD* Δ*argB*)
Mtb mc^{2}8245 (**INH^R**)	**Monoresistance:** Large genomic deletion contains *katG* and 50 other genes (*Rv1867* to *Rv1916* genome deletion)
Mtb mc^{2}8247 (**RIF^R**)	**Monoresistance:** *rpoB* mutation (H445Y)
Mtb mc^{2}8250 (**RIF^R & INH^R**)	**MDR mutant:** *rpoB* mutation (H445Y) and genome deletion (*Rv1872c* to *Rv1918c*)
Mtb mc^{2}8256 (**RIF^R & INH^R**)	**MDR mutant:** *rpoB* mutation (H445Y) and *katG* mutation (Δ305–312 bp, frameshift/stop codon)

Experimental Methods for Synthesis of Intermediates

3-Chloro-1-(2,4-dihydroxyphenyl)propan-1-one (2).[2]

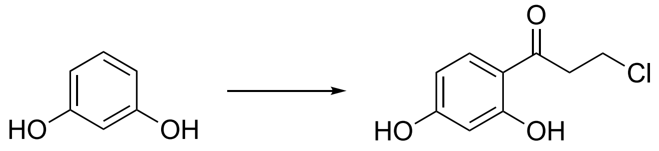

1 2

To a stirred mixture of resorcinol (**1**) (1.1 g, 10.0 mmol) and 3-chloropropionic acid (1.1 g, 10.1 mmol) was added trifluoromethane sulfonic acid (CF_3SO_3H) (5.4 g, 36.0 mmol) in one portion. The solution was warmed to 85 °C for 1.5 h. The reaction was cooled to room temperature and diluted with H_2O (50 mL) and extracted with $CHCl_3$ (8 x 50 mL). The combined organic layers were dried (Mg_2SO_4), then concentrated under reduced pressure to give the product as a yellow solid. Yield: 1.24 g (62%), m.p. 88 – 90 °C, TLC (petroleum ether - EtOAc 1:1 v/v) R_f 0.77. ^{1}H NMR (DMSO-d_6): δ 12.28 (s, 1H, OH), 10.67 (s, 1H, OH), 7.80 (d, J = 9.0 Hz, 1H, Ar), 6.40 (dd, J = 2.5, 9.0 Hz, 1H, Ar), 6.28 (d, J = 2.0 Hz, 1H, Ar), 3.92 (t, J = 6.0 Hz, 2H, CH_2), 3.48 (t, J = 6.0 Hz, 2H, CH_2).

7-Hydroxychroman-4-one (3)[3]

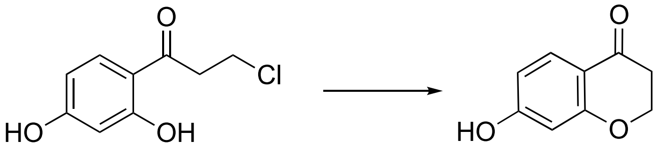

2 3

2M aq. NaOH (50 mL) was added to (**2**) (0.8 g, 4.0 mmol) in an ice-bath (0 °C) and stirred at 0 °C for 3 h. On completion, the reaction was acidified to pH 2 with c.HCl and the resulting precipitate collected by vacuum filtration. The filtrate was washed with EtOAc (3 x 50 mL), and the combined organic layers were dried ($MgSO_4$) and evaporated under reduced pressure to give the crude product. The combined solids from filtration and extraction of the filtrate were purified by recrystallisation (toluene) to give the pure product as a beige solid. Yield: 1.51

g (92%), m.p. 150-152 °C (lit. m.p. 148 – 150 °C)[2], TLC (petroleum ether - EtOAc 1:1 v/v) R_f 0.52. ^{1}H NMR (DMSO-d_6): δ 10.54 (s, 1H, OH), 7.63 (d, J = 8.5 Hz, 1H, Ar), 6.49 (dd, J = 2.5, 9.0 Hz, 1H, Ar), 6.31 (d, J = 2.0 Hz, 1H, Ar), 4.46 (t, J = 6.5 Hz, 2H, CH_2), 2.68 (t, J = 6.0 Hz, 2H, CH_2).

General method for the preparation of alkylated products (4 and 7)

To a solution of 7-hydroxychroman-4-one (**3**) or 7-hydroxytetralone (**6**) (1 equiv.) and K_2CO_3 (1 equiv.) in dry DMF (0.5 mL/mmol) was added alkyl halide (1.5 equiv.) and the mixture stirred at 80 °C for 3 h. On completion, the reaction was diluted with water (20 mL/10 mmol) and extracted with EtOAc (2 x 40 mL). The combined organic layers were dried ($MgSO_4$) then evaporated under reduced pressure to give the crude product, which was purified by gradient column chromatography (petroleum ether – EtOAc).

7-Ethoxychroman-4-one (4b)[4]

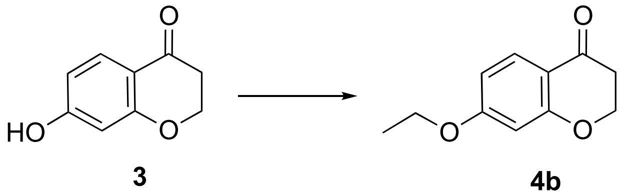

Prepared from 7-hydroxychroman-4-one (**3**) (1.8 g, 11.0 mmol) and iodoethane (2.57 g, 16.5 mmol). The pure compound was eluted with petroleum ether – EtOAc 70:30 v/v as a white solid. Yield: 1.81 g (86%), m.p. 44-46 °C (lit. m.p. 148 – 150 °C)[4], TLC (petroleum ether - EtOAc 1:1 v/v) R_f 0.69. ^{1}H NMR (DMSO-d_6): δ 7.85 (d, J = 8.5 Hz, 1H, Ar), 6.57 (dd, J = 2.0, 8.5 Hz, 1H, Ar), 6.40 (d, J = 2.0 Hz, 1H, Ar), 4.52 (t, J = 6.5 Hz, 2H, CH_2), 4.07 (q, J = 7.0 Hz, 2H, CH_2CH$_3$), 2.76 (t, J = 6.5 Hz, 2H, CH_2), 1.43 (t, J = 7.0 Hz, 3H, CH$_2$$CH_3$).

7-(Pentyloxy)chroman-4-one (4c)

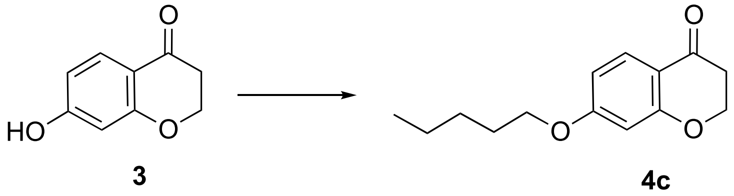

Prepared from 7-hydroxychroman-4-one (**3**) (1.8 g, 11.0 mmol) and 1-bromopentane (2.5 g, 16.5 mmol). The pure compound was eluted with petroleum ether – EtOAc 75:25 v/v as a yellow oil. Yield: 2.16 g (84%), TLC (petroleum ether - EtOAc 2:1 v/v) R_f 0.62. ^{1}H NMR (DMSO-d_6): δ 7.85 (d, J = 9.0 Hz, 1H, Ar), 6.59 (dd, J = 2.5, 9.0 Hz, 1H, Ar), 6.41 (d, J = 2.5 Hz, 1H, Ar), 4.52 (t, J = 6.5 Hz, 2H, CH_2), 3.99 (t, J = 6.5 Hz, 2H, CH_2), 2.76 (t, J = 6.5 Hz, 2H, CH_2), 1.81 (quintet, J = 6.5 Hz, 2H, CH_2), 1.38 – 1.48 (m, 4H, 2 x CH_2), 0.95 (t, J = 7.0 Hz, 3H, CH_3). ^{13}C NMR (DMSO-d_6): δ 190.54 (C=O), 165.62 (C, Ar), 163.81 (C, Ar), 128.84 (CH, Ar), 115.10 (C, Ar), 110.30 (CH, Ar), 101.19 (CH, Ar), 68.45 (CH_2), 67.37 (CH_2), 37.45 (CH_2), 28.68 (CH_2), 28.09 (CH_2), 22.40 (CH_2), 13.99 (CH_3). HPLC: 99%, RT= 4.87 min, HRMS (ESI, m/z): theoretical mass: 257.1154 $[M+Na]^+$, observed mass: 257.1147 $[M+Na]^+$

7-(Benzyloxy)chroman-4-one (4d)[4]

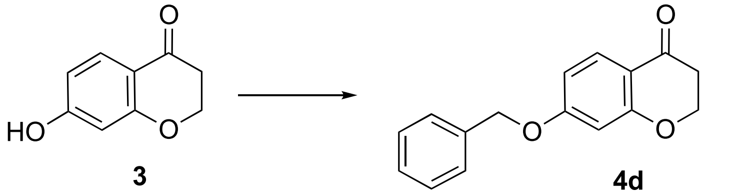

Prepared from 7-hydroxychroman-4-one (**3**) (1.8 g, 11.0 mmol) and benzyl bromide (2.8 g, 16.5 mmol). The pure compound was eluted with petroleum ether – EtOAc 75:25 v/v as a yellow solid. Yield: 1.51 g (57%), m.p: 98 – 100 °C (lit. m.p. 103 – 104 °C)[4], TLC (petroleum ether - EtOAc 2:1 v/v) R_f 0.5. ^{1}H NMR ($CDCl_3$): δ 7.87 (d, J = 9.0 Hz, 1H, Ar), 7.45 (m, 5H, Ar), 6.68 (dd, J = 2.5, 7.5 Hz, 1H, Ar), 6.51 (d, J =2.5 Hz, 1H, Ar), 5.12 (s, 2H, CH_2), 4.53 (t, J = 6.5 Hz, 2H, CH_2), 2.78 (t, J = 6.5 Hz, 2H, CH_2).

7-((4-Fluorobenzyl)oxy)chroman-4-one (4e)[5]

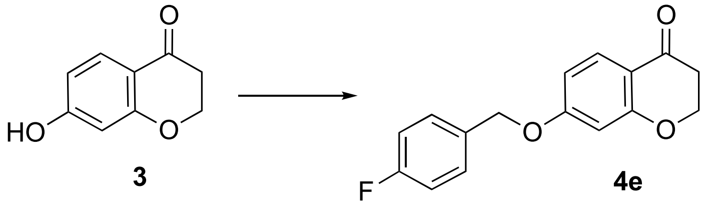

Prepared from 7-hydroxychroman-4-one (**3**) (1.8 g, 11.0 mmol) and 4-fluorobenzyl chloride (2.4 g, 16.5 mmol). The pure compound was eluted with petroleum ether – EtOAc 70:30 v/v as a brown solid. Yield: 1.36 g (48%), m.p: 82-84 °C (lit. m.p. 125 – 127 °C)[5], TLC (petroleum ether - EtOAc 1:1 v/v) R_f 0.48. ^{1}H NMR ($CDCl_3$): δ 7.87 (d, J = 9.5 Hz, 1H, Ar), 7.43 (m, 2H, Ar), 7.13 (m, 2H, Ar), 6.67 (dd, J = 2.5, 8.8 Hz, 1H, Ar), 6.49 (d, J = 2.5 Hz, 1H, Ar), 5.07 (s, 2H, CH_2), 4.53 (t, J = 6.5 Hz, 2H, CH_2), 2.78 (t, J = 6.5 Hz, 2H, CH_2). ^{19}F NMR ($CDCl_3$): δ -113.23 (Ar-F).

7-Phenethoxychroman-4-one (4f)[6]

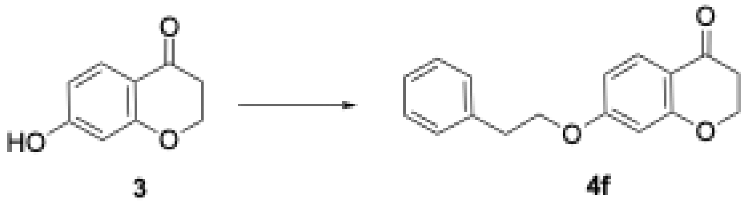

Prepared from 7-hydroxychroman-4-one (**3**) (0.5 g, 3.05 mmol) and phenethyl bromide (0.85 g, 4.57 mmol). The pure compound was eluted with petroleum ether – EtOAc 70:30 v/v to give a white solid. Yield: 0.41 g (57%), m.p: 87-88 °C, TLC (petroleum ether - EtOAc 2:1 v/v) R_f 0.62. ^{1}H NMR ($CDCl_3$): δ 7.84 (d, J = 8.8 Hz, 1H, Ar), 7.31 (m, 5H, Ar), 6.59 (dd, J = 2.0, 8.8 Hz, 1H, Ar), 6.42 (d, J = 2.0 Hz, 1H, Ar), 4.52 (t, J = 6.4 Hz, 2H, CH_2), 4.22 (t, J = 7.0 Hz, 2H, CH_2), 3.13 (t, J = 7.0 Hz, 2H, CH_2), 2.76 (t, J = 6.4 Hz, 2H, CH_2).

7-(4-Fluorophenethoxy)chroman-4-one (4g)

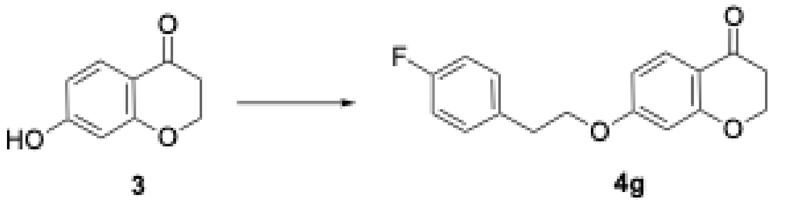

Prepared from 7-hydroxychroman-4-one (**3**) (0.5 g, 4.57 mmol) and 4-fluorophenethyl bromide (0.93 g, 4.57 mmol). The pure compound was eluted with petroleum ether – EtOAc 70:30 v/v to give a white solid. Yield: 0.43 g (53%), m.p: 114-115 °C, TLC (petroleum ether - EtOAc 2:1 v/v) R_f 0.77. ^{1}H NMR ($CDCl_3$): δ 7.85 (d, J = 8.8 Hz, 1H, Ar), 7.28-7.01 (m, 4H, Ar), 6.58 (d, J = 8.8 Hz, 1H, Ar), 6.41 (s, 1H, Ar), 4.52 (t, J = 6.4 Hz, 2H, CH_2), 4.10 (t, J = 6.7 Hz, 2H, CH_2), 3.09 (t, J = 6.7 Hz, 2H, CH_2), 2.76 (t, J = 6.4 Hz, 2H, CH_2). ^{13}C NMR ($CDCl_3$): δ 190.44 (C=O), 165.05 (C, Ar), 163.75 (C, Ar), 161.76 (d, $^1J_{CF}$ = 245 Hz, C-F, Ar), 133.43 (d, $^4J_{CF}$ = 3.6 Hz, C, Ar), 130.38 (d, $^3J_{CF}$ = 8.15 Hz, 2 x CH, Ar), 128.92 (CH, Ar), 115.37 (C, Ar), 115.34 (d, $^2J_{CF}$ = 21 Hz, 2 x CH, Ar), 110.14 (CH, Ar), 101.35 (CH, Ar), 68.88 (CH_2), 67.36 (CH_2), 37.43 (CH_2), 34.68 (CH_2). ^{19}F NMR: δ -116.39 (Ar-F).

7-(4-Chlorophenethoxy)chroman-4-one (4h)

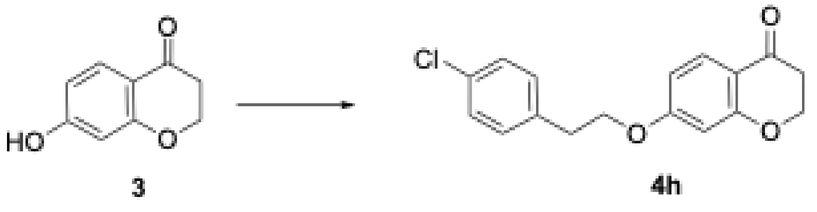

Prepared from 7-hydroxychroman-4-one (**3**) (1.8 g, 11.0 mmol) and 4-chlorophenethyl bromide (3.6 g, 16.5 mmol). The pure compound was eluted with petroleum ether – EtOAc 80:20 v/v to give a beige solid. Yield: 1.56 g (47%), m.p: 90-92 °C, TLC (petroleum ether - EtOAc 1:1 v/v) R_f 0.7. ^{1}H NMR ($CDCl_3$): δ 7.84 (d, J = 9.0 Hz, 1H, Ar), 7.32 (d, J = 8.0 Hz, 2H, Ar), 7.23 (d, J = 8.5 Hz, 2H, Ar), 6.58 (dd, J = 2.5, 9.0 Hz, 1H, Ar), 6.40 (d, J = 2.5 Hz,

1H, Ar), 4.52 (t, J = 7.5 Hz, 2H, CH_2), 4.19 (t, J = 7.5 Hz, 2H, CH_2), 3.09 (t, J = 8.0 Hz, 2H, CH_2), 2.76 (t, J = 6.5 Hz, 2H, CH_2). ^{13}C NMR ($CDCl_3$): δ 190.10 (C=O), 165.01 (C, Ar), 163.68 (C, Ar), 136.31 (C, Ar), 132.55 (C, Ar), 130.28 (2 x CH, Ar), 128.88 (CH, Ar), 128.69 (2 x CH, Ar), 115.37 (C, Ar), 110.15 (CH, Ar), 101.35 (CH, Ar), 68.64 (CH_2), 67.38 (CH_2), 37.44 (CH_2), 34.84 (CH_2). Anal. Calcd: C 67.44%, H 4.99%. Found: C 67.54 %, H 5.12 %.

7-(4-Bromophenethoxy)chroman-4-one (4i)

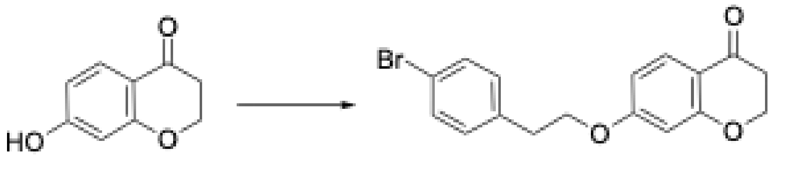

Prepared from 7-hydroxychroman-4-one (**3**) (1.3 g, 7.92 mmol) and 4-bromophenethyl bromide (3.13 g, 11.88 mmol). The pure compound was eluted with petroleum ether – EtOAc 70:30 v/v to give a white solid. Yield: 0.83 g (30%), m.p: 83-85 °C, TLC (petroleum ether - EtOAc 2:1 v/v) R_f 0.50. ^1H NMR ($CDCl_3$): δ 8.02 (d, J = 8.2 Hz, 1H, Ar), 7.64 (d, J = 8.5 Hz, 2H, Ar), 7.35 (d, J = 8.5 Hz, 2H, Ar), 6.75 (dd, J = 8.8, 2.4 Hz, 1H, Ar), 6.57 (d, J = 2.4 Hz, 1H, Ar), 4.70 (t, J = 6.4 Hz, 2H, CH_2), 4.37 (t, J = 6.7 Hz, 2H, CH_2), 3.25 (t, J = 6.7 Hz, 2H, CH_2), 2.94 (t, J = 6.4 Hz, 2H, CH_2). ^{13}C NMR ($CDCl_3$): δ 190.74 (C=O), 164.97 (C, Ar), 163.74 (C, Ar), 136.79 (C, Ar), 131.63 (2 x CH, Ar), 130.70 (2 x CH, Ar), 128.94 (CH, Ar), 120.59 (C, Ar), 115.39 (C, Ar), 110.13 (CH, Ar), 101.33 (CH, Ar), 68.53 (CH_2), 67.37 (CH_2), 37.42 (CH_2), 34.89 (CH_2). Anal. Calcd: C 58.81%, H 4.35%. Found: C 58.78 %, H 4.24 %.

7-(4-Methoxyphenethoxy)chroman-4-one (4j)

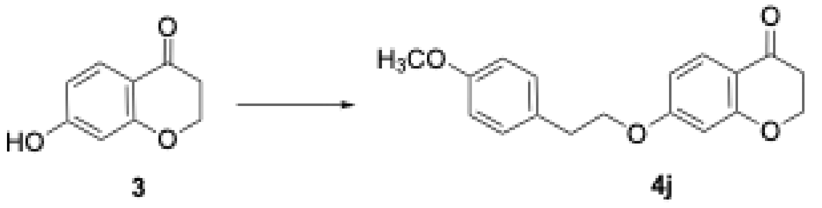

Prepared from 7-hydroxychroman-4-one (**3**) (1.8 g, 11.0 mmol) and 4-methoxyphenethyl bromide (3.5 g, 16.5 mmol). The pure compound was eluted with petroleum ether – EtOAc 80:20 v/v to give a white solid. Yield: 2.3 g (71%), m.p: 88-90 °C, TLC (petroleum ether - EtOAc 1:1 v/v) R_f 0.67. ^{1}H NMR ($CDCl_3$): δ 7.84 (d, J = 9.0 Hz, 1H, Ar), 7.21 (d, J = 9.0 Hz, 2H, Ar), 6.88 (d, J = 8.5 Hz, 2H, Ar), 6.59 (dd, J = 2.5, 9.0 Hz, 1H, Ar), 6.41 (d, J = 2.5 Hz, 1H, Ar), 4.52 (t, J = 6.5 Hz, 2H, CH_2), 4.17 (t, J = 7.0 Hz, 2H, CH_2), 3.82 (s, 3H, CH_3), 3.06 (t, J = 7.0 Hz, 2H, CH_2), 2.76 (t, J = 6.5 Hz, 2H, CH_2). ^{13}C NMR ($CDCl_3$): δ 190.13 (C=O), 165.29 (C, Ar), 163.79 (C, Ar), 158.38 (C, Ar), 129.91 (2 x CH, Ar), 129.76 (C, Ar), 128.97 (CH, Ar), 115.22 (C, Ar), 114.01 (2 x CH, Ar), 110.25 (CH, Ar), 101.35 (CH, Ar), 69.26 (CH_2), 67.37 (CH_2), 55.22 (CH_3), 37.40 (CH_2), 34.52 (CH_2). Anal. Calcd: C 72.47%, H 6.08%. Found: C 72.26%, H 6.02%.

7-(3-Phenylpropoxy)chroman-4-one (4k)[6]

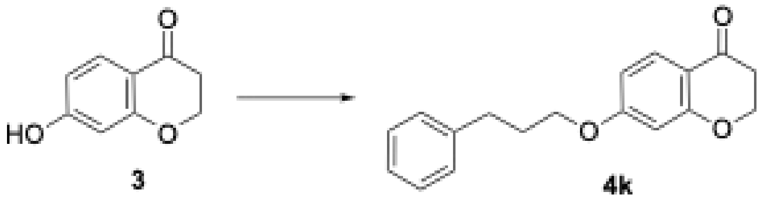

Prepared from 7-hydroxychroman-4-one (**3**) (1.0 g, 6.09 mmol) and 4-bromophenylpropyl bromide (1.82 g, 9.14 mmol). The pure compound was eluted with petroleum ether – EtOAc 70:30 v/v to give a white solid. Yield: 0.49 g (87%), m.p: 68-70 °C, TLC (petroleum ether - EtOAc 2:1 v/v) R_f 0.60. ^{1}H NMR ($CDCl_3$): δ 7.86 (d, J = 8.8 Hz, 1H, Ar), 7.33-7.21 (m, 5H, Ar), 6.59 (d, J = 8.8 Hz, 1H, Ar), 6.4 (s, 1H, Ar), 4.53 (t, J = 6.2 Hz, 2H, H-9′), 4.00 (t, J = 6.2

Hz, 2H, CH_2), 2.82 (t, J = 7.5 Hz, 2H, CH_2), 2.77 (t, J = 6.2 Hz, 2H, CH_2), 2.15 (quintet, J = 6.7 Hz, 2H, CH_2).

7-(3-(4-Fluorophenyl)propoxy)chroman-4-one (4l)

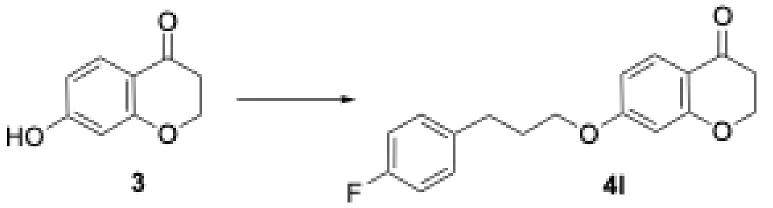

Prepared from 7-hydroxychroman-4-one (**3**) (0.5 g, 3.24 mmol) and 1-(3-bromopropyl)-4-fluorobenzene (0.53 g, 3.24 mmol). The pure compound was eluted with petroleum ether – EtOAc 70:30 v/v to give a white solid. Yield: 0.70 g (70%), m.p: 64-66 °C, TLC (petroleum ether - EtOAc 2:1 v/v) R_f 0.60. ^{1}H NMR ($CDCl_3$): δ 7.85 (d, J = 8.8 Hz, 1H, Ar), 7.17 (m, 2H, Ar), 7.00 (m, 2H, Ar), 6.59 (dd, J = 2.4, 8.8 Hz, 1H, Ar), 6.39 (d, J = 2.4 Hz, 1H, H-8), 4.53 (t, J = 5.5 Hz, 2H, CH_2), 3.98 (t, J = 6.2 Hz, 2H, CH_2), 2.82 (m, 4H, 2 x CH_2), 2.11 (m, 2H, CH_2). ^{13}C NMR ($CDCl_3$): δ 190.48 (C=O), 165.34 (C, Ar), 163.77 (C, Ar), 161.37 (d, $^1J_{CF}$ = 243.6 Hz, C-F, Ar), 136.67 (d, $^4J_{CF}$ = 3.5 Hz, C, Ar), 129.82 (d, $^3J_{CF}$ = 7.5 Hz, 2 x CH, Ar), 128.89 (CH, Ar), 115.26 (C, Ar), 115.22 (d, $^2J_{CF}$ = 22.2 Hz, 2 x CH, Ar), 110.21 (CH, Ar), 101.21 (CH, Ar), 67.37 (CH_2), 67.05 (CH_2), 37.43 (CH_2), 31.19 (CH_2), 30.60 (CH_2). ^{19}F NMR: δ -117.34 (Ar-F). Anal. Calcd: C 71.99%, H 5.71%. Found: C 71.67 %, H 5.77 %.

7-(3-(4-Chlorophenyl)propoxy)chroman-4-one (4m)

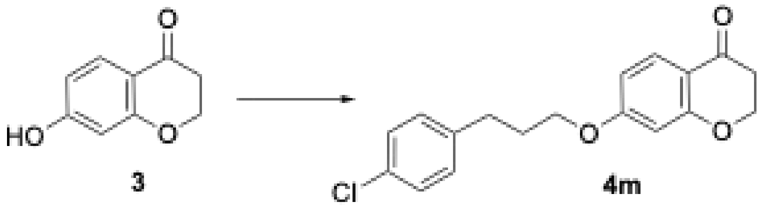

Prepared from 7-hydroxychroman-4-one (**3**) (1.0 g, 6.09 mmol) and 1-(3-bromopropyl)-4-chlorobenzene (1.9 g, 8.14 mmol). The pure compound was eluted with petroleum ether – EtOAc 70:30 v/v to give a biege solid. Yield: 1.7 g (90%), m.p: 99-100 °C, TLC (petroleum

ether - EtOAc 2:1 v/v) R_f 0.49. ^{1}H NMR ($CDCl_3$): δ 7.86 (d, J = 8.8 Hz, 1H, Ar), 7.27 (d, J = 8.8 Hz, 2H, Ar), 7.15 (d, J = 7.5 Hz, 2H, Ar), 6.59 (d, J = 8.8 Hz, 1H, Ar), 6.39 (s, 1H, Ar), 4.53 (t, J = 5.5 Hz, 2H, CH_2), 3.98 (t, J = 5.4 Hz, 2H, CH_2), 2.79 (t, J = 7.6 Hz, 2H, CH_2), 2.77 (t, J = 6.3 Hz, 2H, CH_2), 2.11 (quintet, J = 6.6 Hz, 2H, CH_2). ^{13}C NMR ($CDCl_3$): δ 190.49 (C=O), 165.30 (C, Ar), 163.77 (C, Ar), 139.51 (C, Ar), 131.82 (C, Ar), 129.89 (2 x CH, Ar), 128.91 (CH, Ar), 128.58 (2 x CH, Ar), 115.28 (C, Ar), 110.20 (CH, Ar), 101.22 (CH, Ar), 67.38 (CH_2), 67.00 (CH_2), 37.43 (CH_2), 31.37 (CH_2), 30.40 (CH_2). Anal. Calcd: C 68.25%, H 5.41%. Found: C 68.19 %, H 5.22 %.

7-(3-(4-Bromophenyl)propoxy)chroman-4-one (4n)

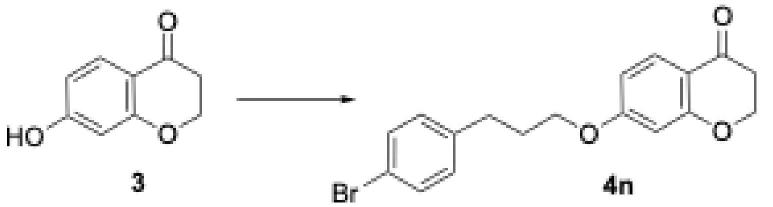

Prepared from 7-hydroxychroman-4-one (**3**) (0.59 g, 3.6 mmol) and 1-(3-bromopropyl)-4-bromobenzene (1.5 g, 5.4 mmol). The pure compound was eluted with petroleum ether – EtOAc 70:30 v/v to give a white solid. Yield: 1.05 g (80%), m.p: 110-112 °C, TLC (petroleum ether - EtOAc 2:1 v/v) R_f 0.55. ^{1}H NMR ($CDCl_3$): δ 7.82 (d, J = 8.8 Hz, 1H, Ar), 7.42 (d, J = 8.4 Hz, 2H, Ar), 7.09 (d, J = 8.5 Hz, 2H, Ar), 6.59 (dd, J = 2.4, 8.8 Hz, 1H, Ar), 6.38 (d, J = 2.4 Hz, 1H, Ar), 4.53 (t, J = 6.4 Hz, 2H, CH_2), 3.98 (t, J = 6.2 Hz, 2H, CH_2), 2.79 (m, 4H, 2 x CH_2), 2.11 (m, 2H, CH_2). ^{13}C NMR ($CDCl_3$): δ 190.48 (C=O), 165.29 (C, Ar), 163.77 (C, Ar), 140.03 (C, Ar), 131.54 (C, Ar), 130.25 (2 x CH, Ar), 128.90 (CH, Ar), 119.83 (2 x CH, Ar), 115.28 (C, Ar), 110.19 (CH, Ar), 101.22 (CH, Ar), 67.37 (CH_2), 66.98 (CH_2), 37.43 (CH_2), 31.43 (CH_2), 30.34 (CH_2). Anal. Calcd: C 59.85%, H 4.74%. Found: C 59.83 %, H 4.75 %.

7-(3-(4-Methoxyphenyl)propoxy)chroman-4-one (4o)

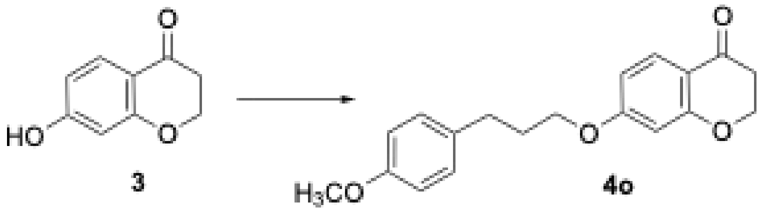

Prepared from 7-hydroxychroman-4-one (**3**) (1.0 g, 6.09 mmol) and 1-(3-bromopropyl)-4-methoxybenzene (2.0 g, 8.73 mmol). The pure compound was eluted with petroleum ether – EtOAc 70:30 v/v to give a brown solid. Yield: 1.57 g (83%), m.p: 69-71 °C, TLC (petroleum ether - EtOAc 2:1 v/v) R_f 0.49. ^{1}H NMR ($CDCl_3$): δ 7.85 (d, *J* = 8.8 Hz, 1H, Ar), 7.13 (d, *J* = 8.4 Hz, 2H, Ar), 6.86 (d, *J* = 8.5 Hz, 2H, Ar), 6.59 (dd, *J* = 2.2, 8.8 Hz, 1H, H-6), 6.39 (d, *J* = 2.1 Hz, H-8), 4.53 (t, *J* = 6.4 Hz, 2H, CH_2), 3.98 (t, *J* = 6.3 Hz, 2H, CH_2), 3.81 (s, 3H, OCH_3), 2.77 (m, 4H, 2 x CH_2), 2.10 (quintet, *J* = 7.0 Hz, 2H, CH_2). ^{13}C NMR ($CDCl_3$): δ 190.51 (C=O), 165.47 (C, C-O), 163.77 (C, C-O), 157.93 (C, Ar), 133.10 (C, Ar), 129.38 (2 x CH, Ar), 128.91 (CH, Ar), 115.19 (C, Ar), 113.89 (2 x CH, Ar), 110.27 (CH, Ar), 101.27 (CH, Ar), 67.35 (CH_2), 67.24 (CH_2), 55.26 (CH_3), 37.43 (CH_2), 31.04 (CH_2), 30.70 (CH_2). Anal. Calcd: C 73.06%, H 6.45%. Found: C 72.68 %, H 6.41%.

6-Phenethoxy-3,4-dihydronaphthalen-1(2*H*)-one (7a)[7]

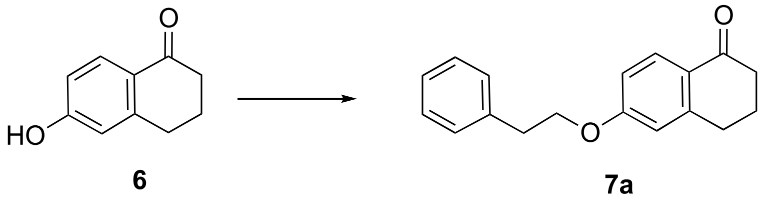

Prepared from 6-hydroxytetralone (**6**) (3.0 g, 18.5 mmol) and phenethyl bromide (3.75 mL, 27.8 mmol). The pure compound was eluted with petroleum ether – EtOAc 75:25 v/v to give a biege solid. Yield: 2.1 g (43%), m.p: 51-53 °C, TLC (petroleum ether - EtOAc 3:1 v/v) R_f 0.47. ^{1}H NMR ($CDCl_3$): δ 8.02 (d, *J* = 8.7 Hz, 1H, Ar), 7.31 (m, 5H, Ar), 6.85 (d, *J* = 8.7 Hz, 1H,

Ar), 6.72 (s, 1H, Ar), 4.25 (t, J = 7.0 Hz, 2H, OCH_2), 3.14 (t, J = 7.0 Hz, 2H, CH_2), 2.93 (t, J = 6.0 Hz, 2H, CH_2), 2.63 (t, J = 6.5 Hz, 2H, CH_2), 2.13 (quintet, J = 6.3 Hz, 2H, CH_2).

7-(4-Fluorophenethoxy)-3,4-dihydronaphthalen-1(2*H*)-one (7b)[8]

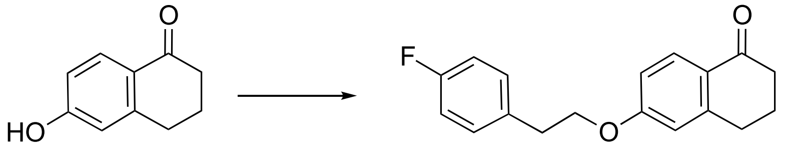

6 **7b**

Prepared from 6-hydroxytetralone (**6**) (1.1 g, 6.7 mmol) and 4-fluorophenethyl bromide (1.4 mL, 10 mmol). The pure compound was eluted with petroleum ether – EtOAc 75:25 v/v to give a light pink solid. Yield: 0.83 g (44%), m.p: 68-70 °C, TLC (petroleum ether - EtOAc 3:1 v/v) R_f 0.35. ^{1}H NMR ($CDCl_3$): δ 8.03 (d, J = 8.7 Hz, 1H, Ar), 7.26 (m, 2H, Ar), 7.03 (t, J = 8.3 Hz, 2H, Ar), 6.83 (d, J = 8.8 Hz, 1H, Ar), 6.70 (s, 1H, Ar), 4.22 (t, J = 6.7 Hz, 2H, OCH_2), 3.10 (t, J = 6.7 Hz, 2H, CH_2), 2.93 (t, J = 5.8 Hz, 2H, CH_2), 2.62 (t, J = 6.3 Hz, 2H, CH_2), 2.13 (quintet, J = 6.2 Hz, 2H, CH_2). ^{19}F NMR ($CDCl_3$): δ -116.47 (Ar-F).

6-(4-Chlorophenethoxy)-3,4-dihydronaphthalen-1(2*H*)-one (7c)

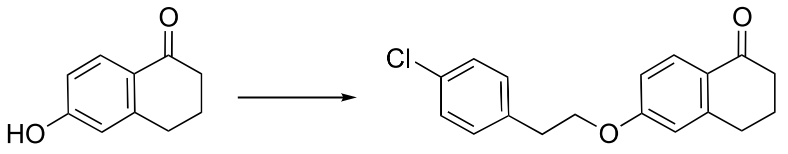

6 **7c**

Prepared from 6-hydroxytetralone (**6**) (0.44 g, 2.7 mmol) and 4-chlorophenethyl bromide (0.58 mL, 4 mmol). The pure compound was eluted with petroleum ether – EtOAc 80:20 v/v to give a biege solid. Yield: 0.32 g (40%), m.p: 69-71 °C, TLC (petroleum ether - EtOAc 3:1 v/v) R_f 0.30. ^{1}H NMR ($CDCl_3$): δ 8.02 (d, J = 8.5 Hz, 1H, Ar), 7.31 (d, J = 7.5 Hz, 2H, Ar), 7.24 (t, J = 8.0 Hz, 2H, Ar), 6.83 (d, J = 8.5 Hz, 1H, Ar), 6.70 (s, 1H, Ar), 4.22 (t, J = 6.5 Hz, 2H, CH_2), 3.10 (t, J = 6.5 Hz, 2H, CH_2), 2.93 (t, J = 5.5 Hz, 2H, CH_2), 2.62 (t, J = 6.5 Hz, 2H, CH_2), 2.12 (quintet, J = 6.0 Hz, 2H, CH_2). ^{13}C NMR ($CDCl_3$): δ 196.54 (C=O), 162.68 (C, Ar), 147.70 (C,

Ar), 137.73 (C, Ar), 131.49 (C, Ar), 131.33 (2 x CH, Ar), 129.17 (CH, Ar), 128.71 (2 x CH, Ar), 126.25 (C, Ar), 114.08 (CH, Ar), 113.65 (CH, Ar), 68.56 (CH_2), 38.84 (CH_2), 34.46 (CH_2), 29.73 (CH_2), 23.41 (CH_2). Anal. Calcd: C 71.02%, H 5.63%. Found: C 70.65 %, H 5.59 %.

6-(4-Bromophenethoxy)-3,4-dihydronaphthalen-1(2*H*)-one (7d)

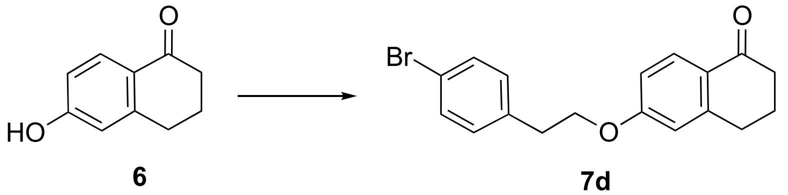

6 **7d**

Prepared from 6-hydroxytetralone (**6**) (1.0 g, 6.0 mmol) and 4-bromophenethyl bromide (1.4 mL, 9 mmol). The pure compound was eluted with petroleum ether – EtOAc 80:20 v/v to give a yellow oil. Yield: 0.59 g (29%), TLC (petroleum ether - EtOAc 3:1 v/v) R_f 0.39. ^{1}H NMR ($CDCl_3$): δ 8.02 (d, *J* = 8.5 Hz, 1H, Ar), 7.47 (d, *J* = 8.0 Hz, 2H, Ar), 7.19 (d, *J* = 8.5 Hz, 2H, Ar), 6.83 (dd, *J* = 2.5, 9.0 Hz, 1H, Ar), 6.70 (d, *J* = 2.0 Hz, 1H, Ar), 4.22 (t, *J* = 6.5 Hz, 2H, CH_2), 3.08 (t, *J* = 6.5 Hz, 2H, CH_2), 2.93 (t, *J* = 6.0 Hz, 2H, CH_2), 2.62 (t, *J* = 7.0 Hz, 2H, CH_2), 2.13 (quintet, *J* = 6.5 Hz, 2H, CH_2). ^{13}C NMR ($CDCl_3$): δ 196.24 (C=O), 162.63 (C, Ar), 146.98 (C, Ar), 136.96 (C, Ar), 131.63 (2 x CH, Ar), 130.74 (2 x CH, Ar), 129.69 (CH, Ar), 126.45 (C, Ar), 120.54 (C, Ar), 113.38 (CH, Ar), 113.21 (CH, Ar), 68.34 (CH_2), 38.90 (CH_2), 35.05 (CH_2), 30.15 (CH_2), 23.37 (CH_2).

6-(4-Methoxyphenethoxy)-3,4-dihydronaphthalen-1(2*H*)-one (7e)

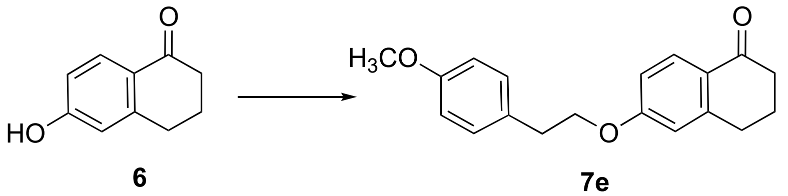

6 **7e**

Prepared from 6-hydroxytetralone (**6**) (0.5 g, 3.1 mmol) and 4-methoxyphenethyl bromide (0.73 mL, 4.65 mmol). The pure compound was eluted with petroleum ether – EtOAc 75:25

v/v. to give a green oil. Yield: 0.42 g (46%), TLC (petroleum ether - EtOAc 3:1 v/v) R_f 0.35. ^{1}H NMR ($CDCl_3$): δ 8.02 (d, *J* = 9.0 Hz, 1H, Ar), 7.23 (d, *J* = 8.0 Hz, 2H, Ar), 6.90 (d, *J* = 8.0 Hz, 2H, Ar), 6.84 (d, *J* = 8.5 Hz, 1H, Ar), 6.71 (s, 1H, Ar), 4.21 (t, *J* = 7.0 Hz, 2H, CH_2), 3.82 (s, 3H, CH_3), 3.08 (t, *J* = 7.0 Hz, 2H, CH_2), 2.92 (t, *J* = 6.0 Hz, 2H, CH_2), 2.62 (t, *J* = 6.0 Hz, 2H, CH_2), 2.13 (quintet, *J* = 6.0 Hz, 2H, CH_2). ^{13}C NMR ($CDCl_3$): δ 197.21 (C=O), 162.85 (C, Ar), 158.41 (C, Ar), 146.94 (C, Ar), 129.95 (2 x CH, Ar), 129.86 (C, Ar), 129.65 (CH, Ar), 126.32 (C, Ar), 114.00 (2 x CH, Ar), 113.49 (CH, Ar), 113.20 (CH, Ar), 69.06 (CH_2), 55.29 (OCH_3), 38.91 (CH_2), 34.76 (CH_2), 30.16 (CH_2), 23.39 (CH_2). HPLC: 100%, RT= 4.82 min. HRMS (ESI, m/z): Theoretical mass: 319.1310 $[M+Na]^+$, observed mass: 319.1312 $[M+Na]^+$.

6-Phenylpropoxy-3,4-dihydronaphthalen-1(2*H*)-one (7f)

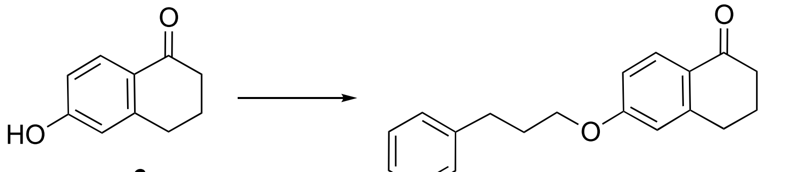

6 → 7f

Prepared from 6-hydroxytetralone (**6**) (1.3 g, 8.0 mmol) and phenylpropyl bromide (1.8 mL, 12 mmol). The pure compound was eluted with petroleum ether – EtOAc 80:20 v/v to give a biege solid. Yield: 1.91 g (85%), m.p: 66-68 °C (Lit. m.p. 93-94 °C)[8], TLC (petroleum ether - EtOAc 3:1 v/v) R_f 0.43. ^{1}H NMR ($CDCl_3$): δ 8.03 (d, *J* = 8.5 Hz, 1H, Ar), 7.30 (m, 5H, Ar), 6.83 (d, *J* = 9.0 Hz, 1H, Ar), 6.71 (s, 1H, Ar), 4.04 (t, *J* = 6.0 Hz, 2H, CH_2), 2.93 (t, *J* = 6.0 Hz, 2H, CH_2), 2.84 (t, *J* = 7.5 Hz, 2H, CH_2), 2.63 (t, *J* = 6.5 Hz, 2H, CH_2), 2.15 (m, 2H, CH_2), 2.13 (m, 2H, CH_2).

6-(3-(4-Fluorophenyl)propoxy)-3,4-dihydronaphthalen-1(2*H*)-one (7g)

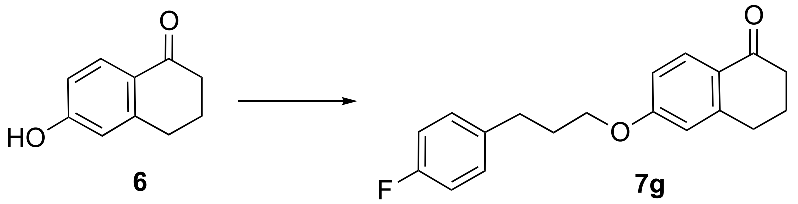

Prepared from 6-hydroxytetralone (**6**) (0.9 g, 5.5 mmol) and 4-fluorophenylpropyl bromide (1.8 g, 8.3 mmol). The pure compound was eluted with petroleum ether – EtOAc 75:25 v/v to give a yellow oil. Yield: 1.1 g (67%), TLC (petroleum ether - EtOAc 3:1 v/v) R_f 0.54. ^{1}H NMR ($CDCl_3$): δ 8.03 (d, J = 8.5 Hz, 1H, Ar), 7.18 (m, 2H, Ar), 6.90 (t, J = 8.5 Hz, 2H, Ar), 6.83 (dd, J = 2.5, 9.0 Hz, 1H, Ar), 6.70 (d, J = 2.5 Hz, 1H, Ar), 4.02 (t, J = 6.0 Hz, 2H, CH_2), 2.93 (t, J = 6.0 Hz, 2H, CH_2), 2.81 (t, J = 7.5 Hz, 2H, CH_2), 2.63 (t, J = 6.5 Hz, 2H, CH_2), 2.12 (m, 4H, 2 x CH_2). ^{19}F NMR ($CDCl_3$): δ -117.38 (Ar-F). ^{13}C NMR ($CDCl_3$): δ 197.22 (C=O), 162.96 (C, Ar), 162.35 and 160.41 (d, $^1J_{CF}$ = 243.7 Hz, C-F, Ar), 146.98 (C, Ar), 136.81 (d, $^4J_{CF}$ = 3.1 Hz, C, Ar), 129.88 (d, $^3J_{CF}$ = 7.8 Hz, 2 x CH, Ar), 129.67 (CH, Ar), 126.32 (C, Ar), 115.31 and 115.14 (d, $^2J_{CF}$ = 21.1 Hz, 2 x CH, Ar), 113.44 (CH, Ar), 113.12 (CH, Ar), 66.78 (CH_2), 38.91 (CH_2), 31.23 (CH_2), 30.75 (CH_2), 30.17 (CH_2), 23.39 (CH_2). HPLC: 100%, RT= 4.87 min. HRMS (ESI, m/z): Theoretical mass: 321.1267 $[M+Na]^+$, observed mass: 321.1247 $[M+Na]^+$.

6-(3-(4-Chlorophenyl)propoxy)-3,4-dihydronaphthalen-1(2*H*)-one (7h)

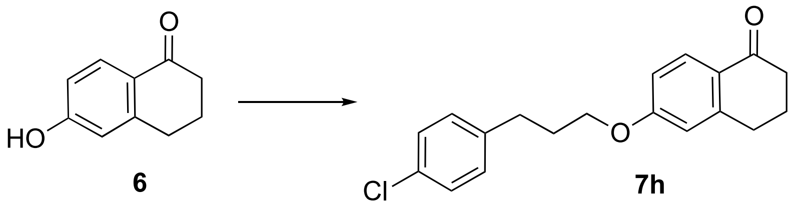

Prepared from 6-hydroxytetralone (**6**) (1.3 g, 8.0 mmol) and 4-chlorophenylpropyl bromide (2.8 g, 12 mmol). The pure compound was eluted with petroleum ether – EtOAc 80:20 v/v to give a beige solid. Yield: 2.03 g (81%), m.p: 80-82 °C, TLC (petroleum ether - EtOAc 3:1 v/v)

R_f 0.32. ^{1}H NMR ($CDCl_3$): δ 8.03 (d, *J* = 8.5 Hz, 1H, Ar), 7.28 (d, *J* = 7.0 Hz, 2H, Ar), 7.16 (d, *J* = 8.0 Hz, 2H, Ar), 6.83 (d, *J* = 8.5 Hz, 1H, Ar), 6.69 (s, 1H, Ar), 4.02 (t, *J* = 6.0 Hz, 2H, CH_2), 2.93 (t, *J* = 6.0 Hz, 2H, CH_2), 2.81 (t, *J* = 7.5 Hz, 2H, CH_2), 2.63 (t, *J* = 6.0 Hz, 2H, CH_2), 2.12 (m, 4H, 2 x CH_2). ^{13}C NMR ($CDCl_3$): δ 197.16 (C=O), 162.92 (C, Ar), 146.96 (C, Ar), 139.63 (C, Ar), 131.82 (C, Ar), 129.86 (2 x CH, Ar), 129.67 (CH, Ar), 128.58 (2 x CH, Ar), 126.36 (C, Ar), 113.44 (CH, Ar), 113.12 (CH, Ar), 66.73 (CH_2), 38.91 (CH_2), 31.41 (CH_2), 30.54 (CH_2), 30.17 (CH_2), 23.39 (CH_2). Anal. Calcd: C 72.49%, H 6.08 %. Found: C 72.42 %, H 6.05 %.

6-(3-(4-Bromophenyl)propoxy)-3,4-dihydronaphthalen-1(2*H*)-one (7i)

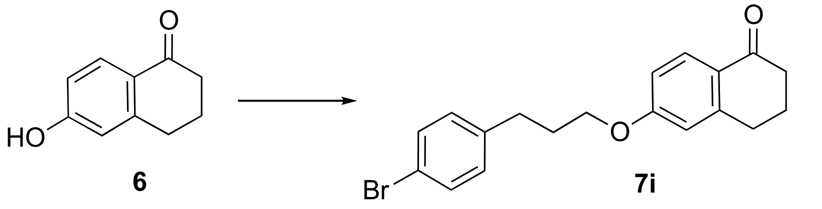

Prepared from 6-hydroxytetralone (**6**) (0.4 g, 2.4 mmol) and 4-bromophenylpropyl bromide (1.0 g, 3.6 mmol). The pure compound was eluted with petroleum ether – EtOAc 80:20 v/v to give a white solid. Yield: 0.82 g (95%), m.p: 78-80 °C, TLC (petroleum ether - EtOAc 3:1 v/v) R_f 0.55. ^{1}H NMR ($CDCl_3$): δ 8.03 (d, *J* = 9.0 Hz, 1H, Ar), 7.44 (d, *J* = 8.5 Hz, 2H, Ar), 7.11 (d, *J* = 8.5 Hz, 2H, Ar), 6.83 (dd, *J* = 2.5, 8.5 Hz, 1H, Ar), 6.69 (d, *J* = 2.0 Hz, 1H, Ar), 4.01 (t, *J* = 6.5 Hz, 2H, CH_2), 2.93 (t, *J* = 6.5 Hz, 2H, CH_2), 2.79 (t, *J* = 7.5 Hz, 2H, CH_2), 2.63 (t, *J* = 7.0 Hz, 2H, CH_2), 2.13 (m, 4H, 2 x CH_2). ^{13}C NMR ($CDCl_3$): δ 197.22 (C=O), 162.91 (C, Ar), 146.98 (C, Ar), 140.16 (C, Ar), 131.54 (2 x CH, Ar), 130.28 (2 x CH, Ar), 129.67 (CH, Ar), 126.34 (C, Ar), 119.82 (C, Ar), 113.44 (CH, Ar), 113.11 (CH, Ar), 66.70 (CH_2), 38.91 (CH_2), 31.48 (CH_2), 30.48 (CH_2), 30.17 (CH_2), 23.39 (CH_2). Anal. Calcd: C 63.52%, H 5.33 %. Found: C 63.42 %, H 5.25 %.

6-(3-(4-Methoxyphenyl)propoxy)-3,4-dihydronaphthalen-1(2*H*)-one (7j)

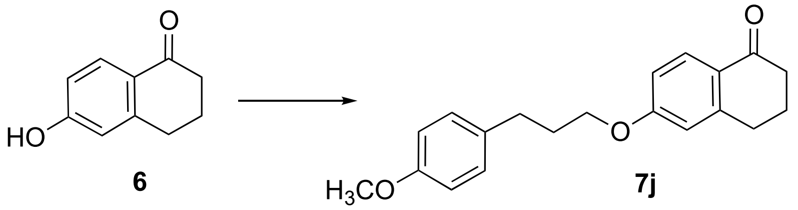

Prepared from 6-hydroxytetralone (**6**) (0.5 g, 3.0 mmol) and 4-methoxyphenylpropyl bromide (1.0 g, 4.4 mmol). The pure compound was eluted with petroleum ether – EtOAc 80:20 v/v to give a pale yellow solid. Yield: 0.93 g (83%), m.p: 68-70 °C, TLC (petroleum ether - EtOAc 3:1 v/v) R_f 0.41. ^{1}H NMR ($CDCl_3$): δ 8.03 (d, *J* = 8.5 Hz, 1H, Ar), 7.15 (d, *J* = 7.0 Hz, 2H, Ar), 6.87 (d, *J* = 8.0 Hz, 2H, Ar), 6.83 (d, *J* = 8.5 Hz, 1H, Ar), 6.70 (s, 1H, Ar), 4.01 (t, *J* = 6.0 Hz, 2H, CH_2), 3.81 (s, 3H, CH_3), 2.93 (t, *J* = 6.0 Hz, 2H, CH_2), 2.77 (t, *J* = 7.5 Hz, 2H, CH_2), 2.63 (t, *J* = 6.0 Hz, 2H, CH_2), 2.12 (m, 4H, 2 x CH_2). ^{13}C NMR ($CDCl_3$): δ 197.20 (C=O), 162.84 (C, Ar), 158.39 (C, Ar), 146.94 (C, Ar), 130.63 (C, Ar), 129.83 (2 x CH, Ar), 129.65 (CH, Ar), 126.36 (C, Ar), 114.76 (2 x CH, Ar), 113.41 (CH, Ar), 113.11 (CH, Ar), 67.81 (CH_2), 55.28 (OCH_3), 38.90 (CH_2), 31.39 (CH_2), 30.53 (CH_2), 30.12 (CH_2), 23.40 (CH_2). Anal. Calcd: C 76.95%, H 7.10 %. Found: C 76.71 %, H 7.25 %.

Antimycobacterial activity (MIC_{90}) - SPOTi Assay

Spot culture growth inhibition (SPOTi) assay was performed as previously described.[9] Briefly, each well of a flat 96-well plate was spotted with 2 µL compound, serially diluted two-fold in DMSO from 150 µg mL^{-1}. 200 µL Middlebrook 7H10 agar supplemented with 0.5 % (v/v) glycerol and 10 % OADC was dispensed into each well of a flat 96-well plate and the plate shaken to homogenise the compound. The agar was allowed to set before storage at 4 ^{o}C and used in the SPOTi assay within 24 h. To inoculate the plate, *Mtb* H37Rv was first cultured with shaking at 37 ^{o}C in Middlebrook 7H9 media supplemented with 0.5 % (v/v) glycerol, 10% OADC (Oleic Albumin Dextrose Catalase) and 0.025% Tween 80 up to an OD (optical density)

of 1. The culture was then diluted 1:100 in 7H9 media and 2 µL spotted onto each well of the SPOTi plate. Cultures were incubated at 37 °C for five weeks before MIC_{90} was recorded for each compound.

The Resazurin Microtiter Assay (REMA) Method[10]

Stock solutions of the tested compounds were prepared in sterile dimethyl sulfoxide (DMSO), then diluted in Middlebrook 7H9 broth (Difco, Detroit, MI, USA) supplemented with oleic acid, albumin, dextrose and catalase (OADC enrichment) to obtain a final compound concentration range of 0.0625–64 µg/mL. A suspension of the test *Mycobacterium* was cultured in Middlebrook 7H9 broth supplemented with 10% OADC and 0.2% glycerol, 0.2% casamino acids, 24 µg/mL pantothenate, 1 µg/mL penicillin G, 10 µg/mL cyclohexamide and 0.05% Tween 80 for one week at 37 °C in an atmosphere of 5% CO_2. The concentration was adjusted at McFarland 0.5 and diluted to 1 × 106 CFU/mL and diluted in growth media 1:25. A 100 µL of the inoculum was added to each well of a 96-well microplate together with 100 µL of the compounds. The plate was incubated at 37 °C in an atmosphere of 5% CO_2. After 5 days, 10 µL 0.2% (w/v) resazurin (solubilized in sterile water) was added. MIC_{99} was defined as the lowest concentration resulting in 99% inhibition of growth of *Mycobacterium* resulting in no colour change. Samples were set up in quadruplet and tested in two independent assays. The bacterial species and cell lines used[1] in this study can be found in Table S3.

CYP121A1 spectral binding assay for K_D determination

Recombinant CYP121A1 protein that was used in spectral binding assays was expressed and purified as previously described.[11] Compounds were initially screened for binding on a dual-beam Shimadzu 2700 spectrophotometer, in which ligand-free spectra of 1 µM CYP121A1 (50 mM TrisHCl, 300 mM NaCl, pH 7.4) were compared with spectra in the presence of 250 µM of each compound following a 15 min incubation period in a 1 cm quartz

cuvette. The shape and intensity of preliminary difference spectra indicated peak maxima between 420-435 nm and minima between 380-415 nm and were consistent with a type-II ligand response.

Full titrations were performed in triplicate at ambient temperature using a Shimadazu 2700 spectrophotometer. Protein samples at 1 μM concentration and in 1000 μL volume were prepared and titrated to acquire 12 intermediate concentration points spanning 0 to 150 μM of compound. K_D values were calculated by plotting the inhibitor concentrations against the blue-shifted type-II response. Data fitting was performed using a single binding mode equation for hyperbolic fitting in Prism GraphPad v7.05. As additional controls, cYY substrate was included in the assay using triplicate manual titration on the Shimadzu 2700 spectrophotometer.

Molecular modelling:

Molecular docking was performed using Molecular Operating Environment (MOE 2024) software.[12] Docking studies using the crystal structure of *Mtb* CYP121A1 co-crystallised with a piperazine azole derivative (PDB 5O4K),[13] which solved at high resolution (1.5 Å), were performed to generate PDB *Mtb* CYP121A1–ligand complexes, until a RMSD gradient of 0.01 kcal mol^{-1} $Å^{-1}$ with the MMFF94 forcefield (ligands) and partial charges were automatically calculated. The charge and geometry of the haem iron of the protein at physiological pH (pH 7.4) was adjusted to 3+ (geometry d2sp3) through the atom manager in MOE. The ligands were generated using either MOE builder or ChemDraw Professional (16.0), and the energy minimised and structures saved in a database for docking studies. Docking was performed using the Alpha Triangle placement to determine the poses, refinement of the results was done using the Merck molecular forcefield (MMFF94), and rescoring of the refined results using the London ΔG scoring function was applied. A pharmacophore (acceptor: azole-Fe), generated from the piperazine azole ligand of pdb 5O4K, was used to optimise docking. The output

database dock file was created showing each ligand with different poses, which were ordered according to the final S-score function.

Molecular dynamics (MD) simulation:

Molecular dynamics simulations were run on the *Mtb* CYP121A1-designed ligand complexes. PDB files were first optimised with protein preparation wizard in Maestro (Schrödinger Release 2020-1)[14] by assigning bond orders, adding hydrogen, and correcting incorrect bond types. A default quick relaxation protocol was used to minimise the MD systems with the Desmond programme. In Desmond, the volume of space in which the simulation takes place, the global cell, is built up by regular 3D simulation boxes, which was utilised as part in this system for protein interactions. The orthorhombic water box allowed for a 10 Å buffer region between protein atoms and box sides. Overlapping water molecules beyond 5 Å were deleted, and the systems were neutralised with Na^{+} ions and salt concentration 0.15 M. Force-field parameters for the complexes were assigned using the OPLS_2005 forcefield, that is, a 200 ns molecular dynamic run in the NPT ensemble (*T* 1/4 300 K) at a constant pressure of 1 bar. Energy and trajectory atomic coordinate data were recorded at each 1.2 ns.

NMR Spectra for Final Compounds

(*E*)-3-(Pyridin-3-ylmethylene)chroman-4-one (**5a**):

^{1}H NMR ($CDCl_3$)

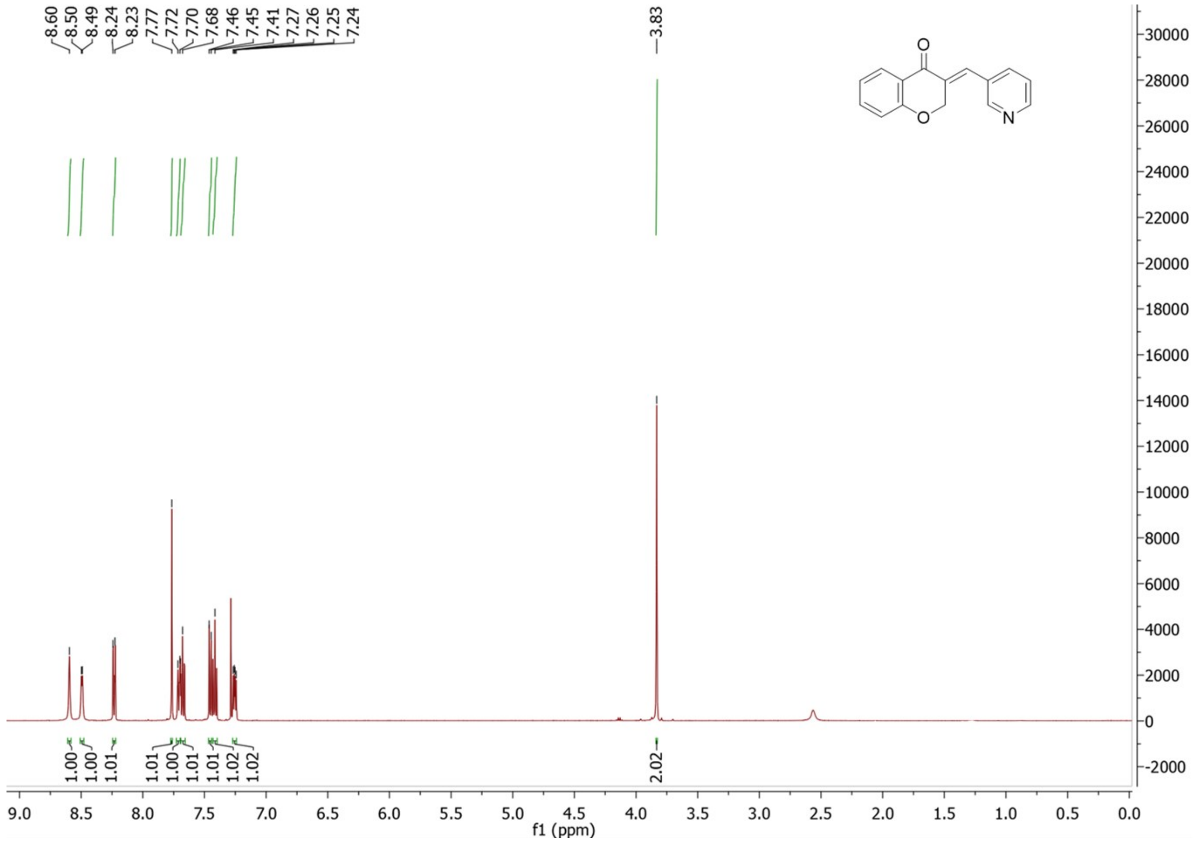

(*E*)-7-Ethoxy-3-(pyridin-3-ylmethylene)chroman-4-one (**5b**):

^{1}H NMR ($CDCl_3$)

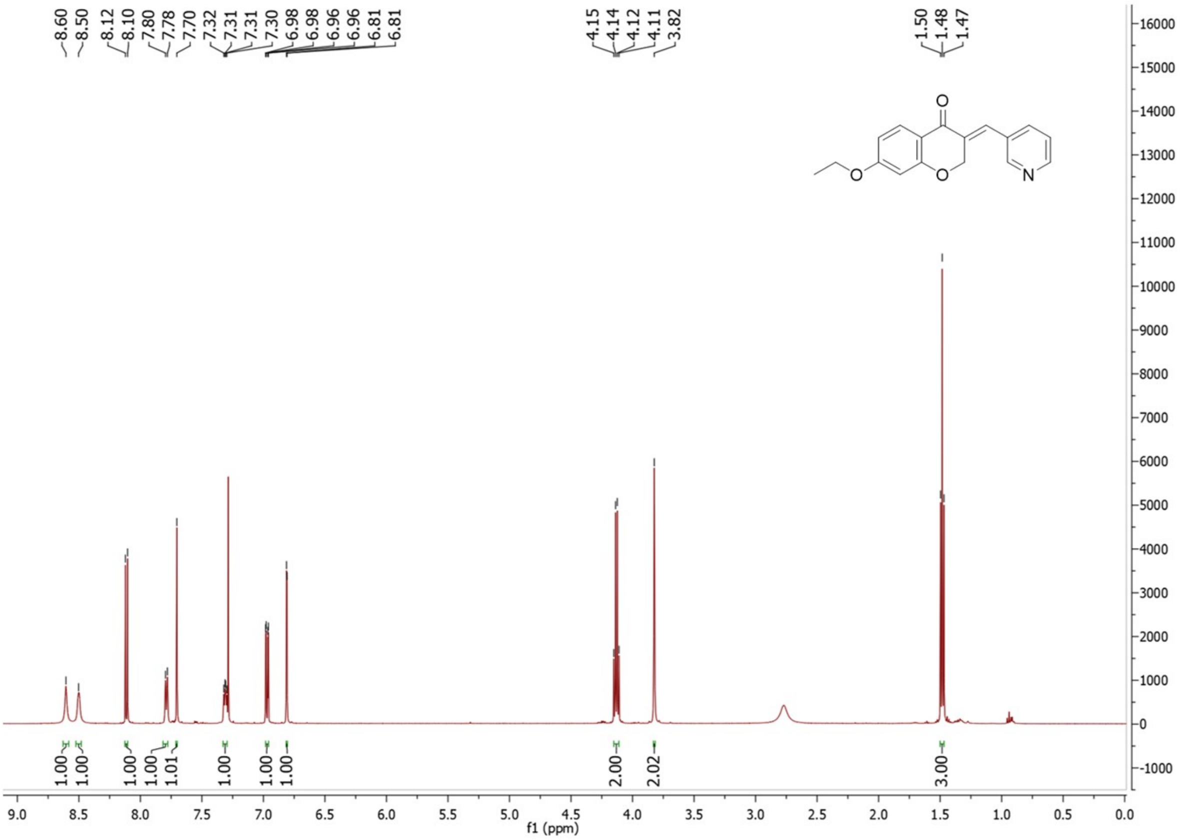

^{13}C NMR ($CDCl_3$)

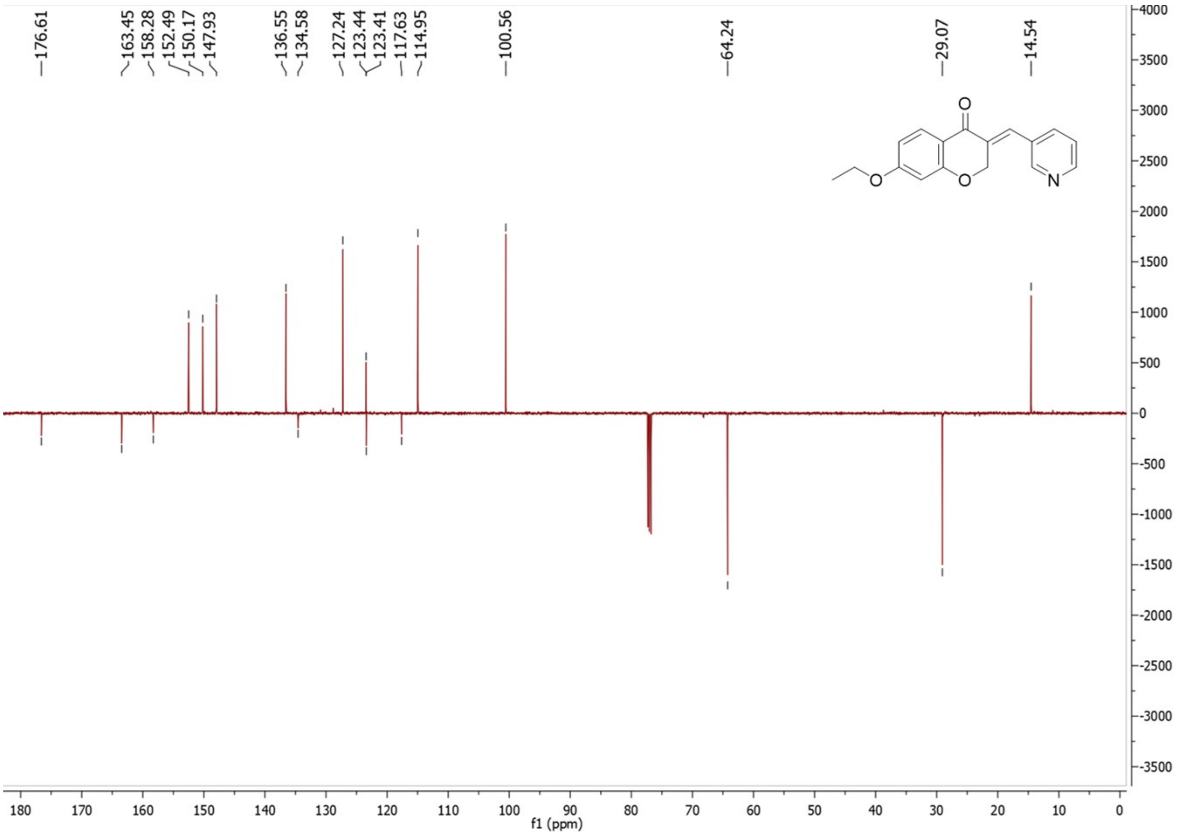

(*E*)-7-(Pentyloxy)-3-(pyridin-3-ylmethylene)chroman-4-one (**5c**):

^{1}H NMR ($CDCl_3$)

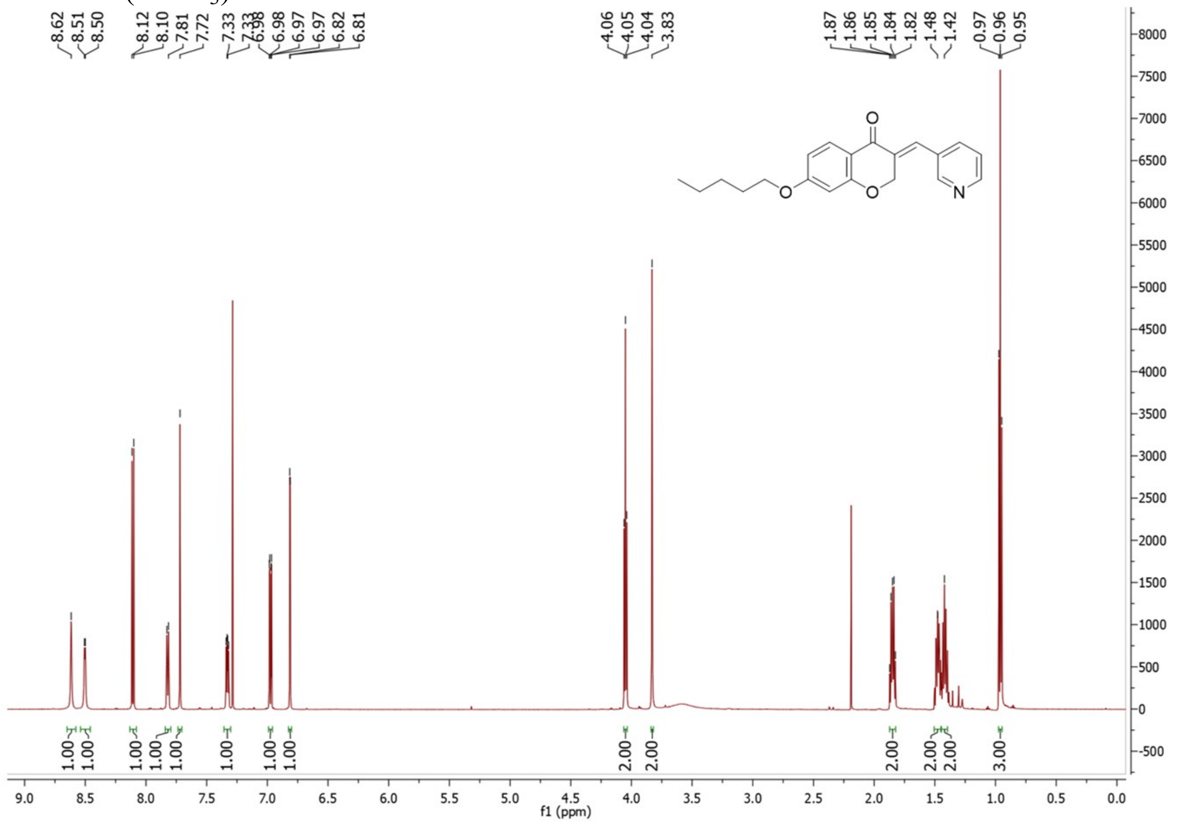

^{13}C NMR ($CDCl_3$)

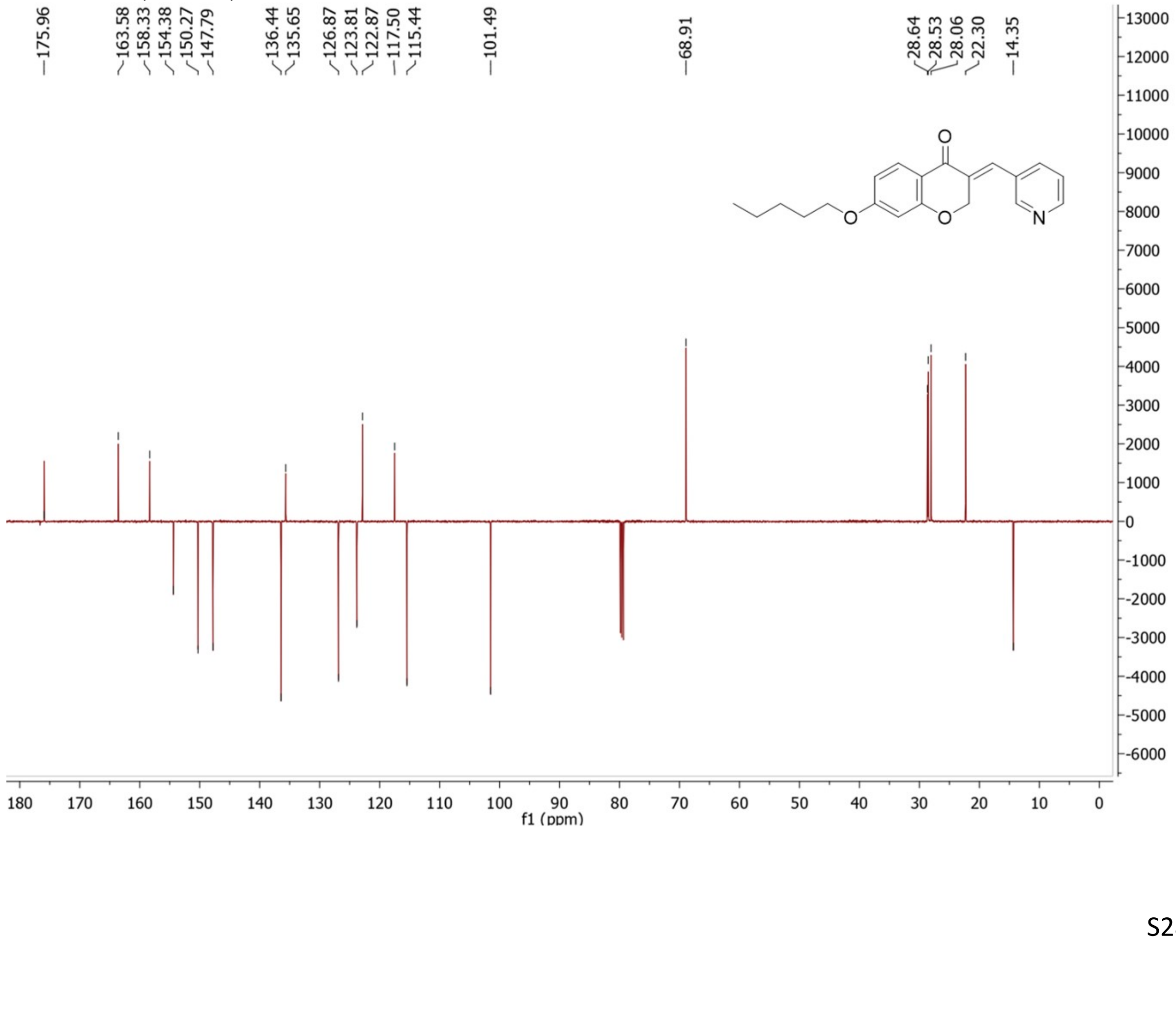

(*E*)-7-(Benzyloxy)-3-(pyridin-3-ylmethylene)chroman-4-one (**5d**):

^{1}H NMR ($CDCl_3$)

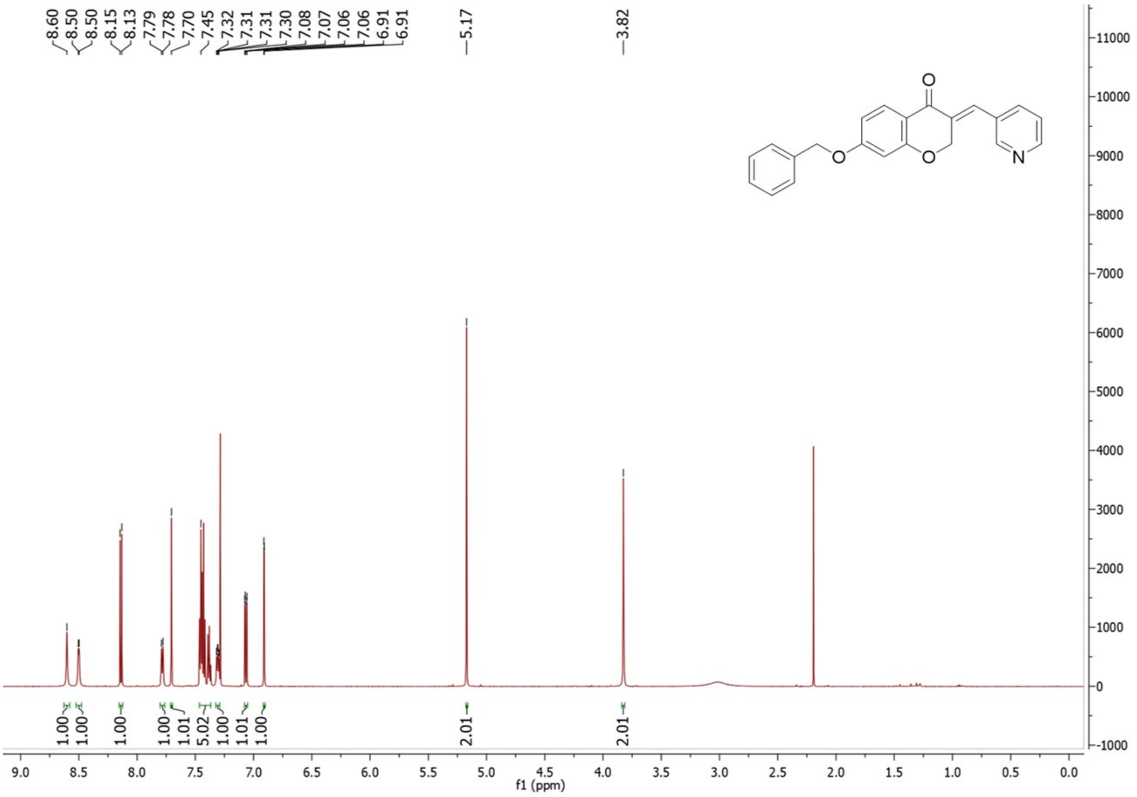

^{13}C NMR ($CDCl_3$)

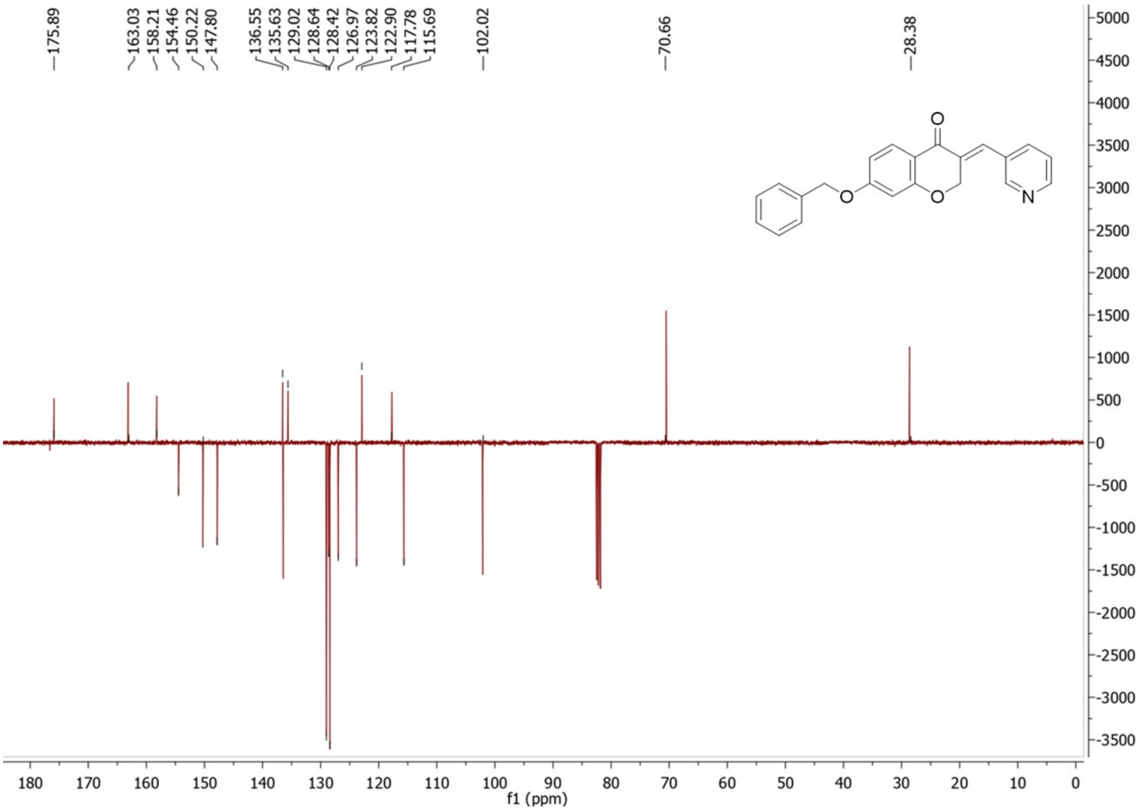

(*E*)-7-((4-fluorobenzyl)oxy)-3-(pyridin-3-ylmethylene)chroman-4-one (**5e**):

^{1}H NMR ($CDCl_3$)

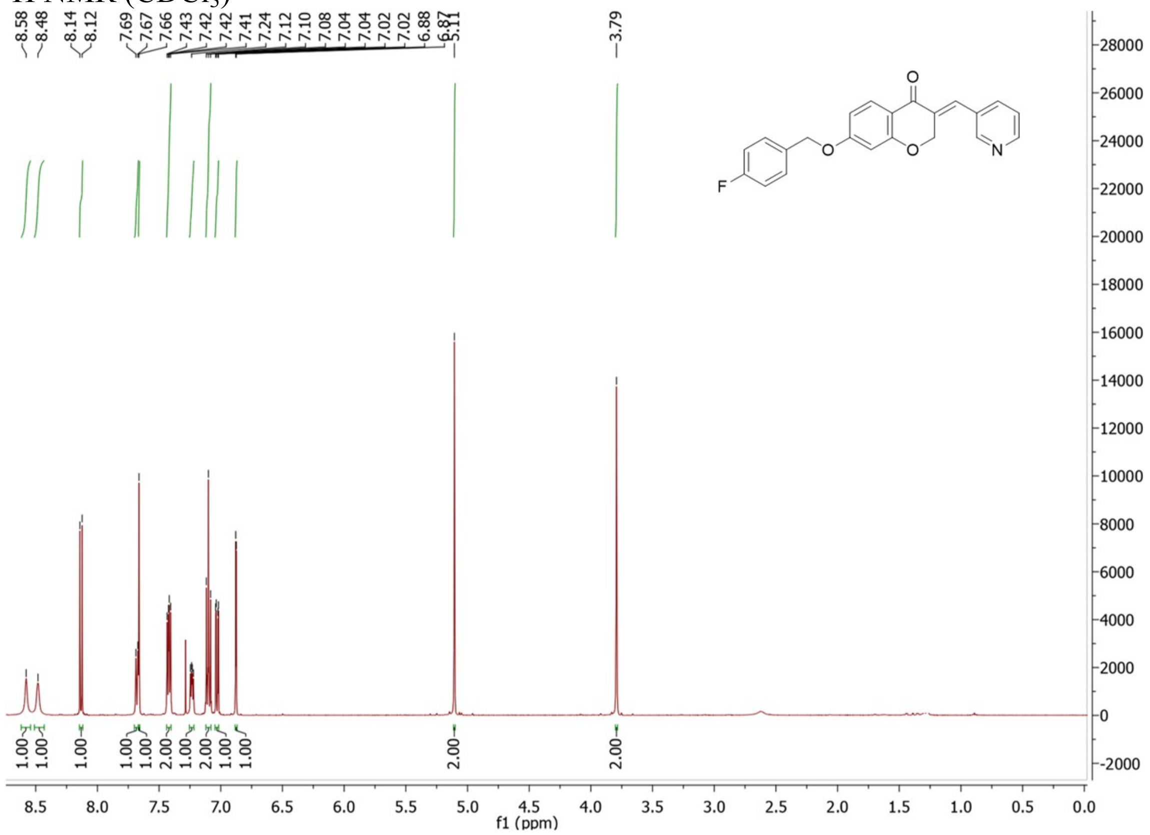

^{13}C NMR ($CDCl_3$)

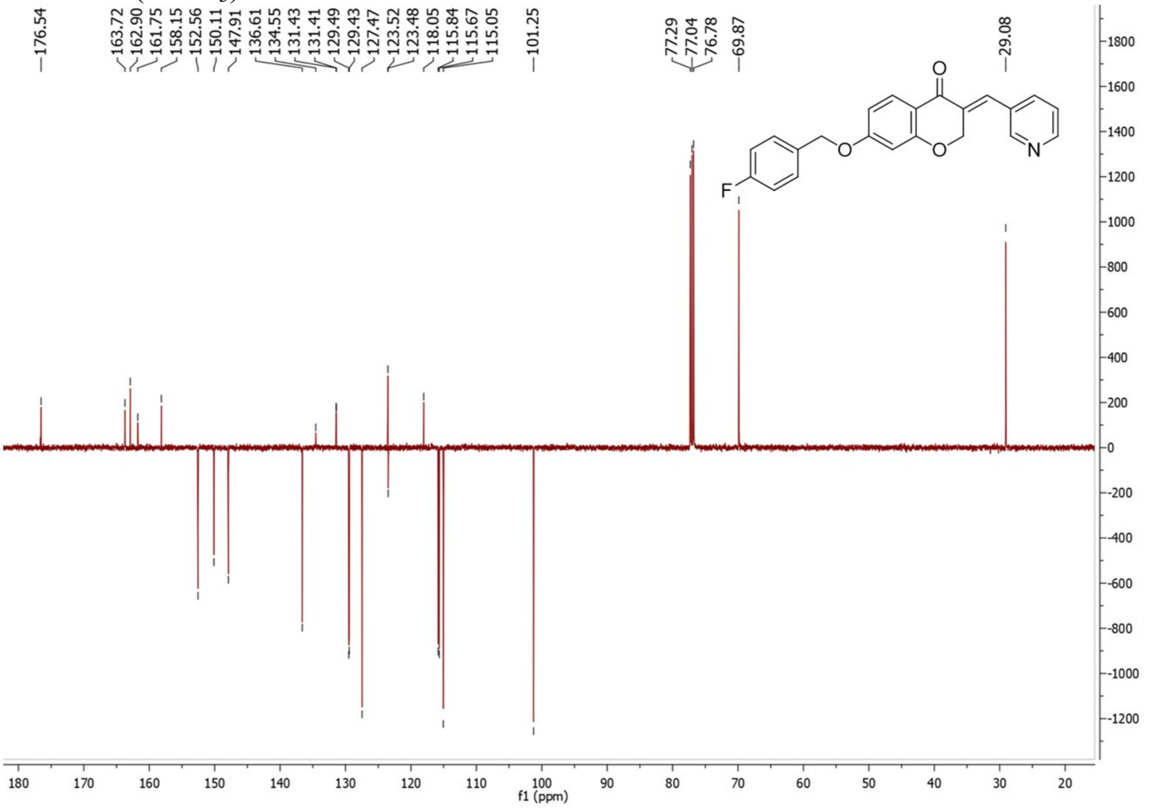

^{19}F NMR ($CDCl_3$)

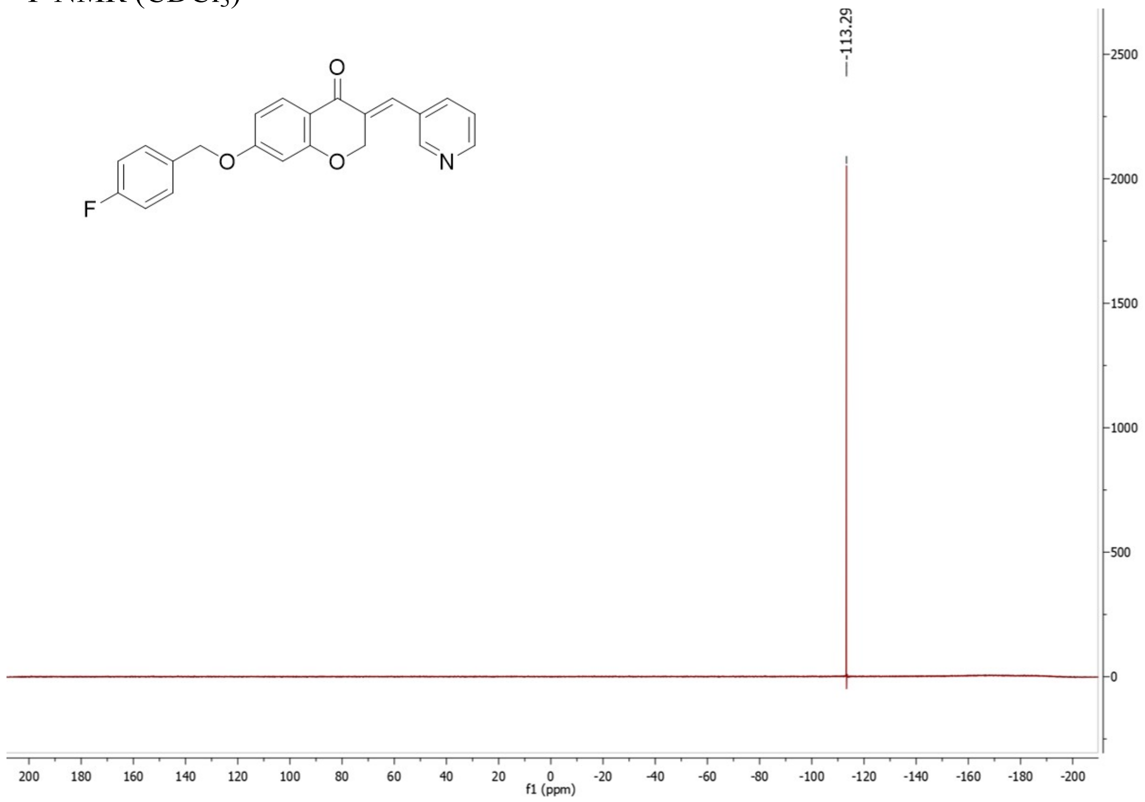

(*E*)-7-Phenethoxy-3-(pyridin-3-ylmethylene)chroman-4-one (**5f**):

^{1}H NMR ($CDCl_3$)

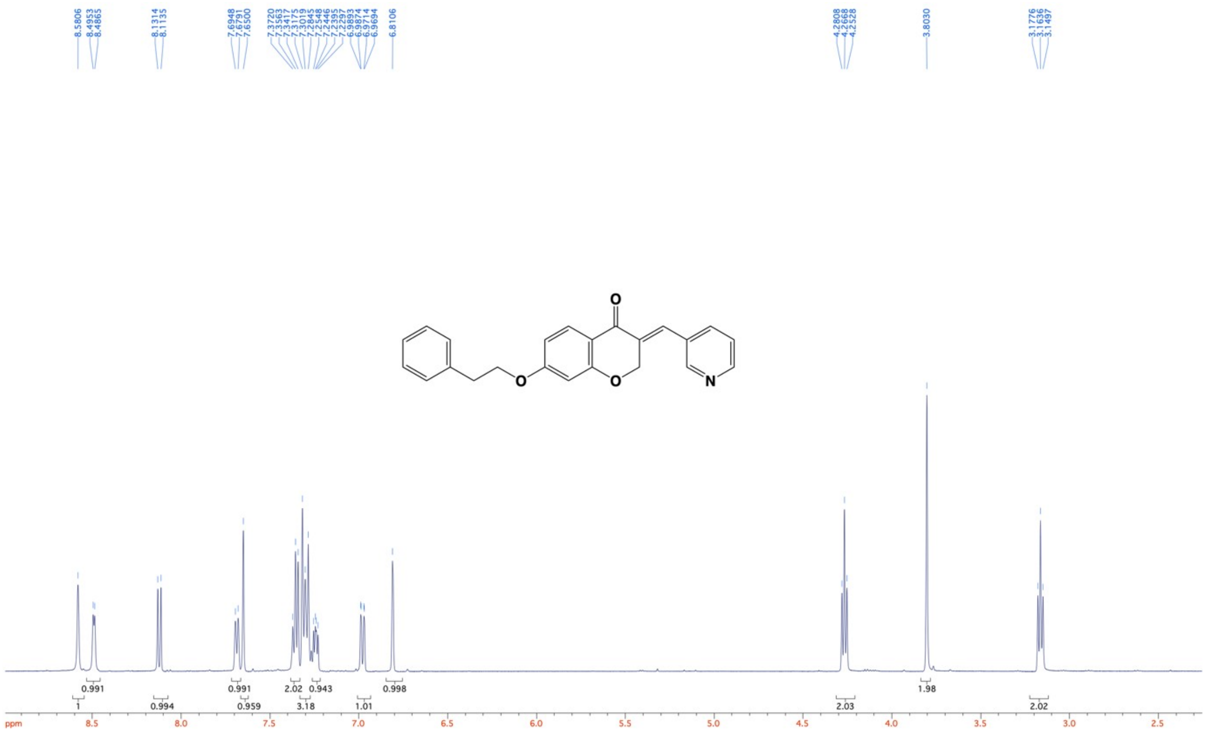

^{13}C NMR ($CDCl_3$)

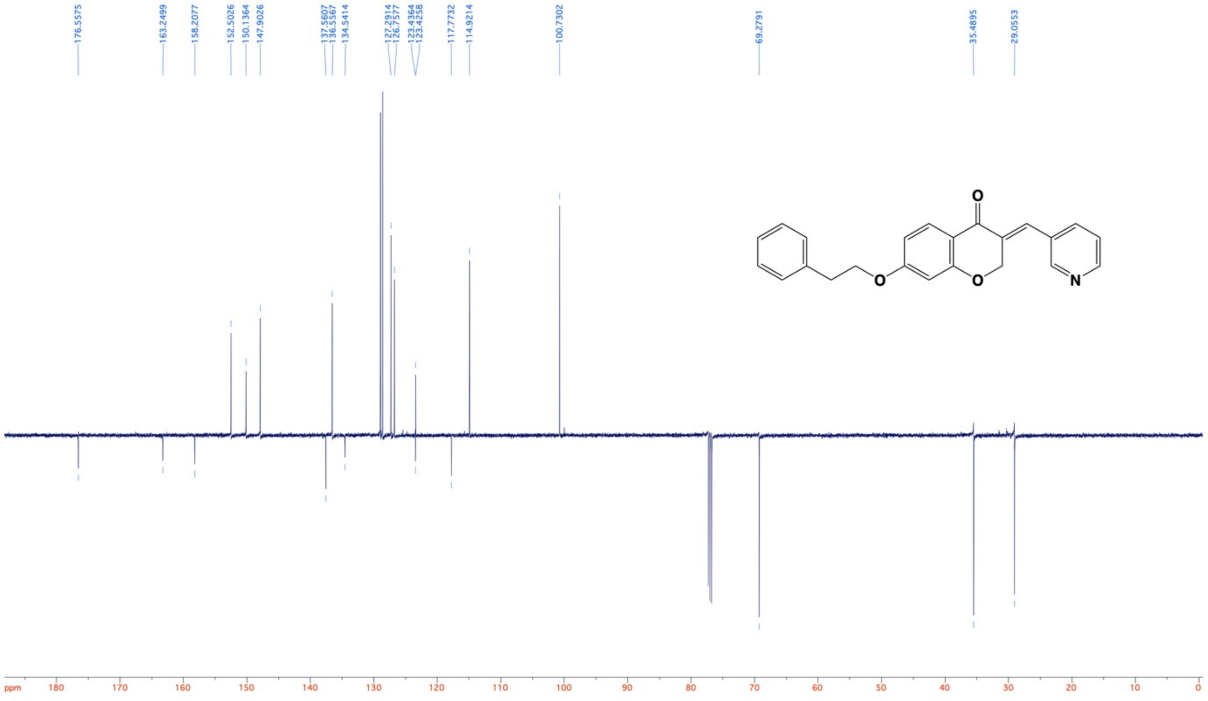

(*E*)-7-(4-Fluorophenethoxy)-3-(pyridin-3-ylmethylene)chroman-4-one (**5g**):

^{1}H NMR ($CDCl_3$)

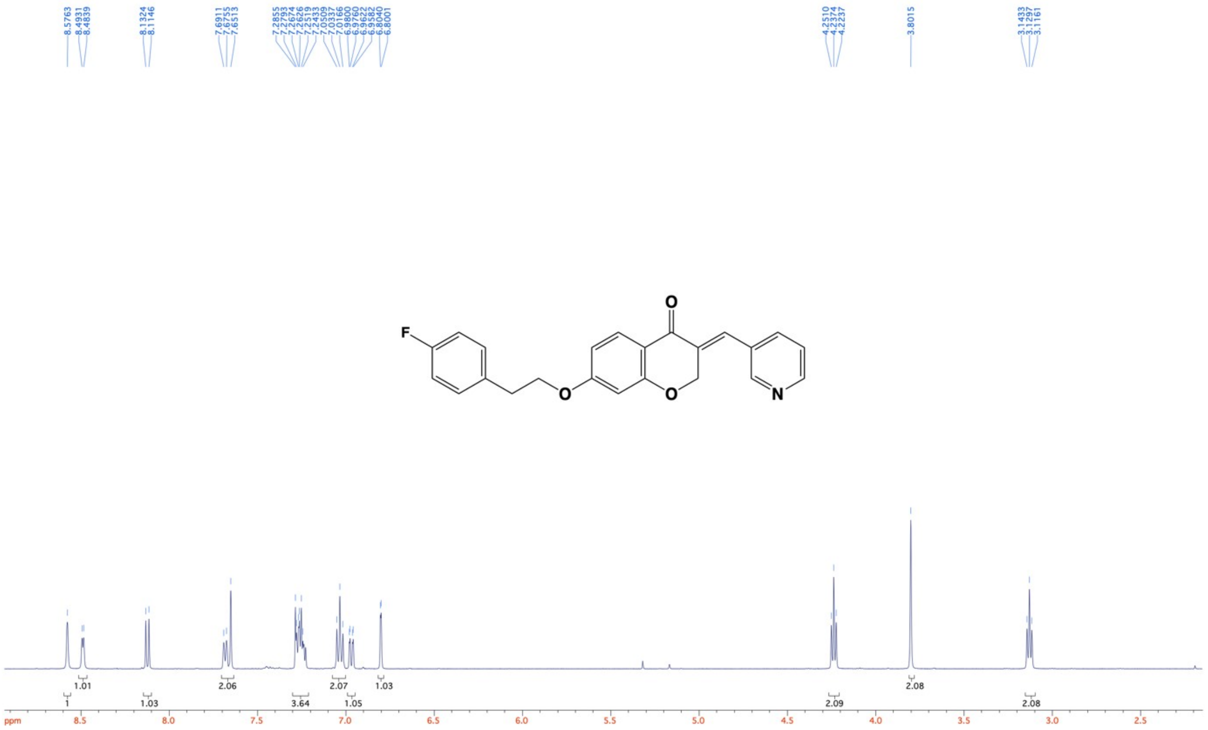

^{13}C NMR ($CDCl_3$)

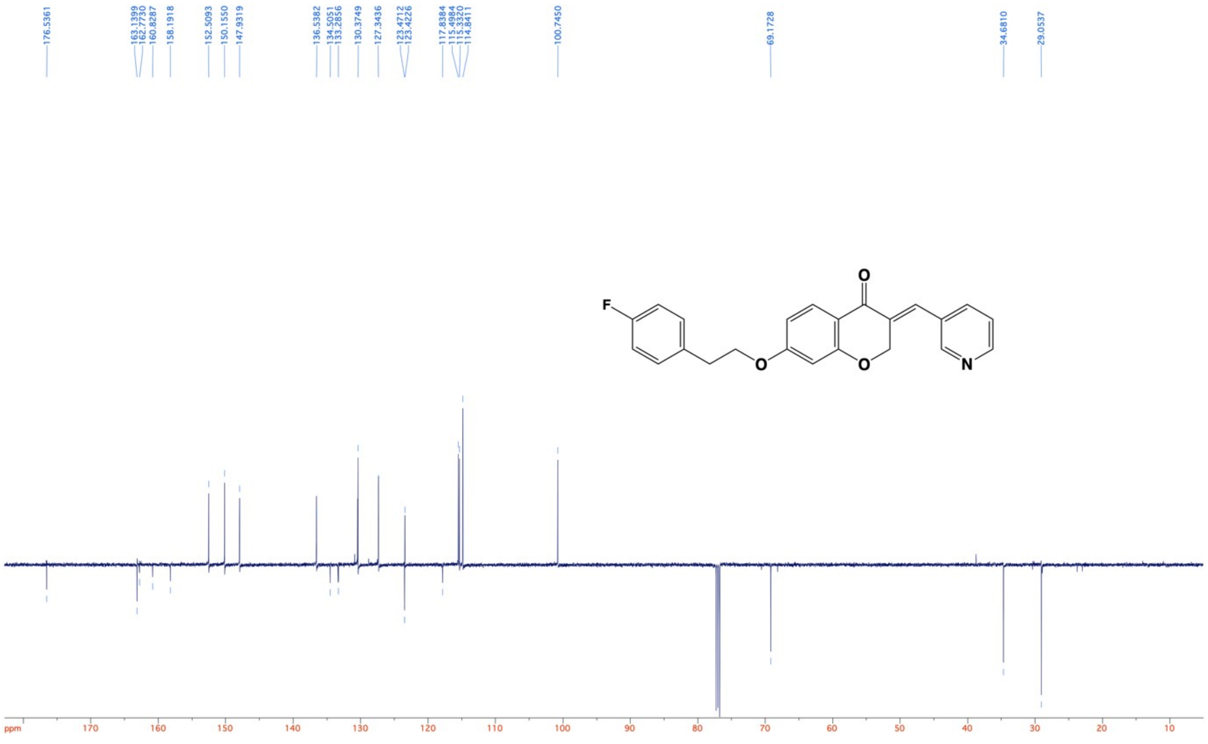

^{19}F NMR ($CDCl_3$)

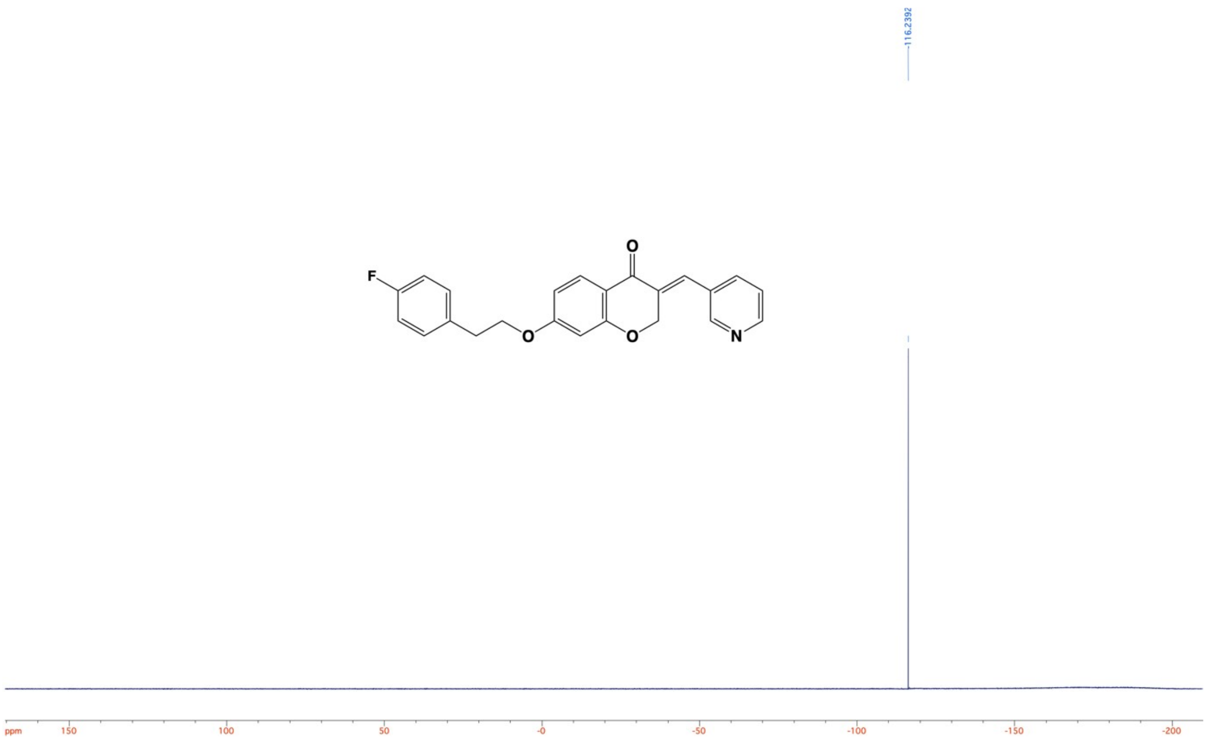

(*E*)-7-(4-Chlorophenethoxy)-3-(pyridin-3-ylmethylene)chroman-4-one (**5h**):

^{1}H NMR ($CDCl_3$)

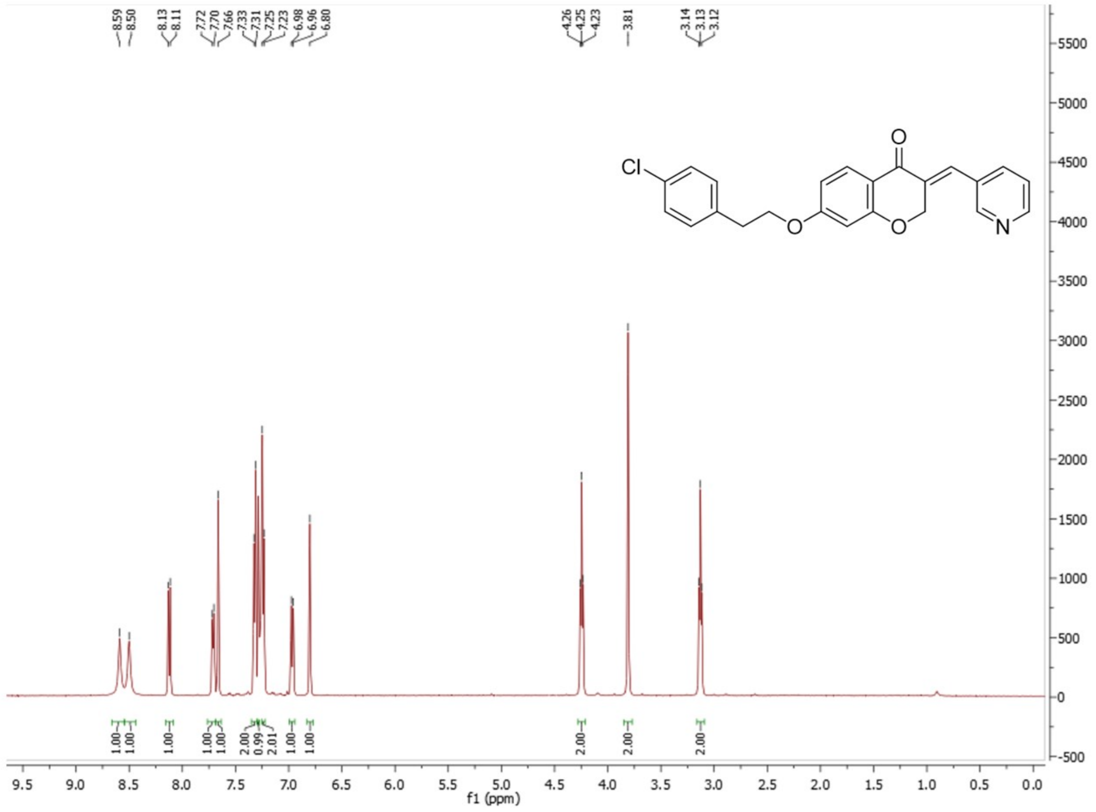

^{13}C NMR ($CDCl_3$)

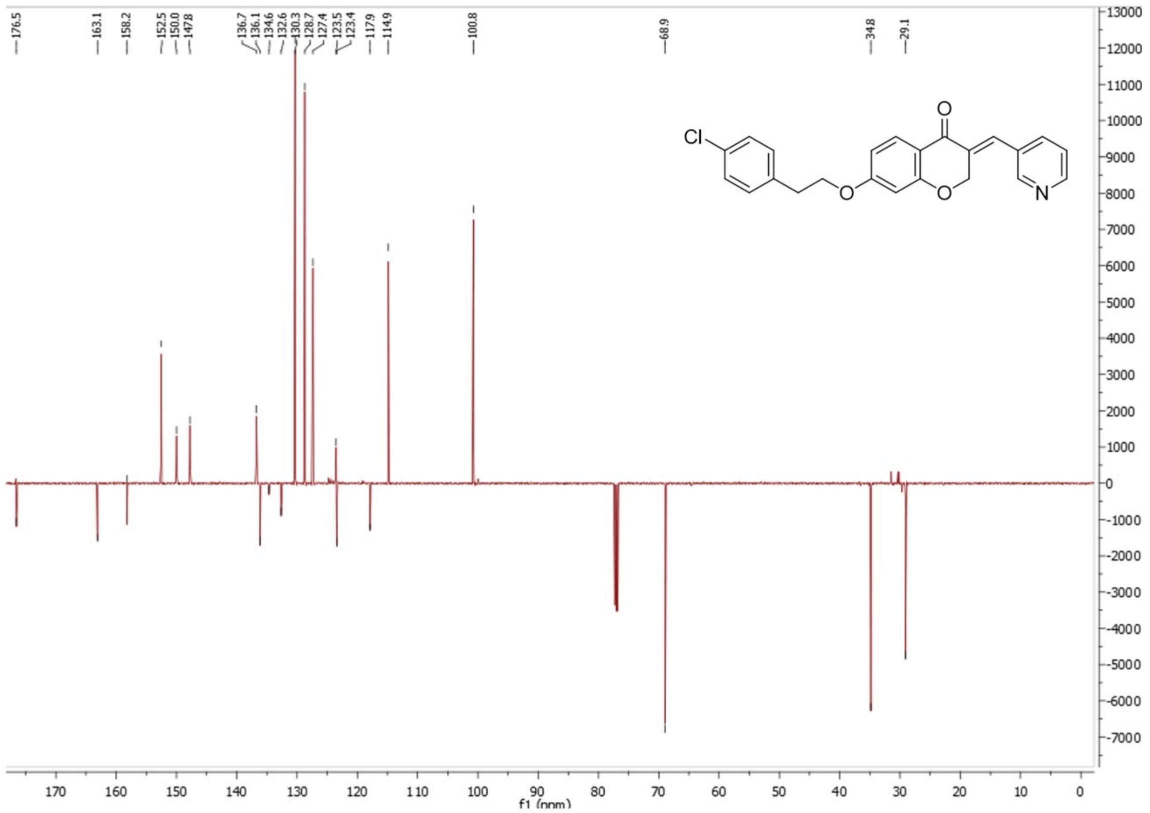

(*E*)-7-(4-Bromophenethoxy)-3-(pyridin-3-ylmethylene)chroman-4-one (**5i**)

^{1}H NMR ($CDCl_3$)

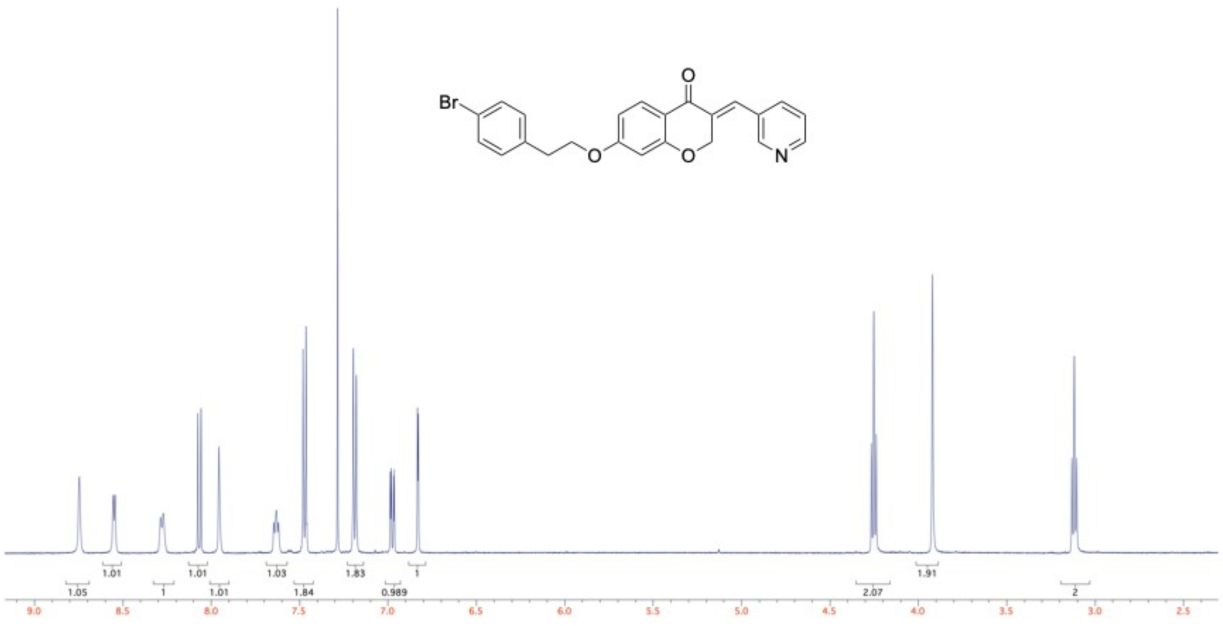

^{13}C NMR ($CDCl_3$)

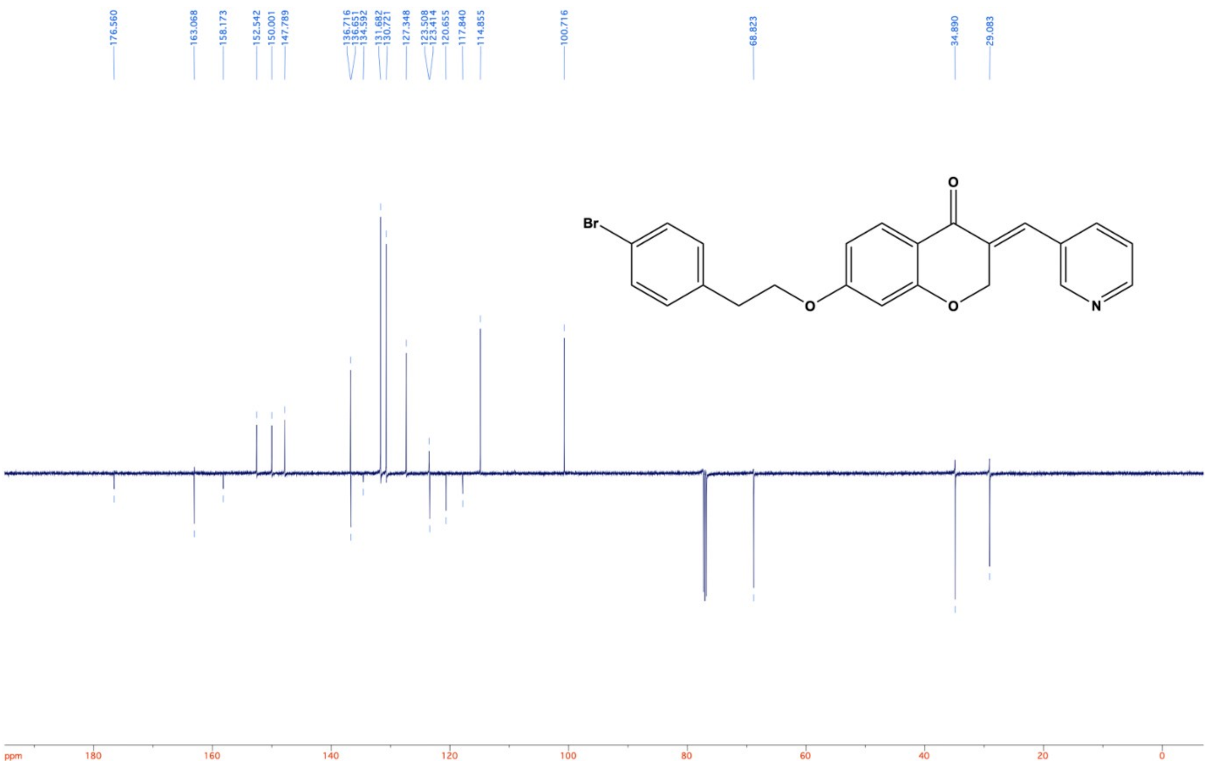

HPLC

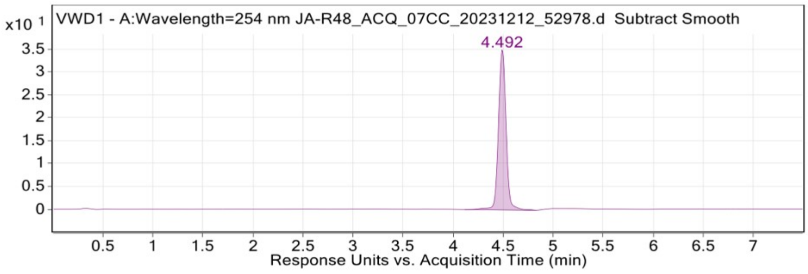

Figure: Base peak or HPLC chromatogram (indicated in left hand corner)

User Chromatogram Peak List

RT (min)	Area	Area %	Area Sum (%)	Width (min)
4.49	185.63	100.00	100.00	0.082

(*E*)-7-(4-Methoxyphenethoxy)-3-(pyridin-3-ylmethylene)chroman-4-one (**5j**):

^{1}H NMR ($CDCl_3$)

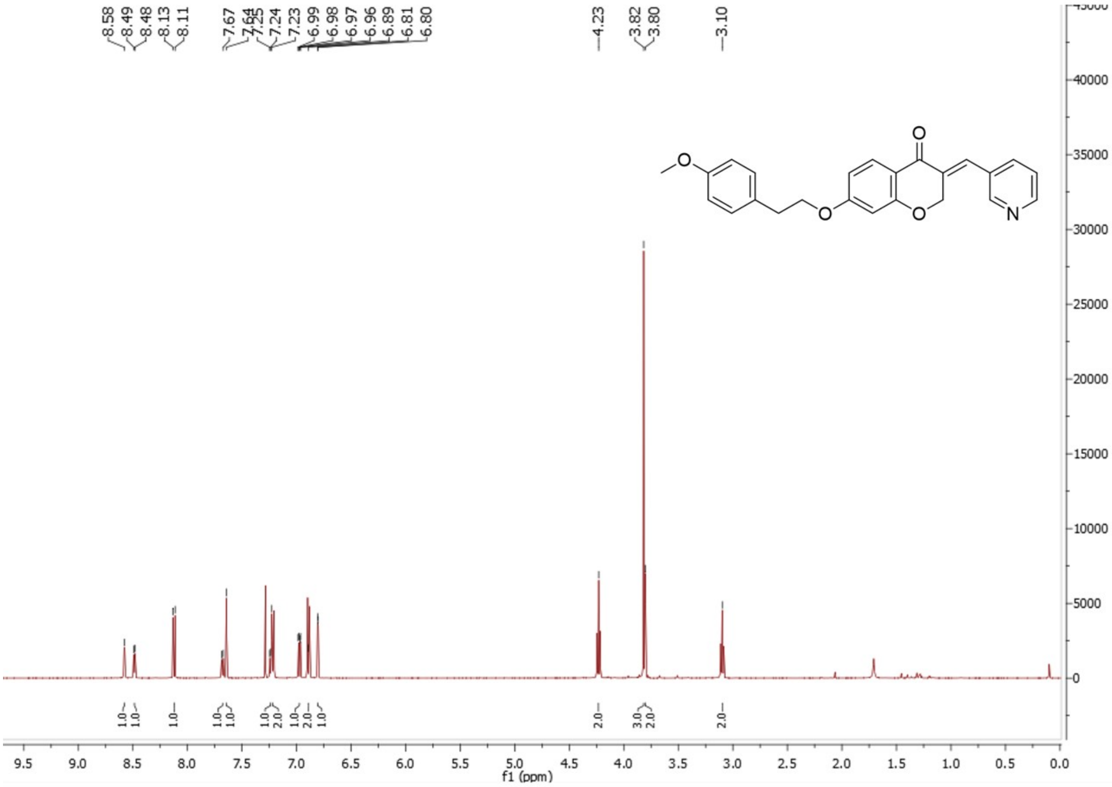

^{13}C NMR ($CDCl_3$)

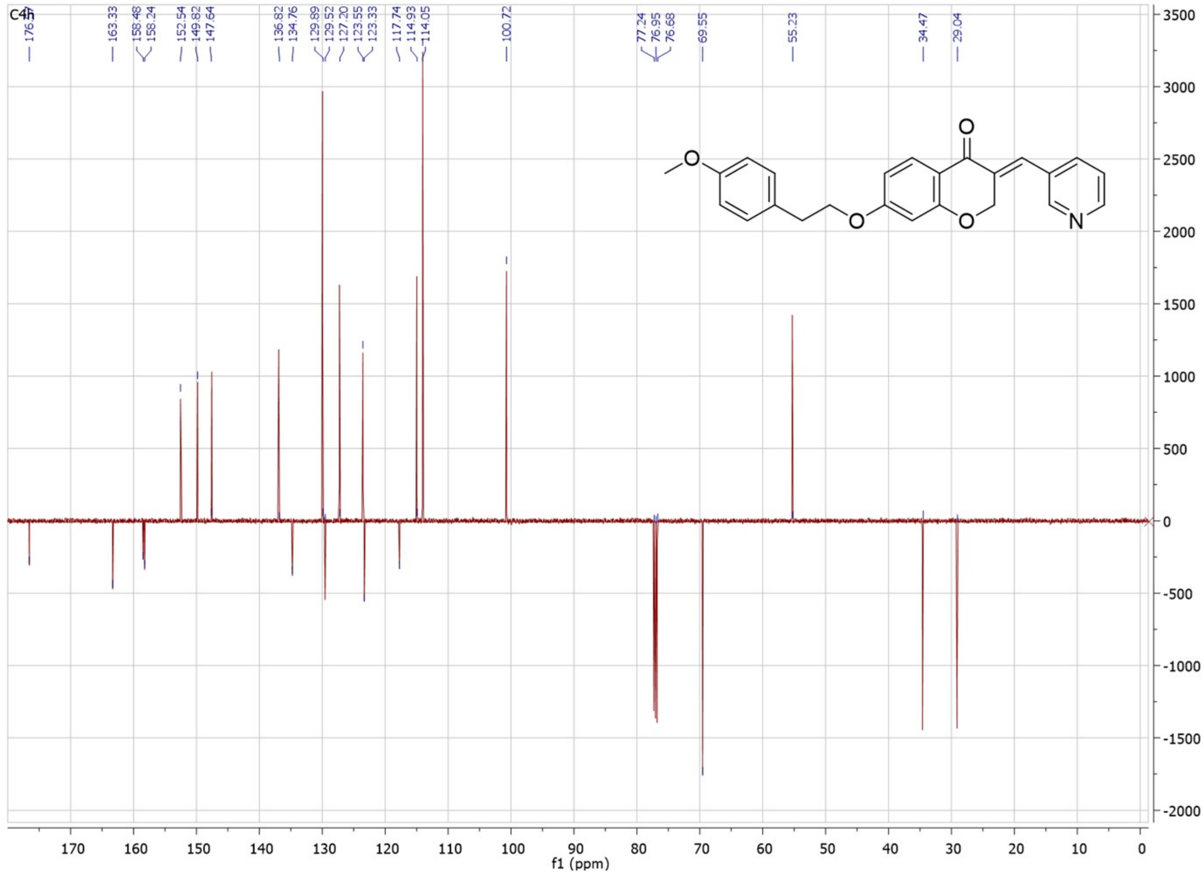

(*E*)-7-(3-Phenylpropoxy)-3-(pyridin-3-ylmethylene)chroman-4-one (**5k**):

^{1}H NMR ($CDCl_3$)

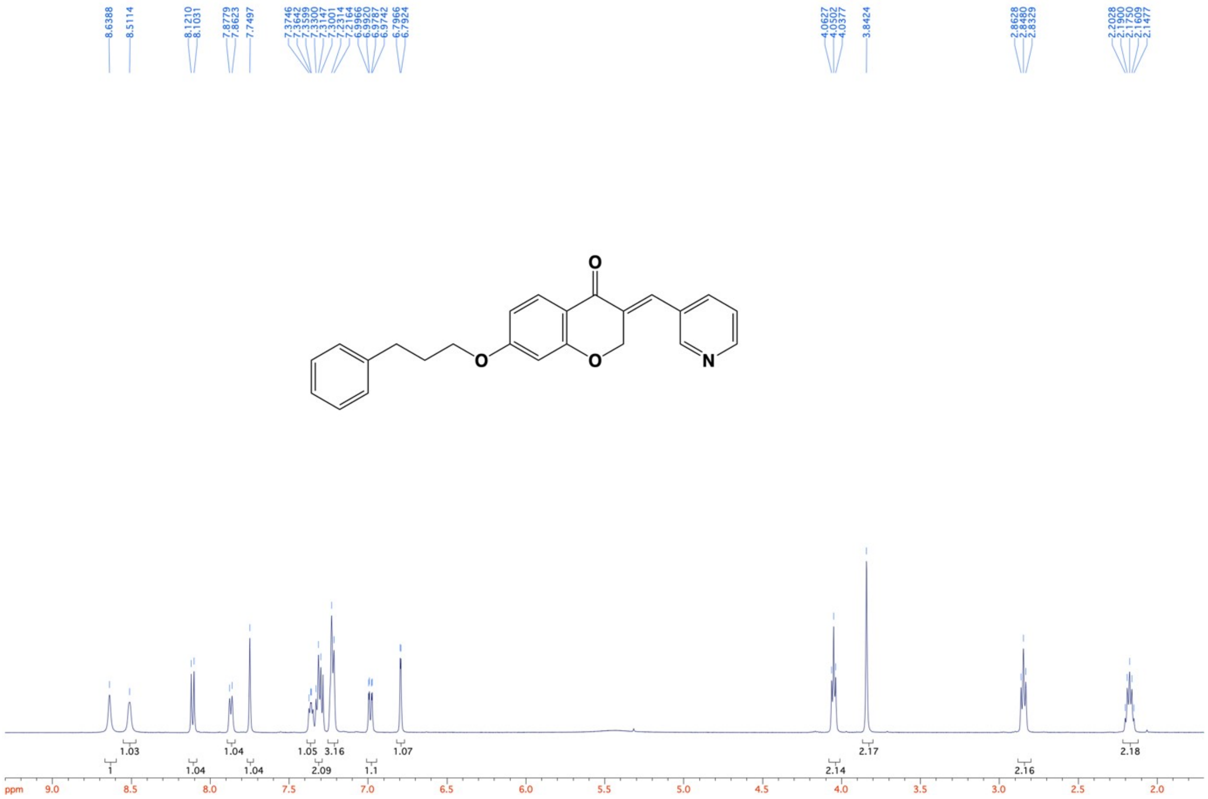

^{13}C NMR ($CDCl_3$)

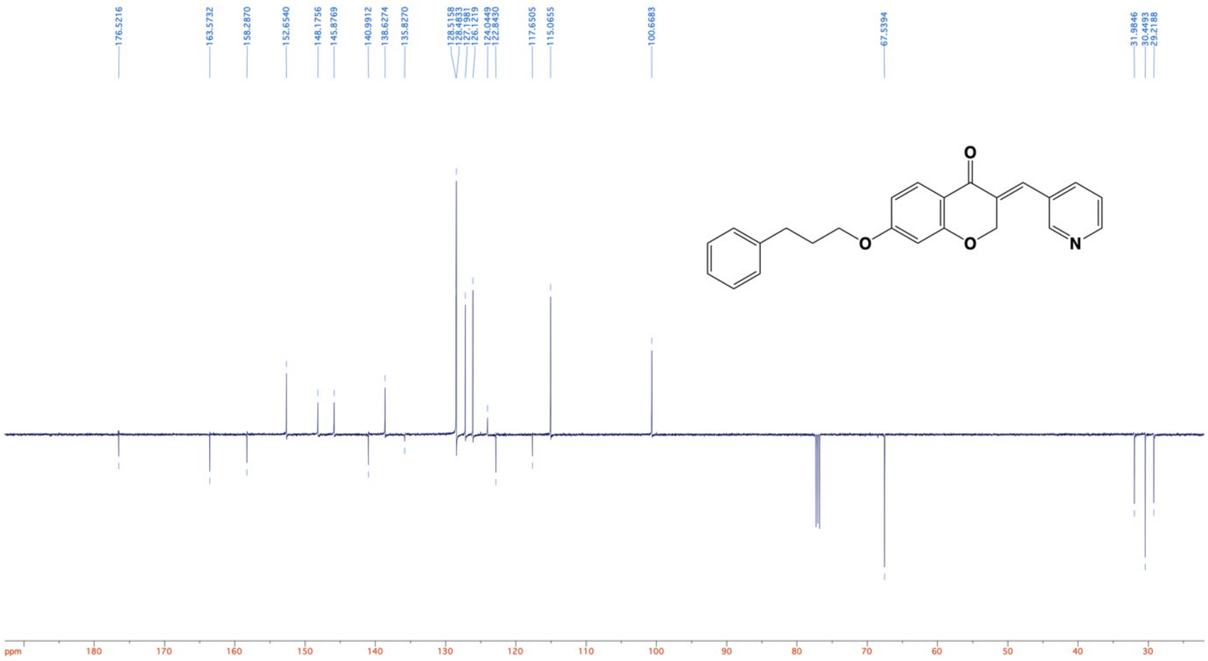

(*E*)-7-(3-(4-Fluorophenyl)propoxy)-3-(pyridin-3-ylmethylene)chroman-4-one (**5l**):

^{1}H NMR ($CDCl_3$)

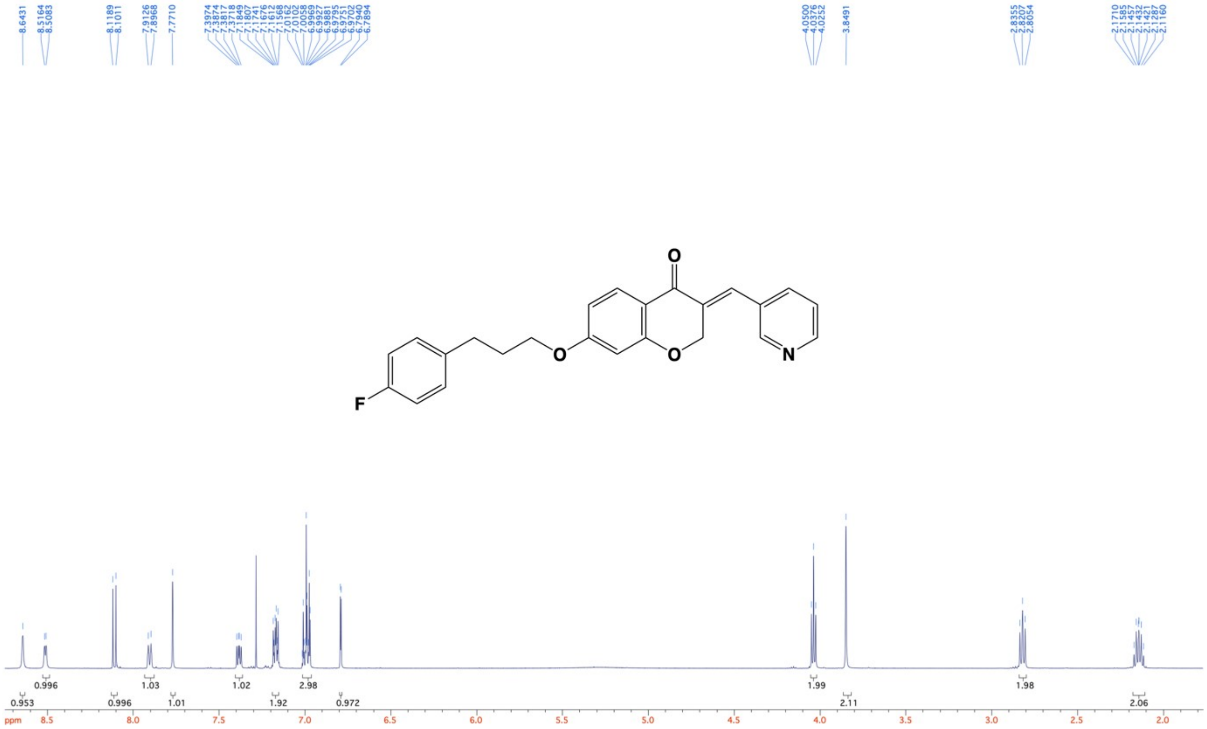

^{13}C NMR ($CDCl_3$):

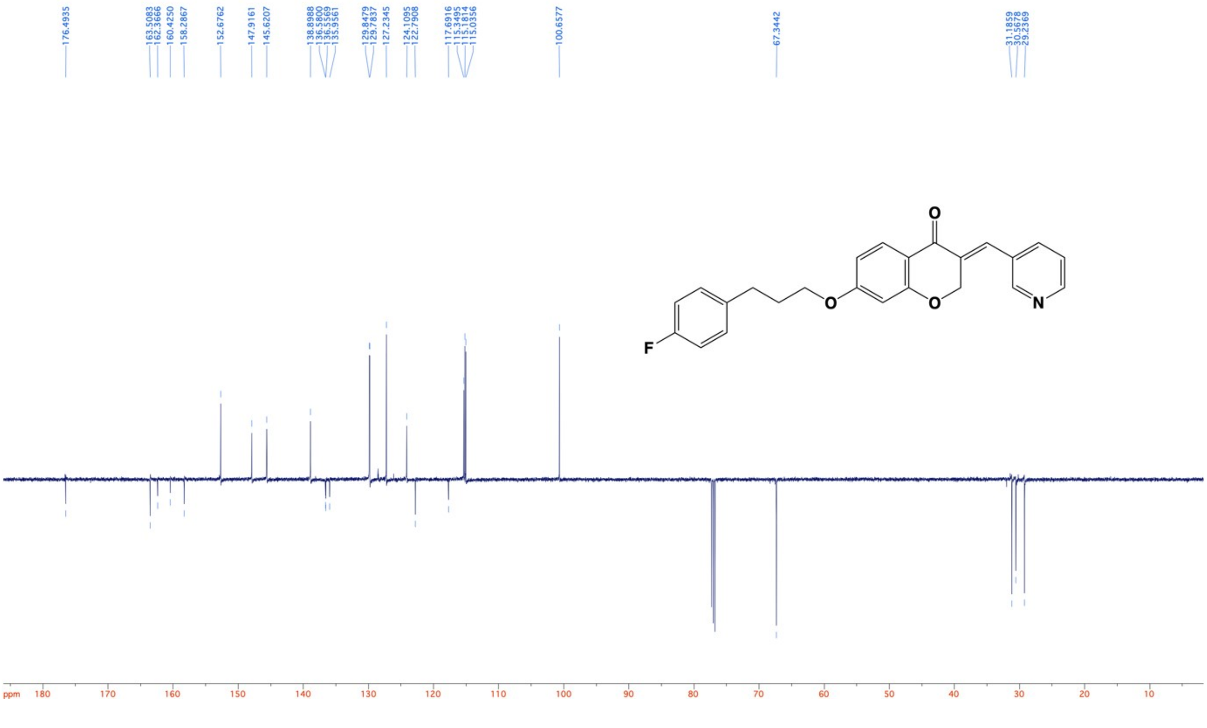

^{19}F NMR ($CDCl_3$)

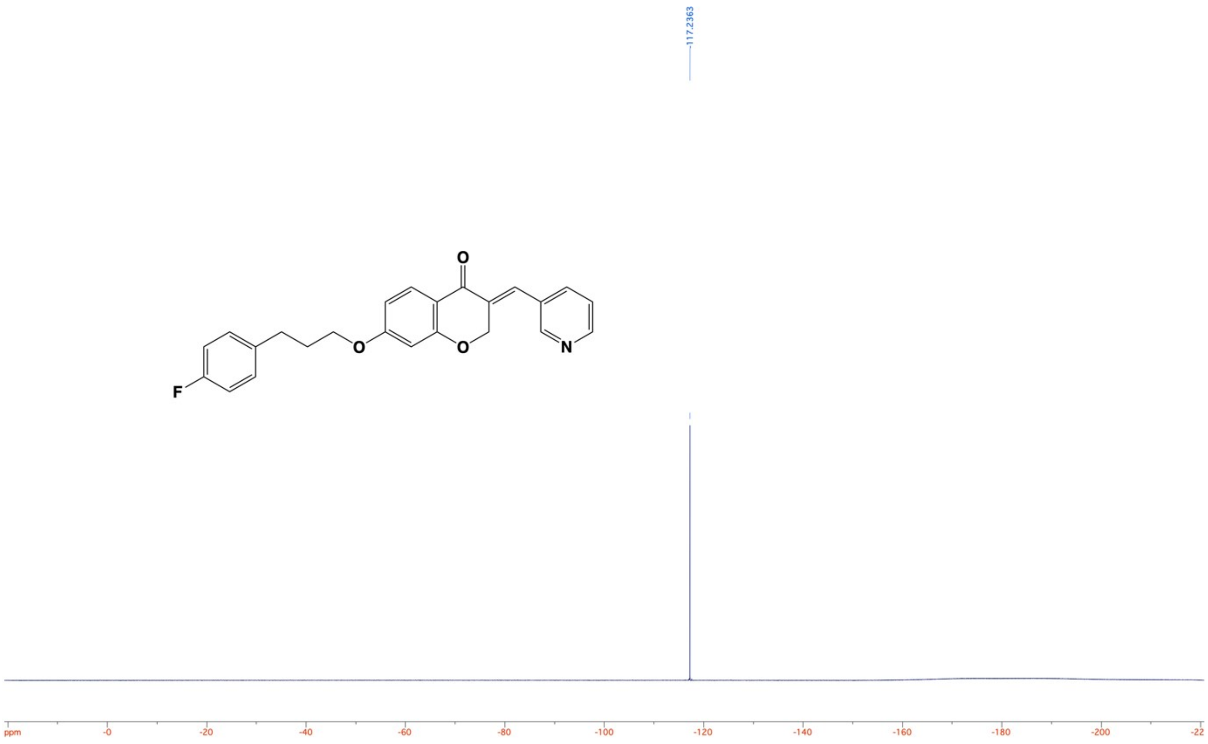

(*E*)-7-(3-(4-Chlorophenyl)propoxy)-3-(pyridin-3-ylmethylene)chroman-4-one (**5m**):

^{1}H NMR ($CDCl_3$)

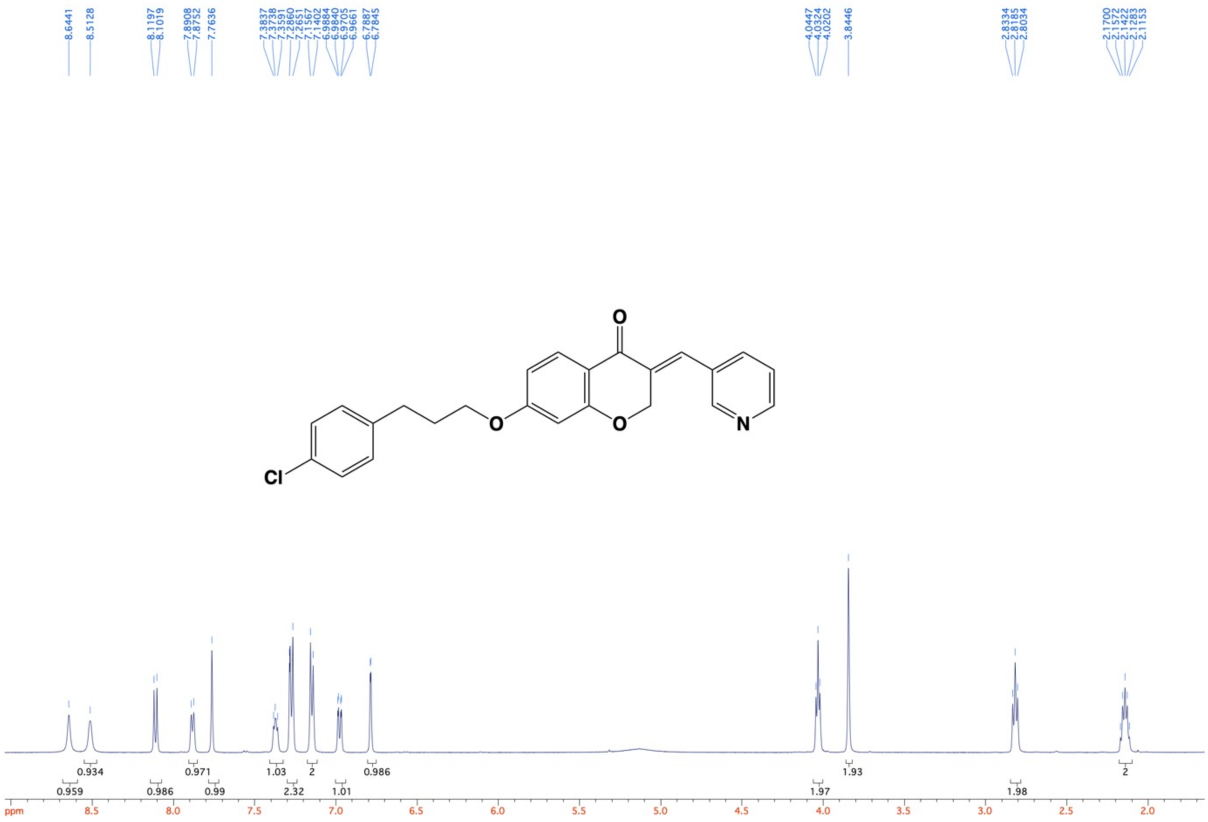

^{13}C NMR ($CDCl_3$)

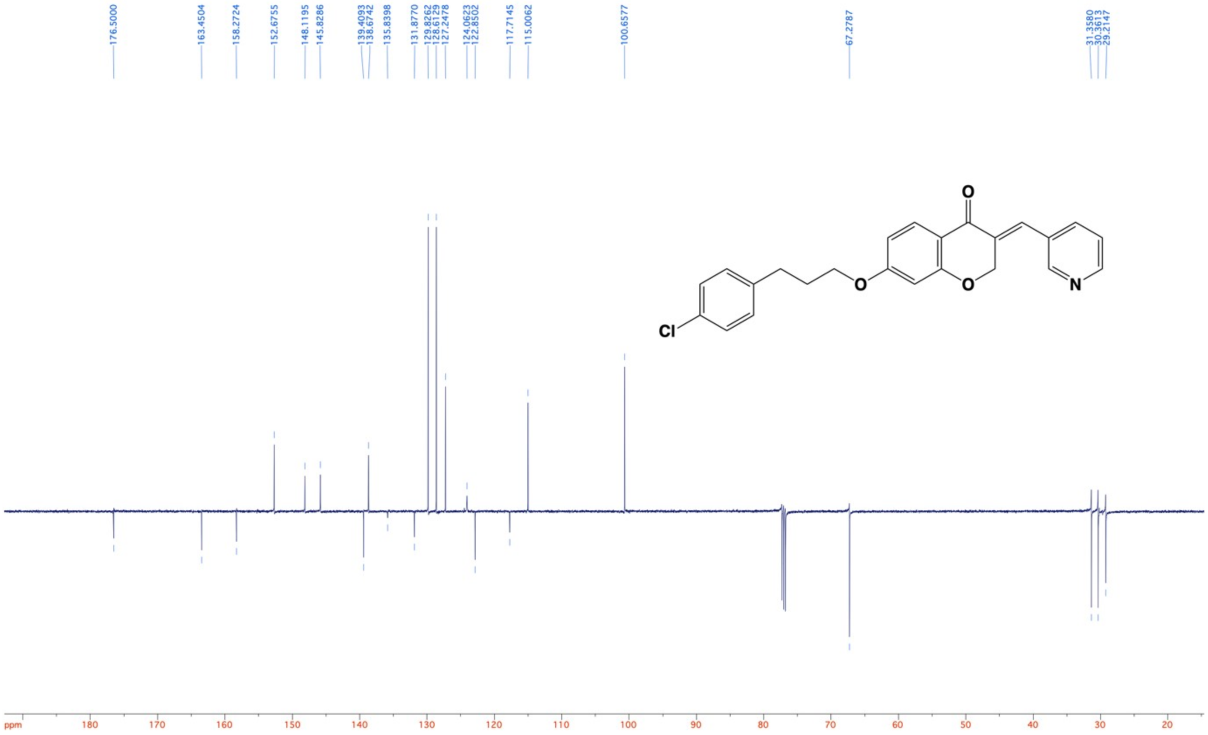

(*E*)-7-(3-(4-bromophenyl)propoxy)-3-(pyridin-3-ylmethylene)chroman-4-one (**5n**)

^{1}H NMR ($CDCl_3$)

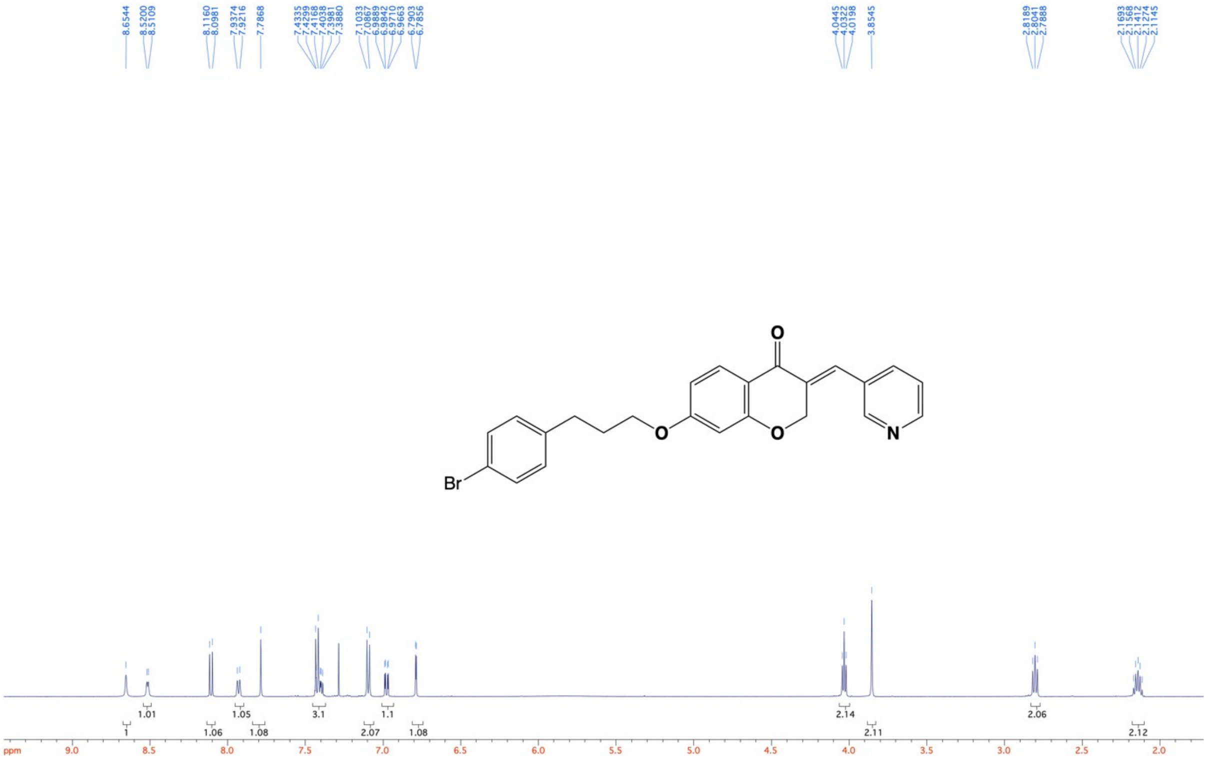

^{13}C NMR ($CDCl_3$)

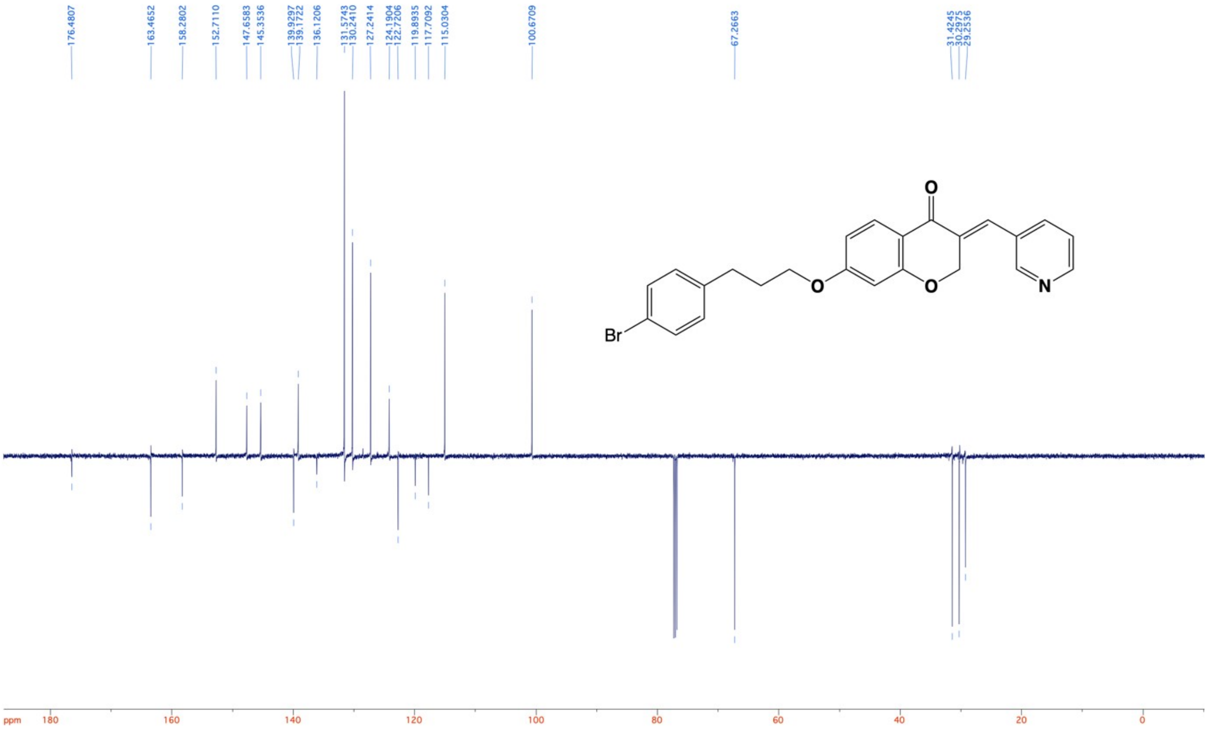

(*E*)-7-(3-(4-Methoxyphenyl)propoxy)-3-(pyridin-3-ylmethylene)chroman-4-one (**5o**)

^{1}H NMR ($CDCl_3$)

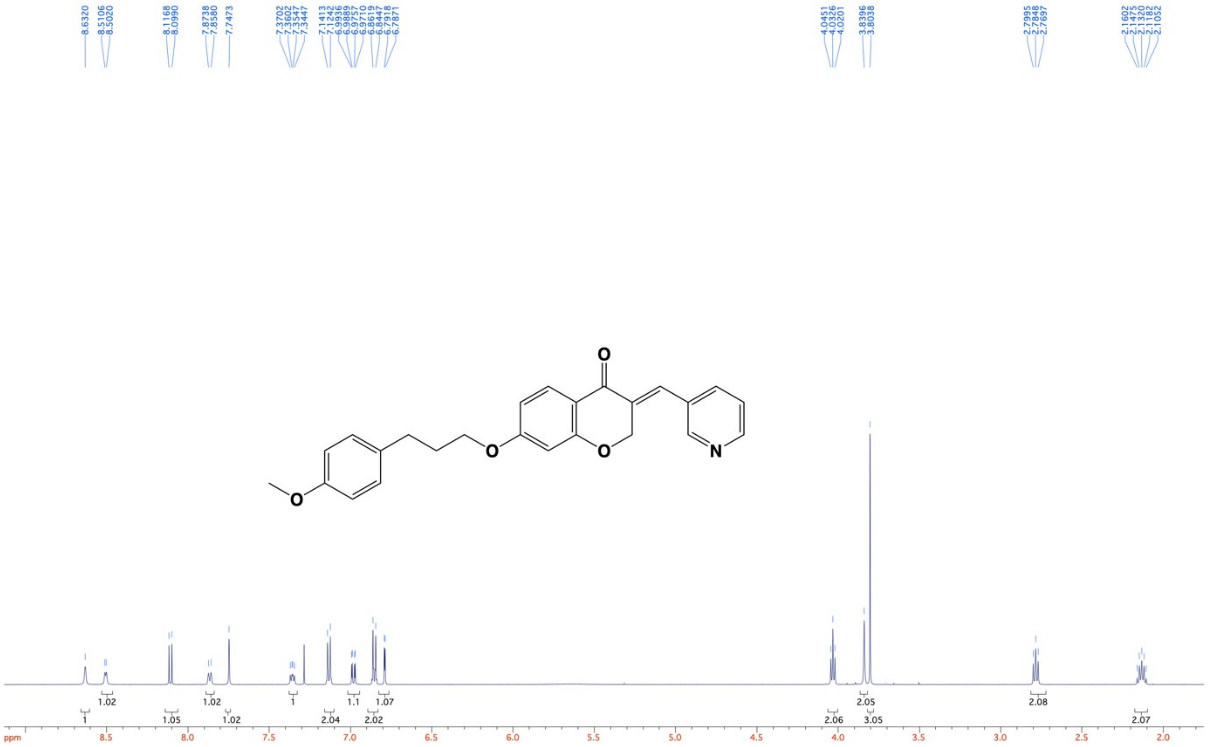

^{13}C NMR ($CDCl_3$)

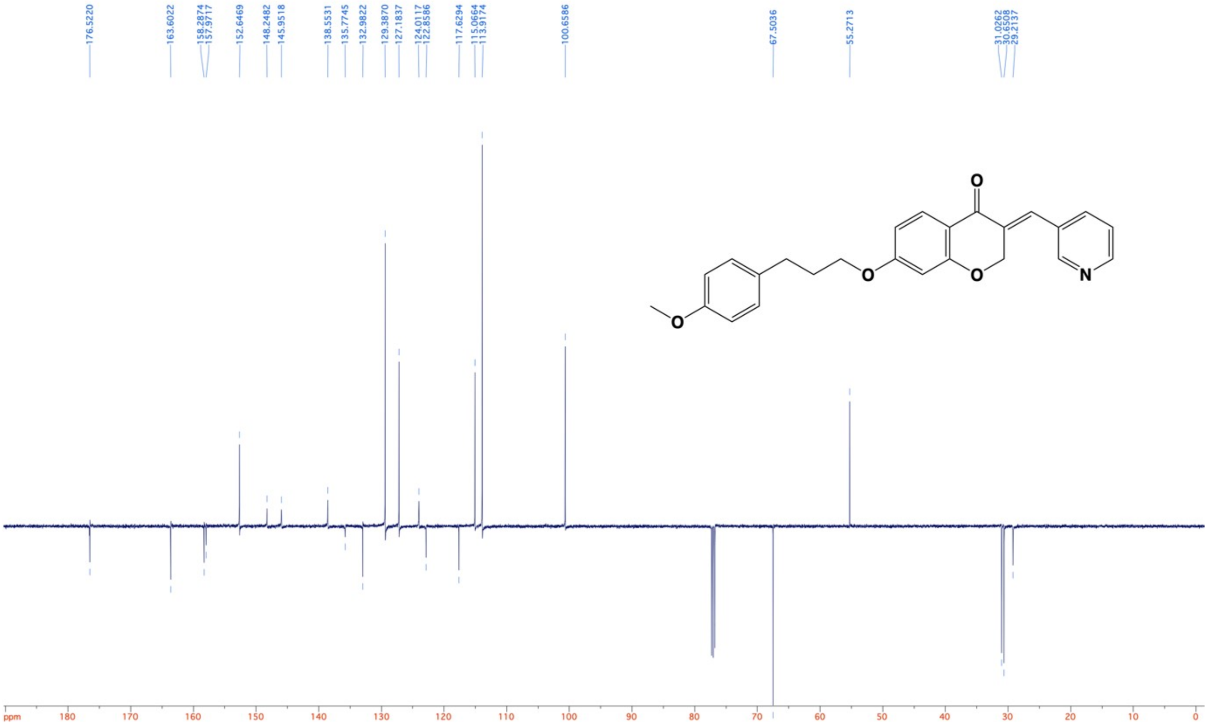

(*E*)-6-Phenethoxy-2-(pyridin-3-ylmethylene)-3,4-dihydronaphthalen-1(2*H*)-one (**8a**):

^{1}H NMR ($CDCl_3$)

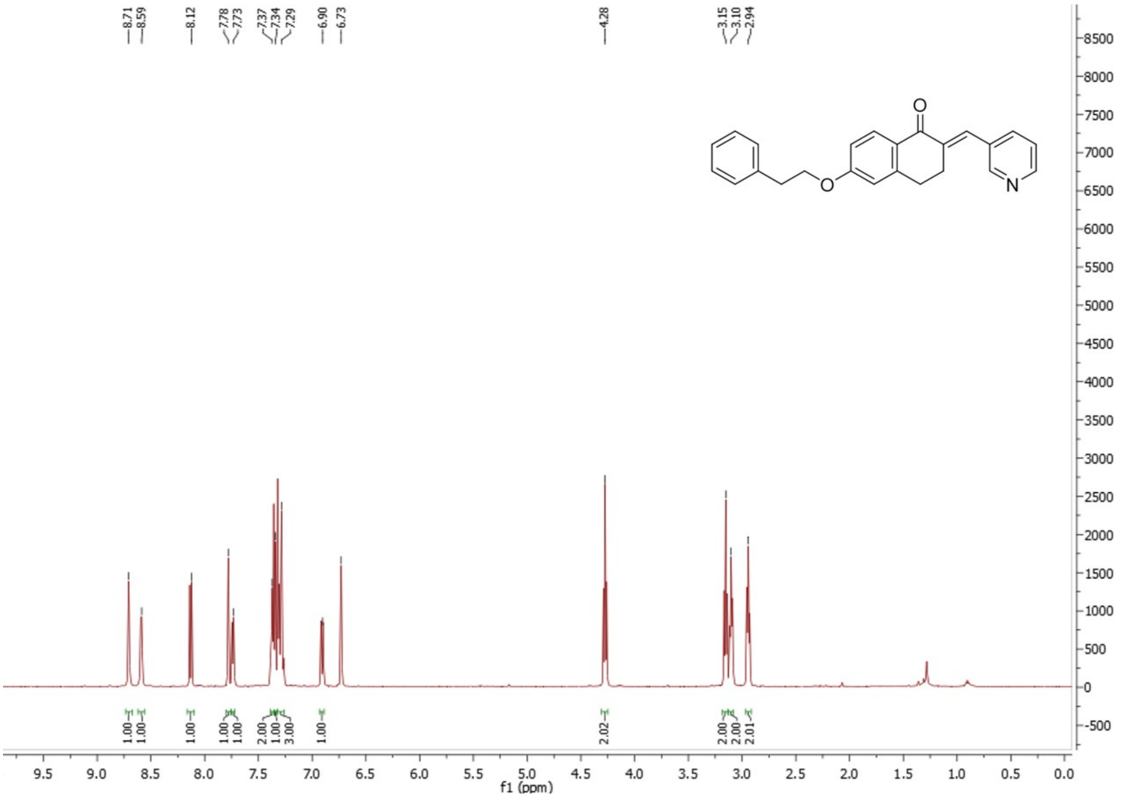

^{13}C NMR ($CDCl_3$)

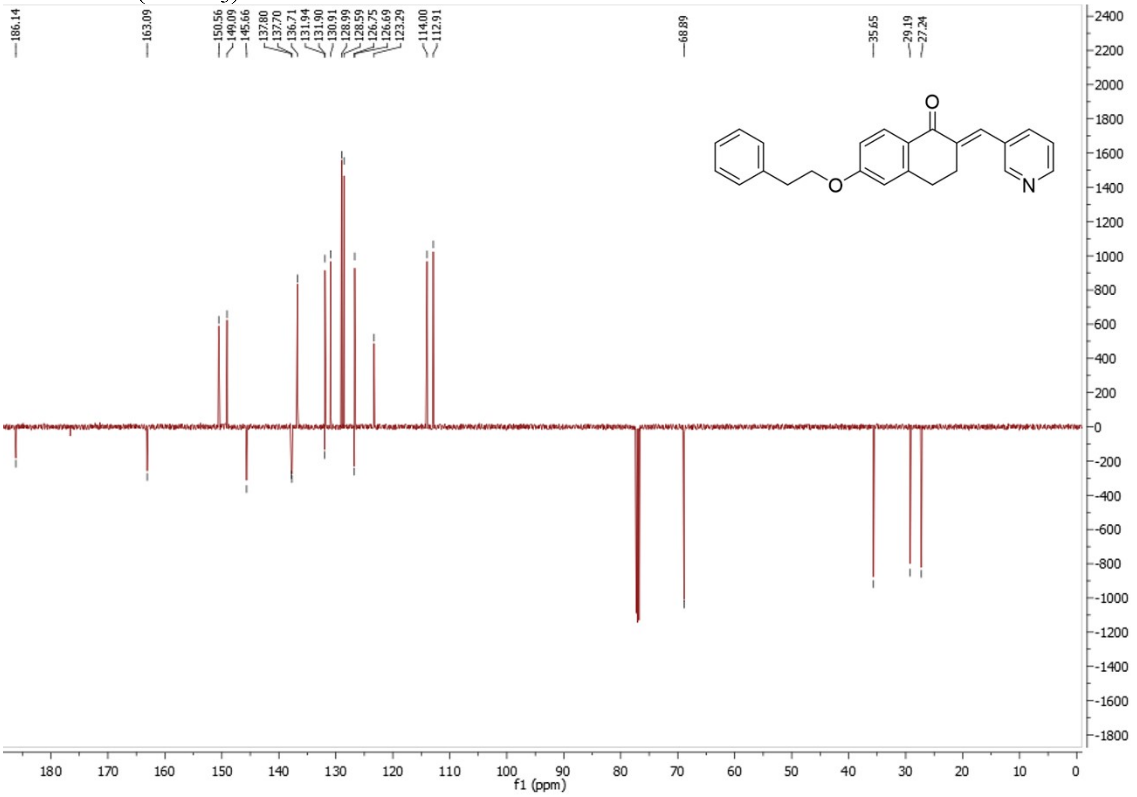

(*E*)-6-(4-Fluorophenethoxy)-2-(pyridin-3-ylmethylene)-3,4-dihydronaphthalen-1(2*H*)-one (**8b**):

^{1}H NMR ($CDCl_3$)

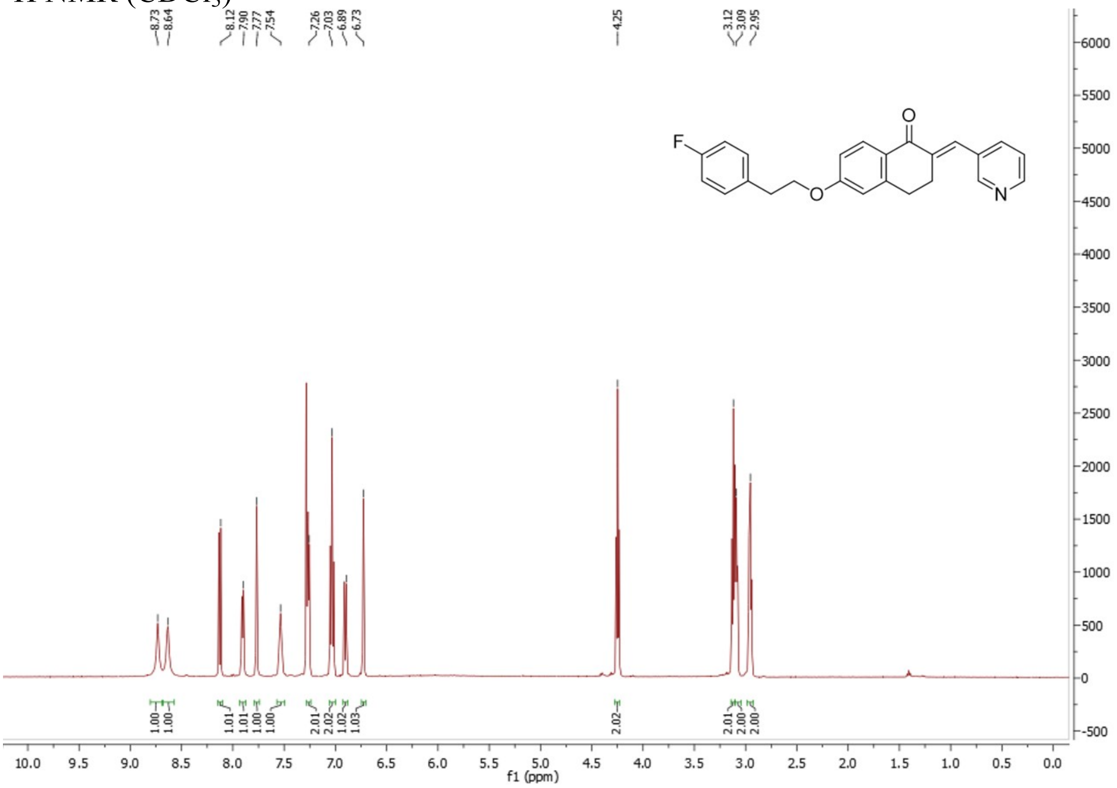

^{13}C NMR ($CDCl_3$)

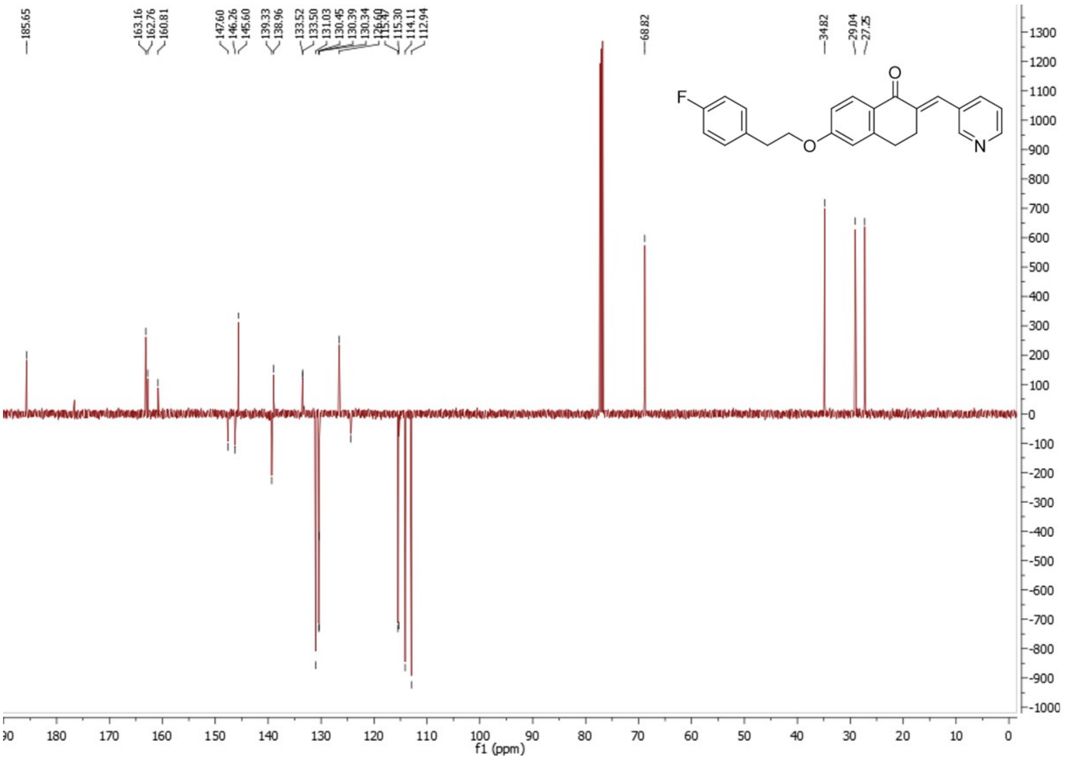

^{19}F NMR ($CDCl_3$)

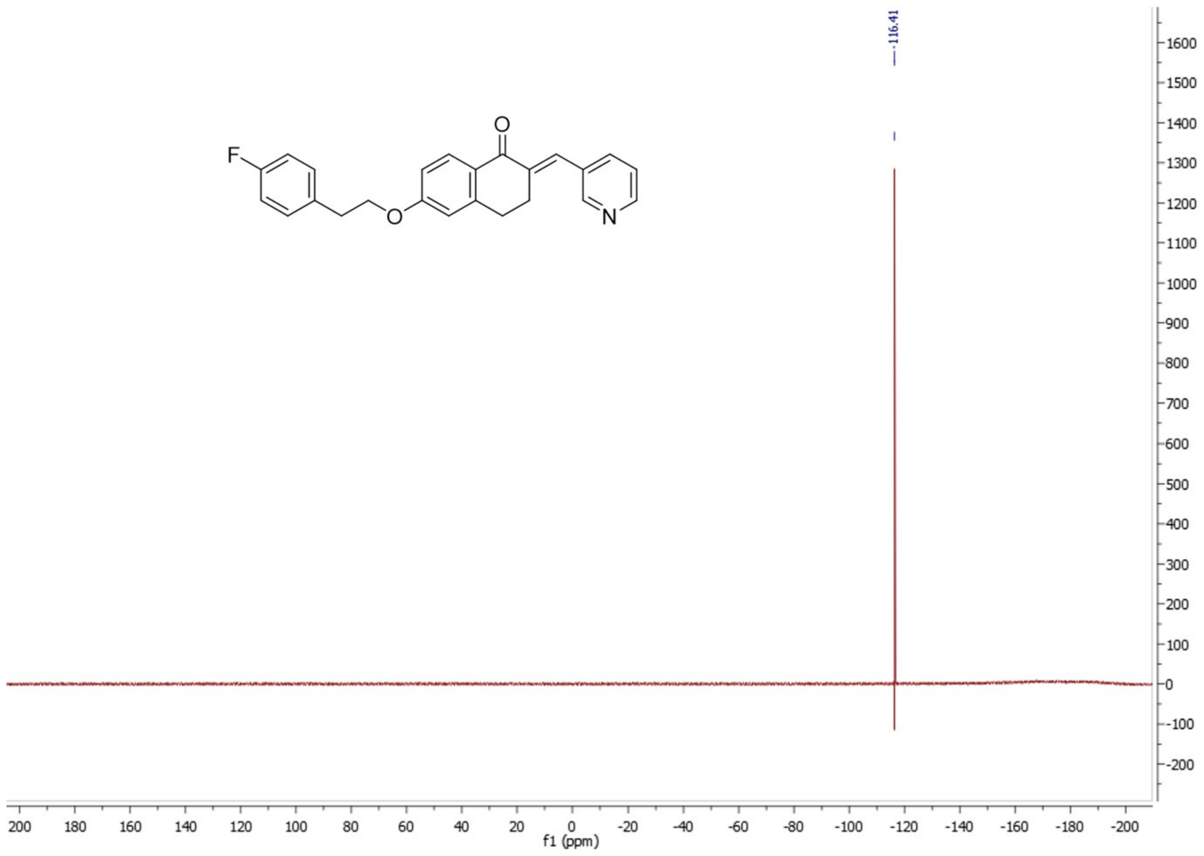

(*E*)-6-(4-Chlorophenethoxy)-2-(pyridin-3-ylmethylene)-3,4-dihydronaphthalen-1(2*H*)-one (**8c**):

^{1}H NMR ($CDCl_3$)

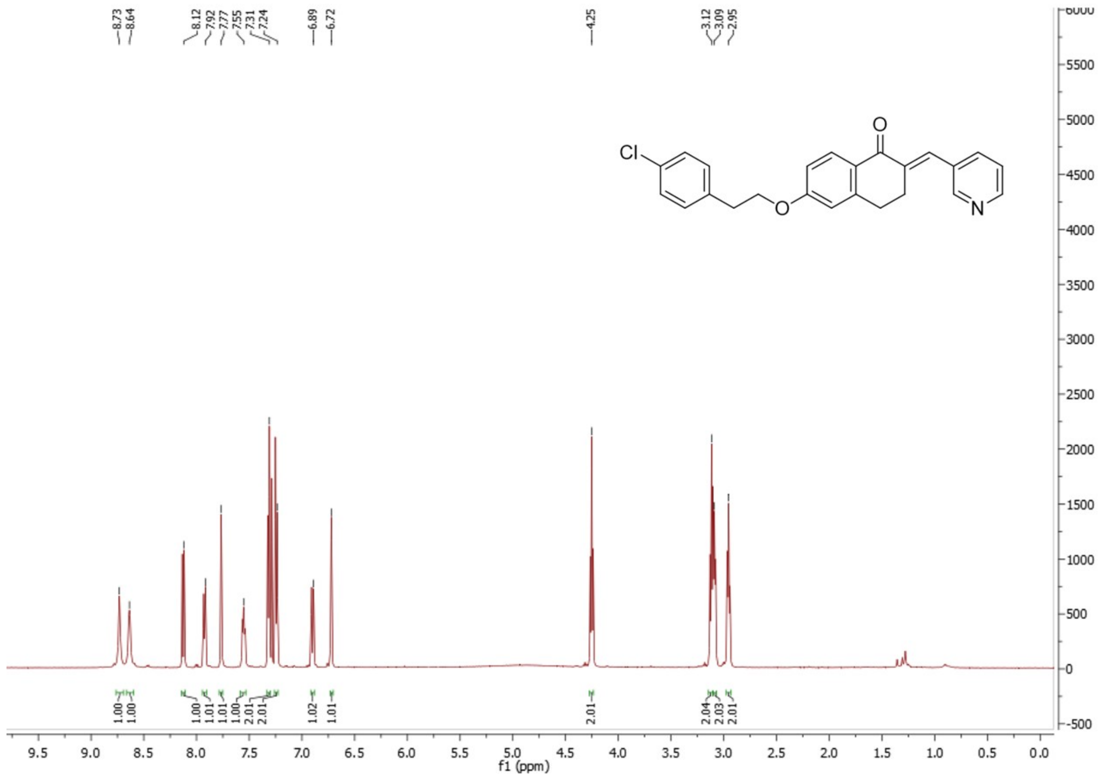

^{13}C NMR ($CDCl_3$)

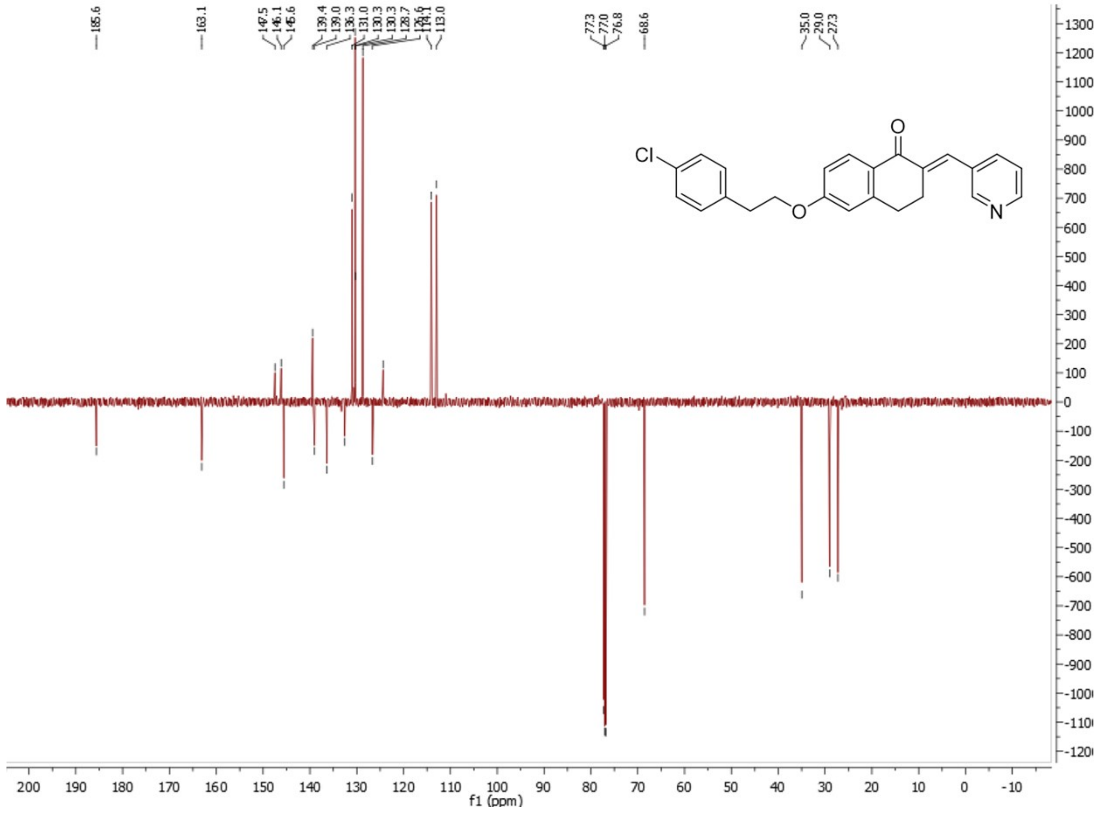

(*E*)-6-(4-Bromophenethoxy)-2-(pyridin-3-ylmethylene)-3,4-dihydronaphthalen-1(2*H*)-one (**8d**):

^{1}H NMR ($CDCl_3$)

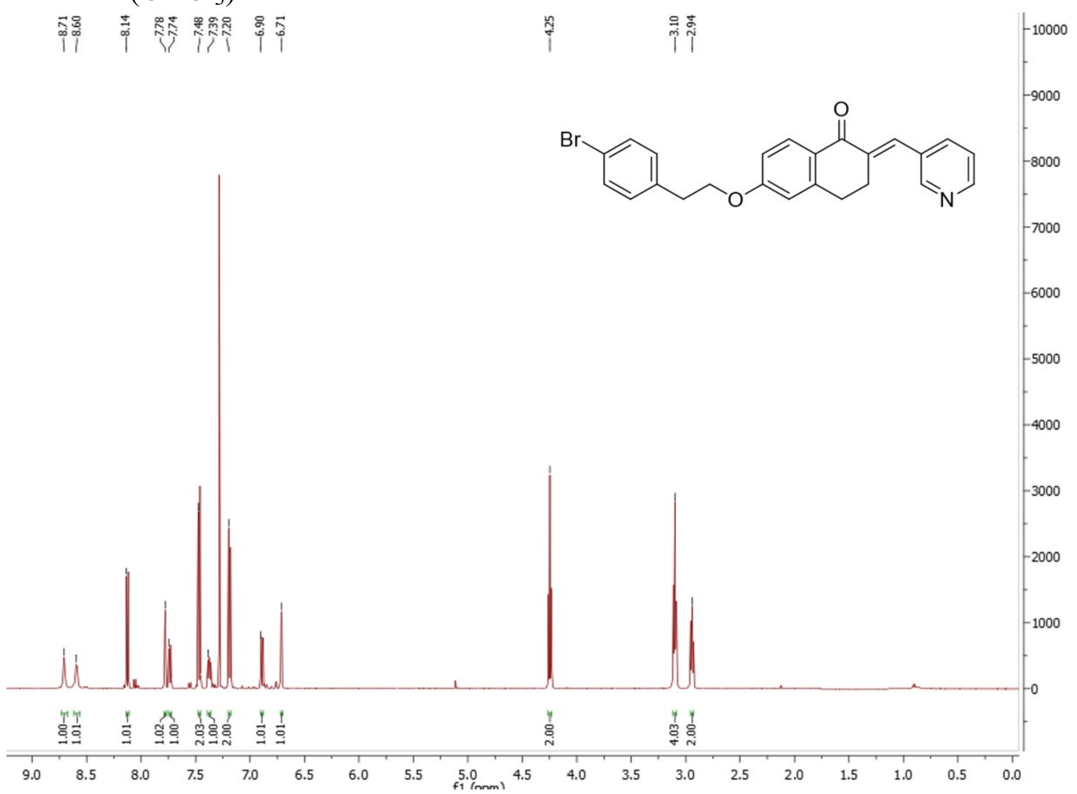

^{13}C NMR ($CDCl_3$)

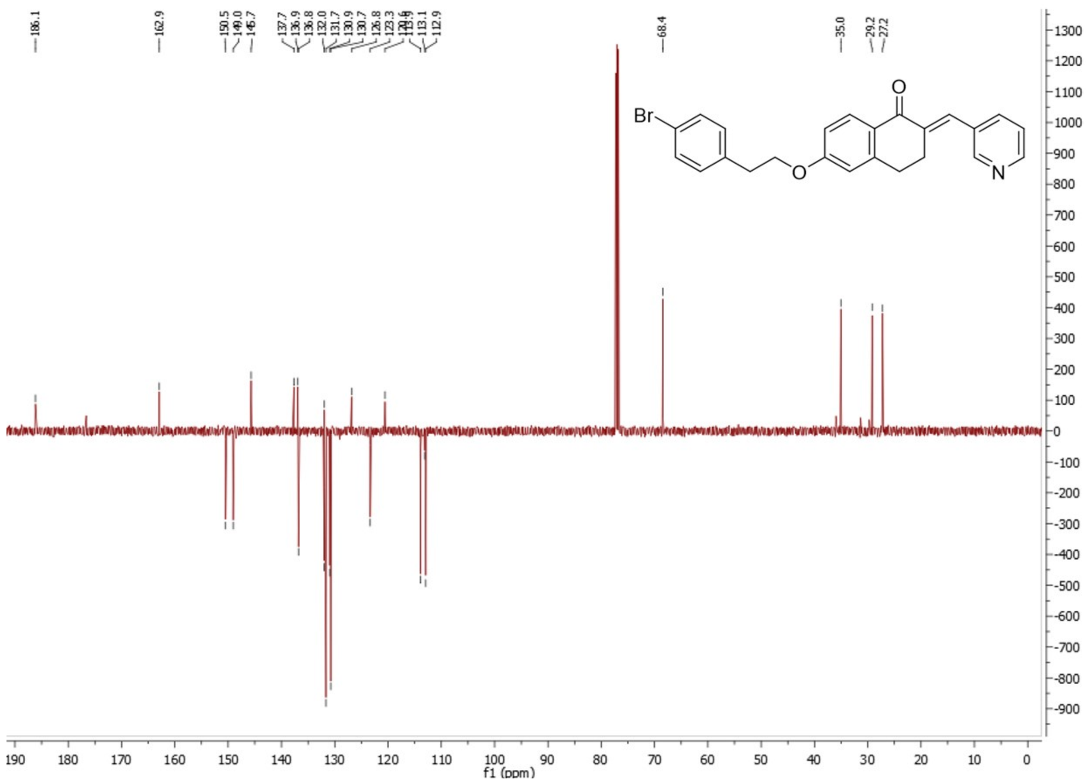

(*E*)-6-(4-Methoxyphenethoxy)-2-(pyridin-3-ylmethylene)-3,4-dihydronaphthalen-1(2*H*)-one (**8e**):

^{1}H NMR ($CDCl_3$)

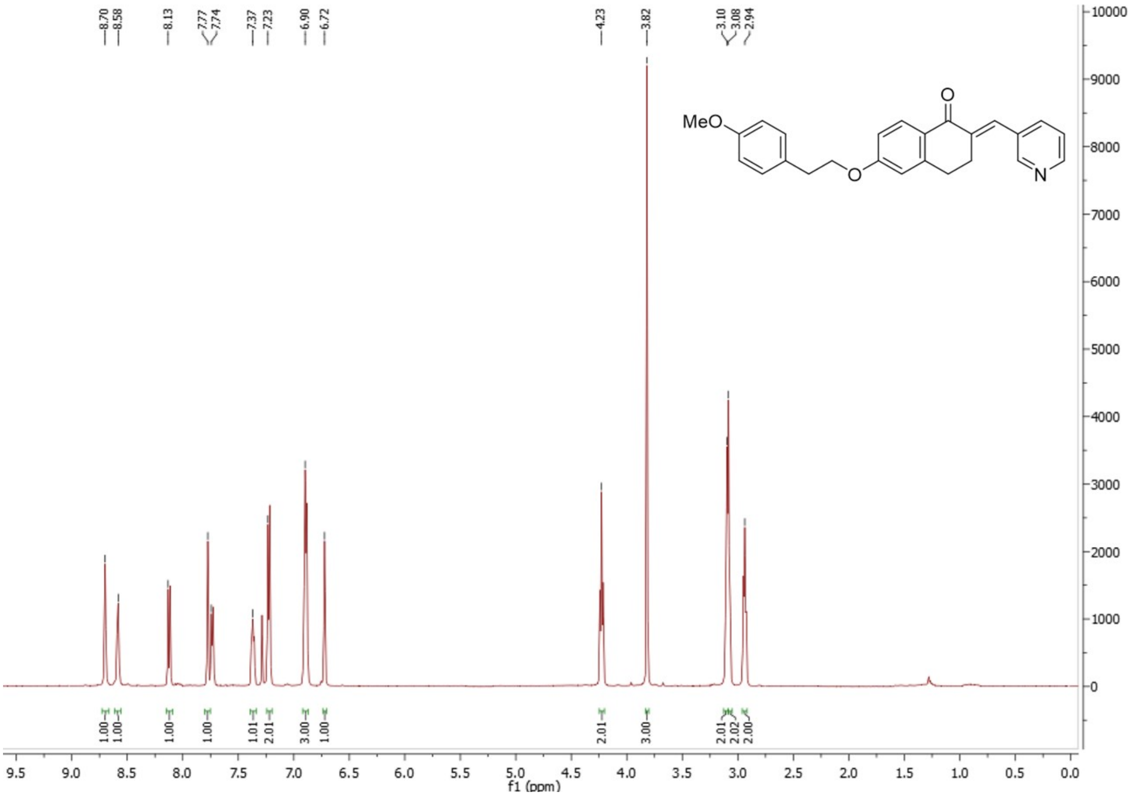

^{13}C NMR ($CDCl_3$)

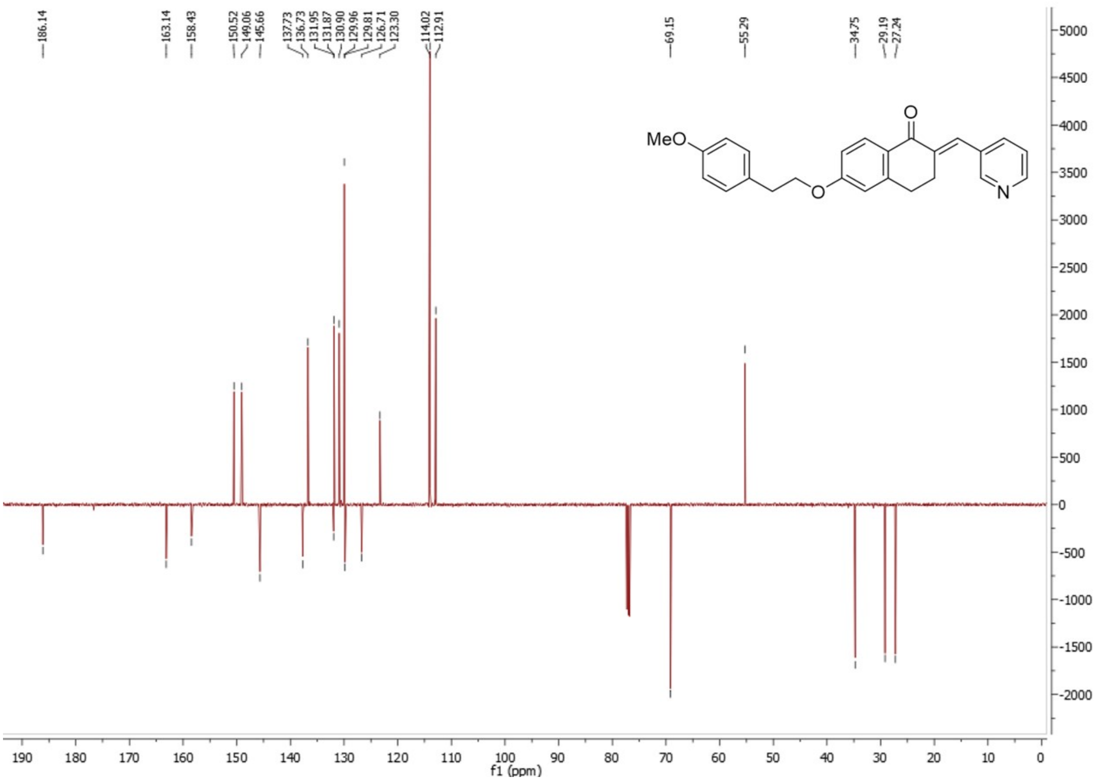

HPLC

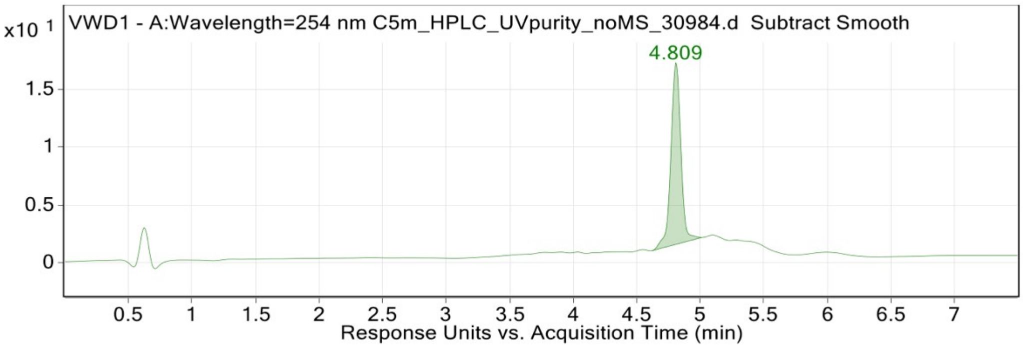

Figure: Base peak or HPLC chromatogram (indicated in left hand corner)

User Chromatogram Peak List

RT (min)	Area	Area %	Area Sum (%)	Width (min)
4.81	85.49	100.00	100.00	0.086

(*E*)-6-(3-Phenylpropoxy)-2-(pyridin-3-ylmethylene)-3,4-dihydronaphthalen-1(2*H*)-one (**8f**):

^{1}H NMR ($CDCl_3$)

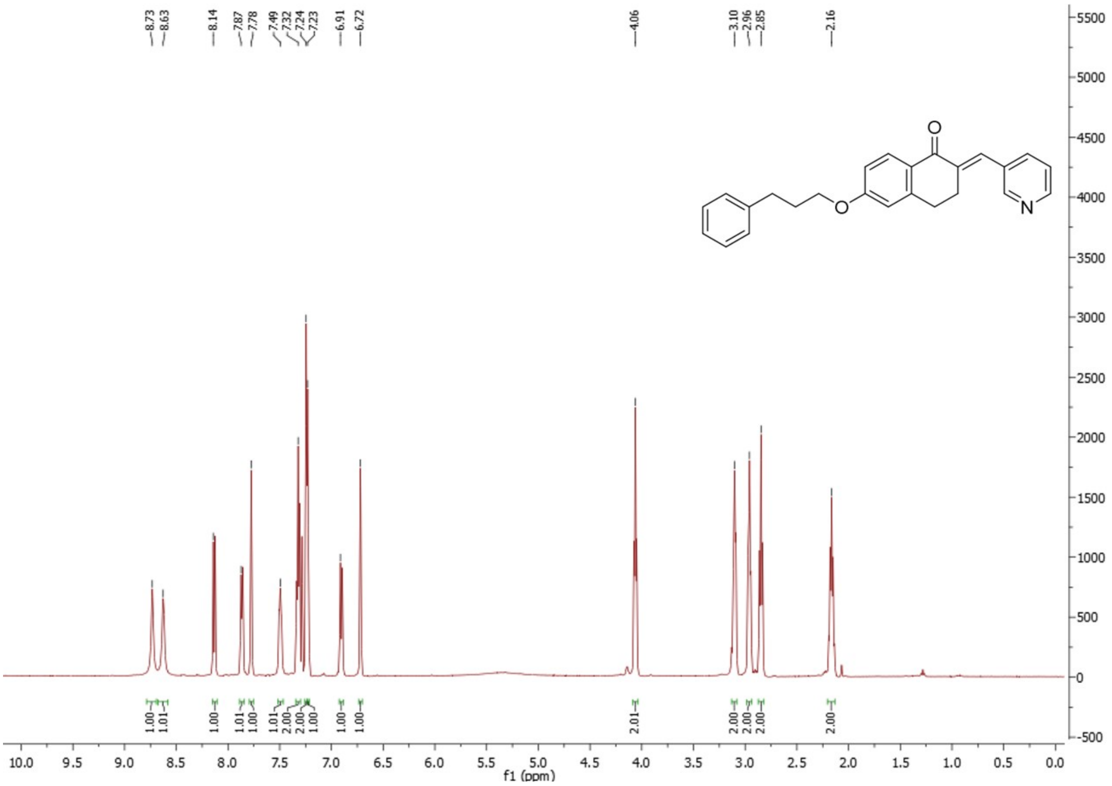

^{13}C NMR ($CDCl_3$)

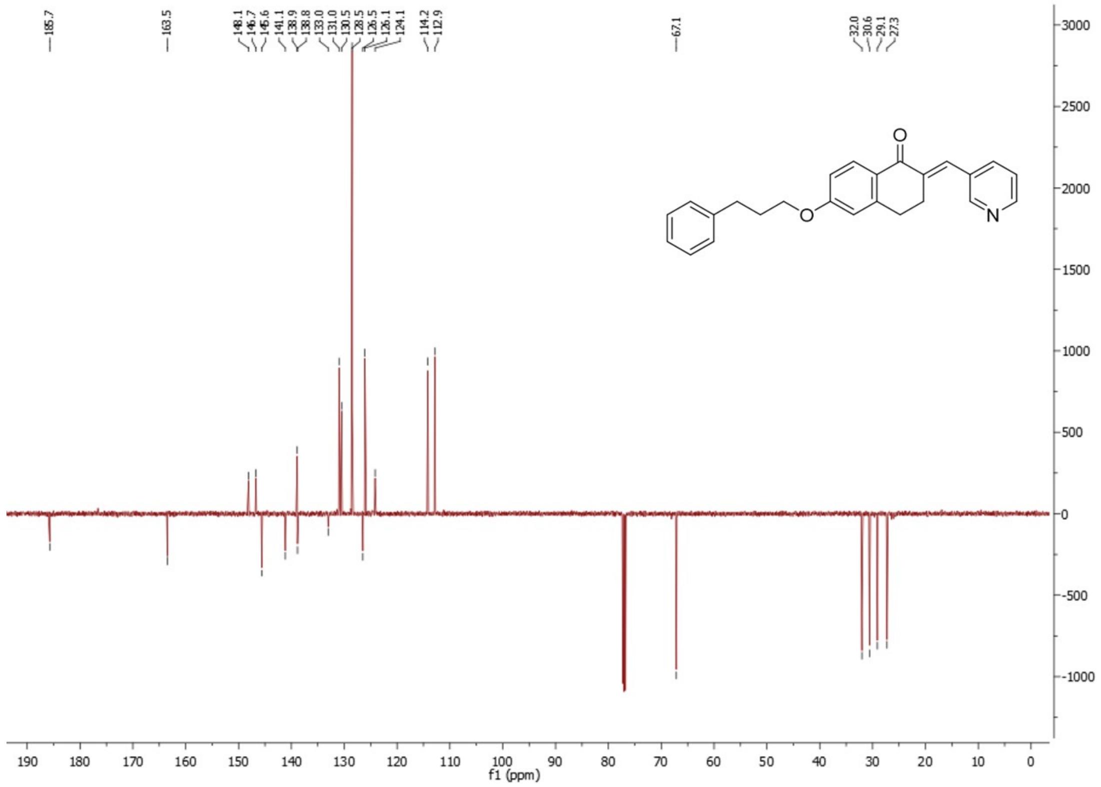

(*E*)-6-(3-(4-Fluorophenyl)propoxy)-2-(pyridin-3-ylmethylene)-3,4-dihydronaphthalen-1(2*H*)-one (**8g**):

^{1}H NMR ($CDCl_3$)

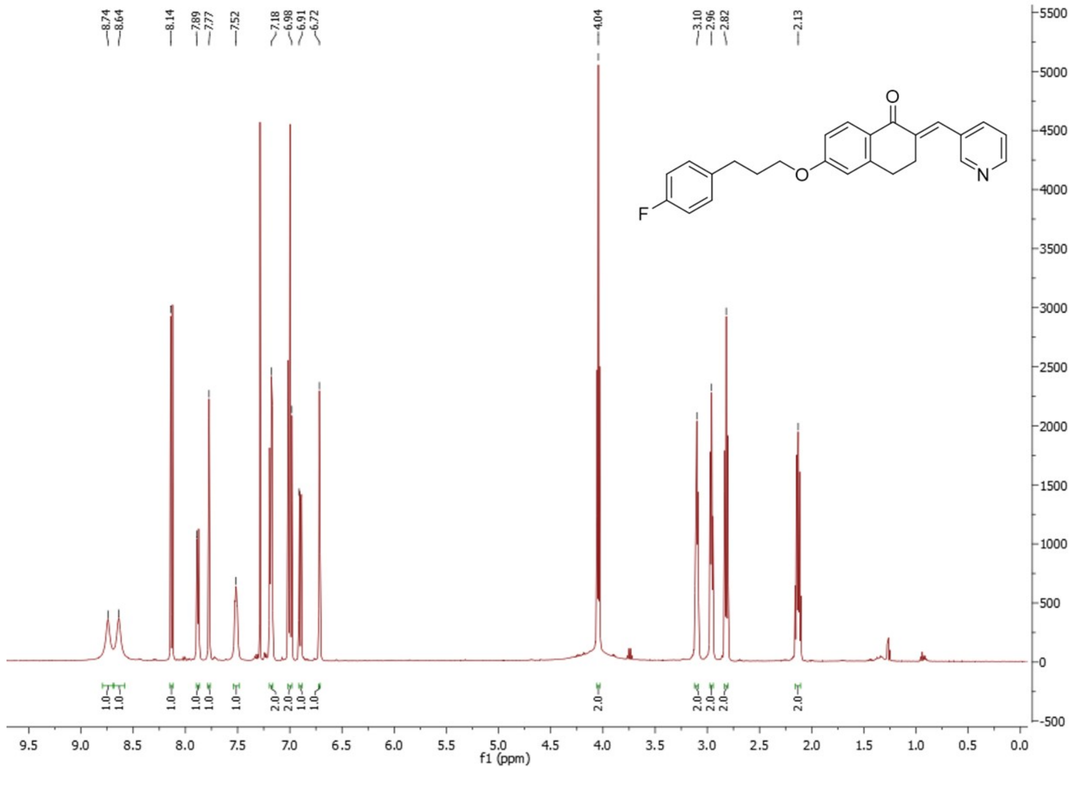

^{13}C NMR ($CDCl_3$)

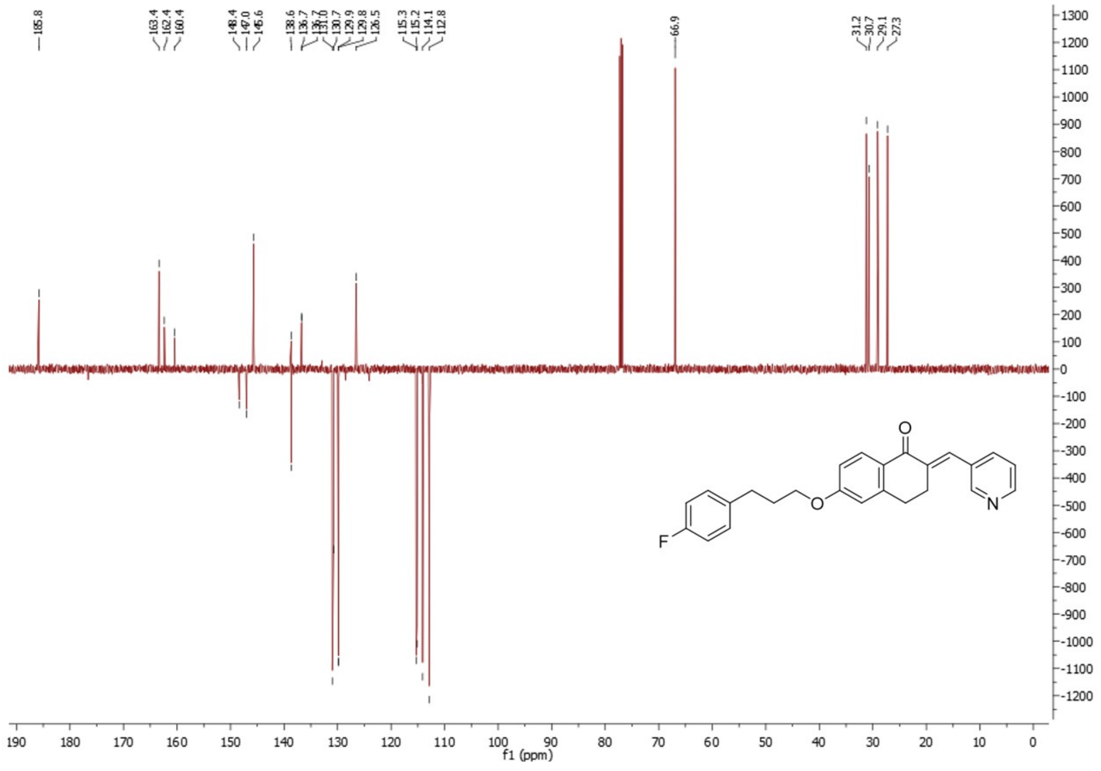

^{19}F NMR ($CDCl_3$)

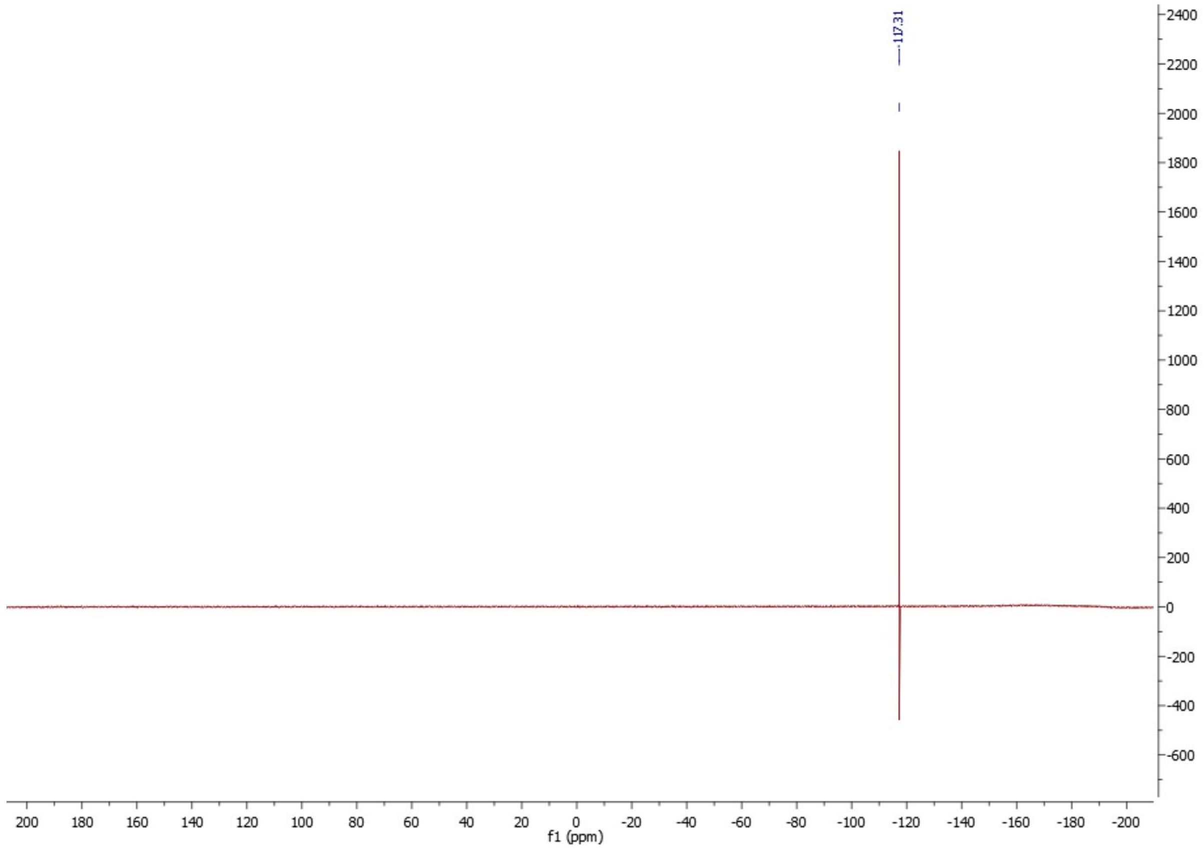

(*E*)-6-(3-(4-Chlorophenyl)propoxy)-2-(pyridin-3-ylmethylene)-3,4-dihydronaphthalen-1(2*H*)-one (**8h**):

^{1}H NMR ($CDCl_3$)

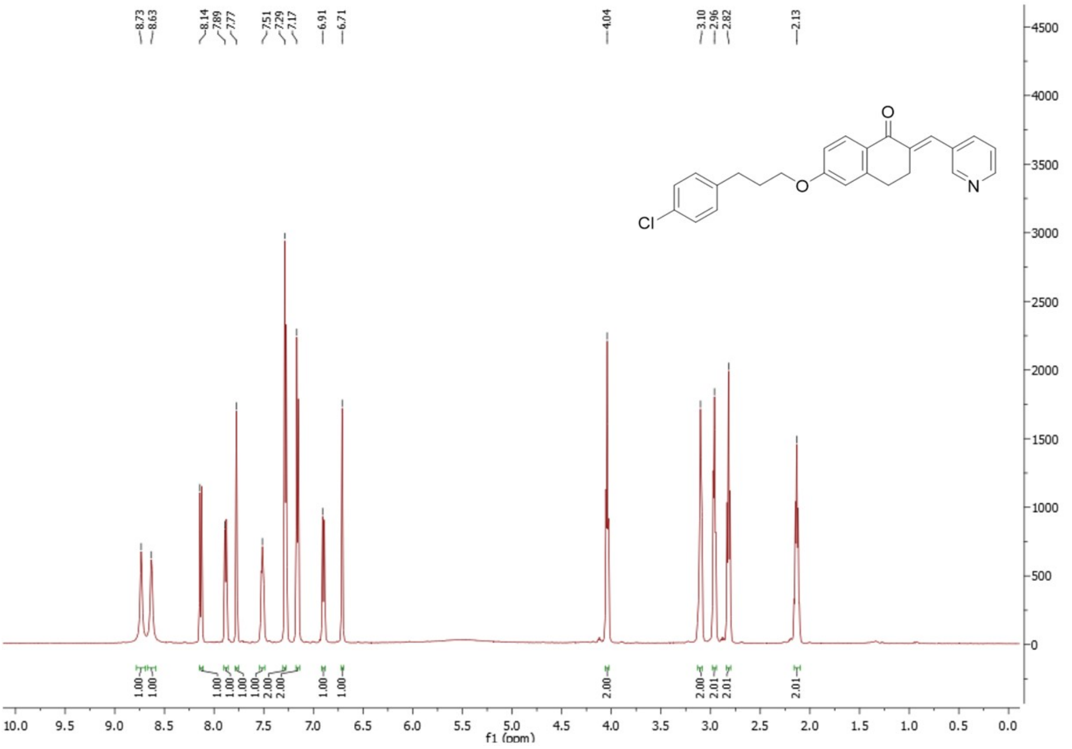

^{13}C NMR ($CDCl_3$)

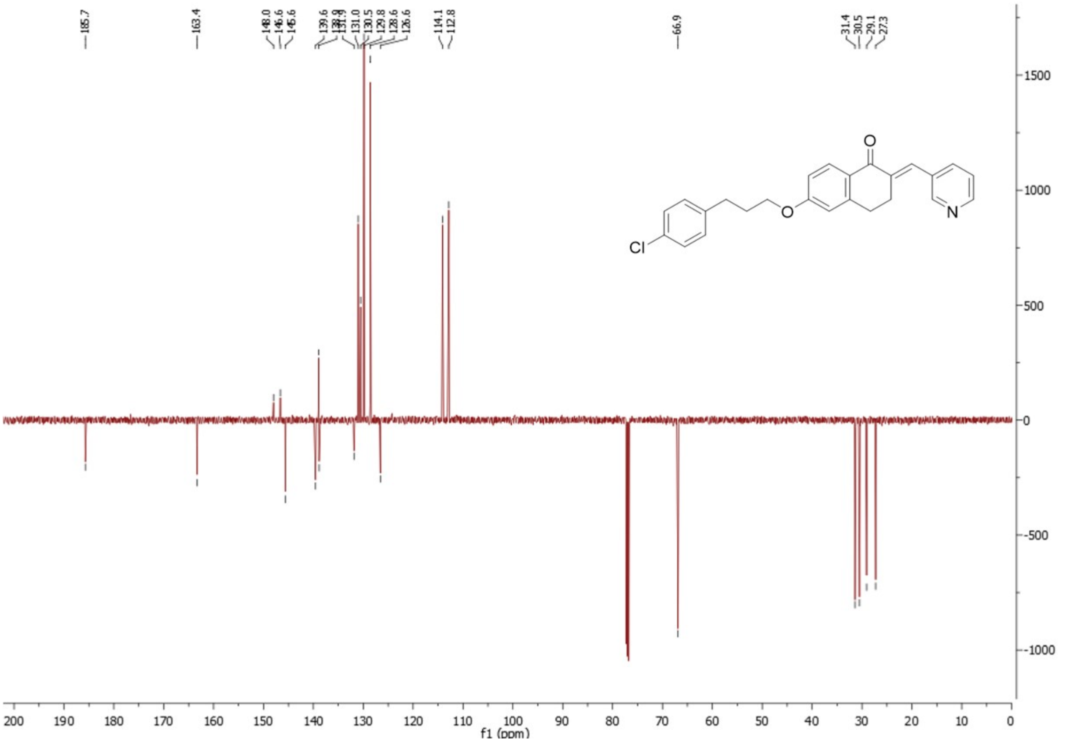

(*E*)-6-(3-(4-Bromophenyl)propoxy)-2-(pyridin-3-ylmethylene)-3,4-dihydronaphthalen-1(2*H*)-one (**8i**):

^{1}H NMR ($CDCl_3$)

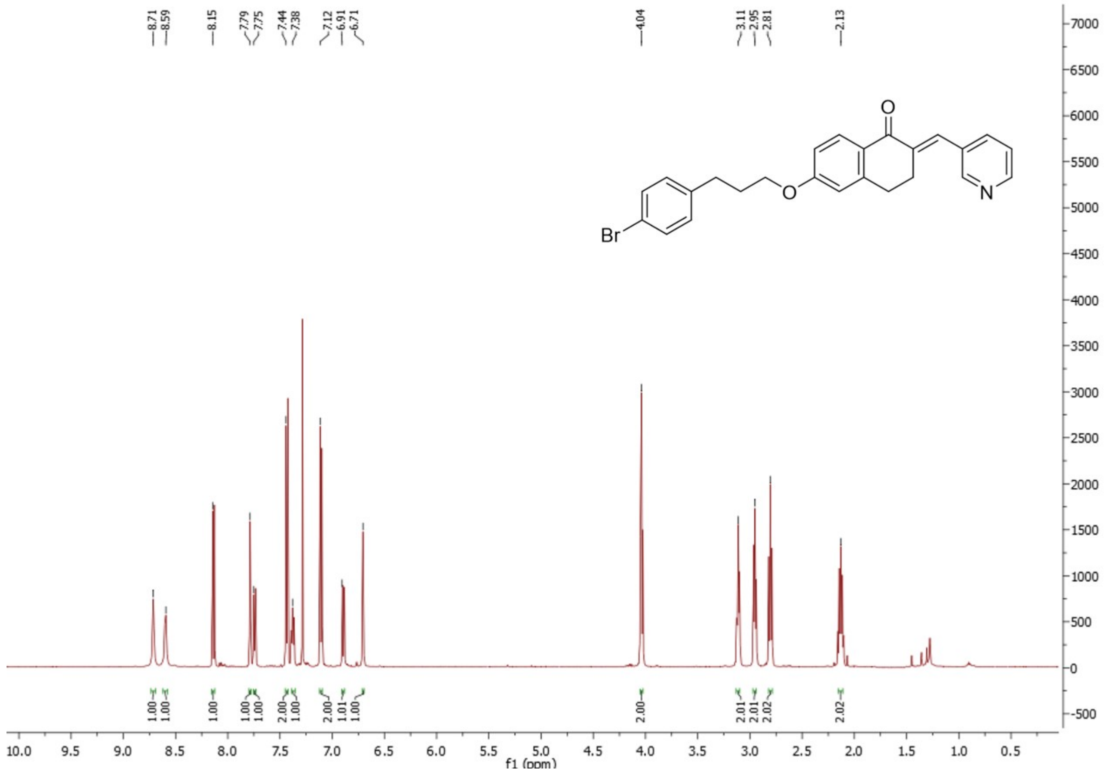

^{13}C NMR ($CDCl_3$)

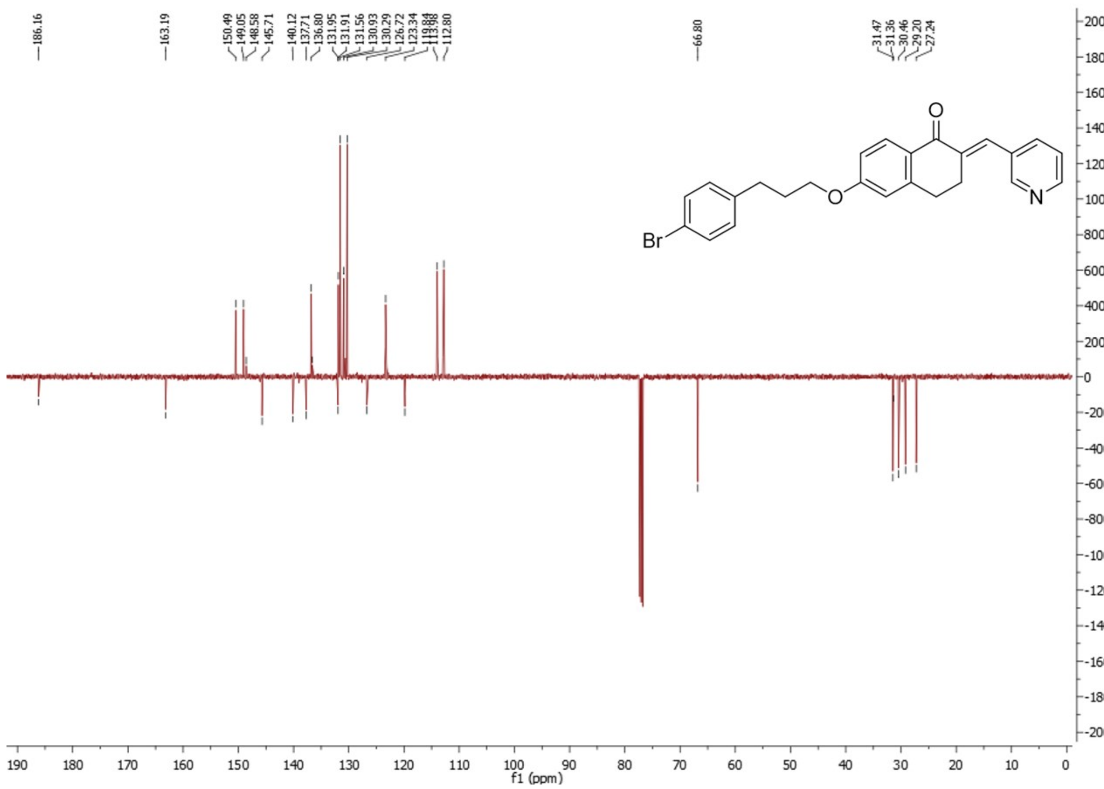

(*E*)-6-(3-(4-Methoxyphenyl)propoxy)-2-(pyridin-3-ylmethylene)-3,4-dihydronaphthalen-1(2*H*)-one (**8j**):

^{1}H NMR ($CDCl_3$)

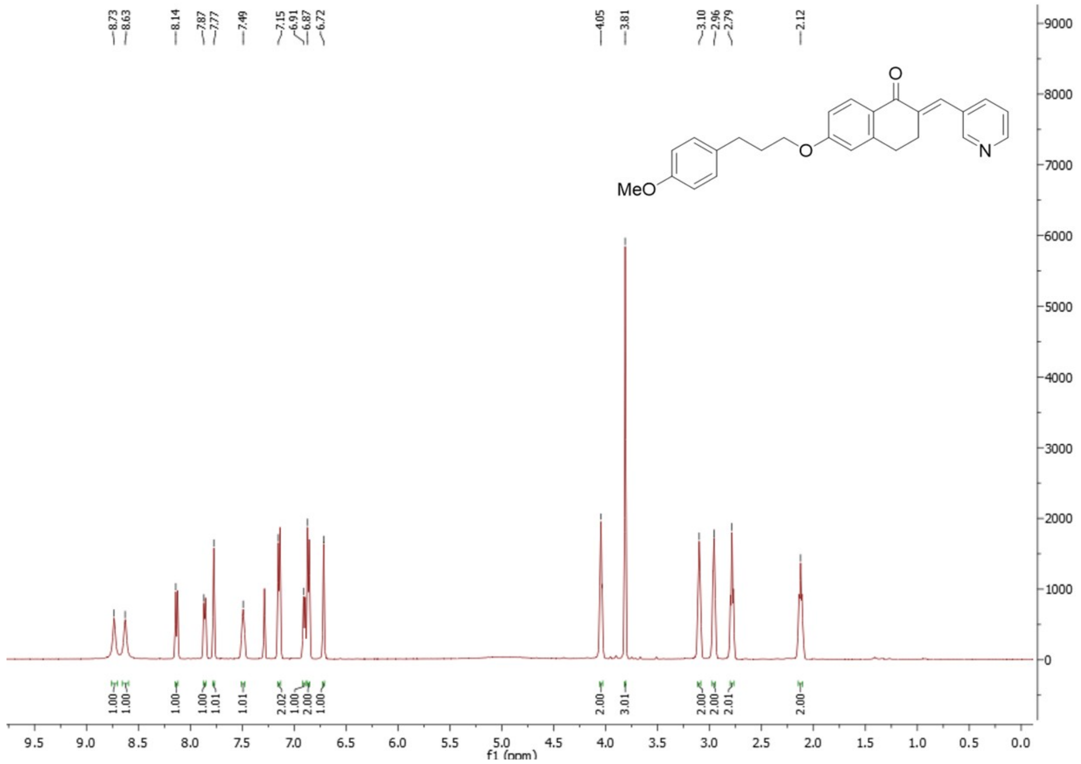

^{13}C NMR ($CDCl_3$)

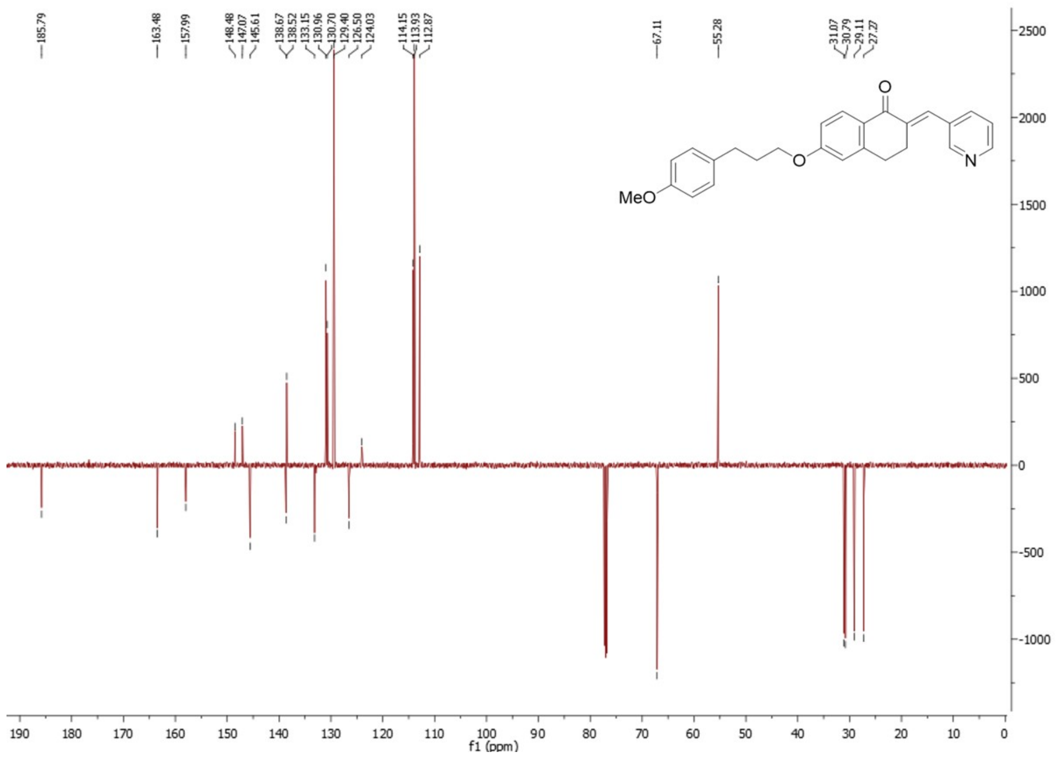

References

(1) Vilchèze, C.; Copeland, J.; Keiser, T. L.; Weisbrod, T.; Washington, J.; Jain, P.; Malek, A.; Weinrick, B.; Jacobs, W. R. Rational design of biosafety level 2-approved, multidrug resistant strains of *Mycobacterium tuberculosis* through nutrient auxotrophy. *mBio.* **2018**, *9* (3), 1–12.

(2) Roy, S. K.; Kumari, N.; Gupta, S.; Pahwa, S.; Nandanwar, H.; Jachak, S. M. 7-Hydroxy (*E*)-3-phenylmethylene-chroman-4-one analogues as efflux pump inhibitors against *Mycobacterium smegmatis* mc^2 155. *Eur. J. Med. Chem.* **2013**, *66*, 499–507.

(3) Zhan, X. Y.; Zhang, H.; Dong, Y.; Yang, J.; He, S.; Shi, Z. C.; Tang, L.; Wang, J.-Y. Chemoselective hydrosilylation of the α,β-site double bond in α,β-and α,β,γ,δ-unsaturated ketones catalyzed by macrosteric borane promoted by hexafluoro-2-propanol. *J. Org. Chem.* **2020**, *85* (10), 6578-6592.

(4) Fitton, A. O.; Ramage, G. R. Chromanones derived from resorcinol. *J. Chem. Soc.* **1962**, 4870–4874.

(5) Wang, Z.; Wu, J.; Yang, X.; Cai, P.; Liu, Q.; Wang, K. D. G.; Kong, L.; Wang, X. Neuroprotective effects of benzyloxy substituted small molecule monoamine oxidase B inhibitors in Parkinson's disease. *Bioorganic Med. Chem.* **2016**, *24* (22), 5929–5940.

(6) Lan J-S, Xie S-S, Huang M, Hu Y-J, Kong L-Y, Wang X-B. Chromanones: selective and reversible monoamine oxidase B inhibitors with nanomolar potency. *MedChemComm*. 2015;

(7) Legoabe, L. J.; Petzer, A.; Petzer, J. P. α-Tetralone derivatives as inhibitors of monoamine oxidase. *Bioorganic Med. Chem. Lett.* **2014**, *24* (12), 2758–2763.
(8) Kuang Yuan, L.; Meng Hsien, L.; Yan-Feng, J.; Yu-Shiou, F.; Chi-Han, C.; Sheng Hung, L.
Benzene fused heterocyclic derivative and pharmaceutical. *World Intellectual Property Organisation.* WO 2019/051222 A1, **2019**.
(9) Guzman, J. D.; Evangelopoulos, D.; Gupta, A.; Birchall, K.; Mwaigwisya, S.; Saxty, B.; McHugh, T. D.; Gibbons, S.; Malkinson, J.; Bhakta, S. Antitubercular specific activity of ibuprofen and the other 2-arylpropanoic acids using the HT-SPOTi whole-cell phenotypic assay. *BMJ Open* **2013**, *3* (6), e002672.
(10) Palomino J.C., Martin A., Camacho M., Guerra H., Swings J., Portaels F. Resazurin microtiter assay plate: Simple and inexpensive method for detection of drug resistance in Mycobacterium tuberculosis. *Antimicrob. Agents Chemother.* **2002**, *46*, 2720–2722.
(11) Kumar, A.; Campomizzi, C. S.; Jay, N.; Ferguson, S.; Scheffler, E. J.; Lioi, J.; Tu, C.; Qu, J.; Simons, C.; Estrada, D. F. Surface hydrophobics mediate functional dimerization of CYP121A1 of *Mycobacterium tuberculosis*. *Sci. Rep.* **2021**, *11* (1), 394.
(12) Molecular Operating Enviroment (MOE). Chemical Computing Group ULC, **2024**, https://www.chemcomp.com/ Products.htm.
(13) Abd El-wahab, H.A.A.; Accietto, M.; Marino, L.B.; McLean, K.J.; Levy, C.W.; Abdel-Rahman, H.M.; El-Gendy, M.A.; Munro, A.W.; Aboraia, A.S.; Simons, C. Design, synthesis and evaluation against *Mycobacterium tuberculosis* of azole piperazine derivatives as dicyclotyrosine (cYY) mimics. *Bioorganic & Medicinal Chemistry* **2018**, *26* (1), 161-176. DOI: 10.1016/j.bmc.2017.11.030.
(14) Desmond Schrödinger Release 2020-1, Desmond Molecular Dynamics System, D.E. Shaw Research, New York, NY, Maestro Desmond interoperability tools, Schrödinger, NewYork, NY, **2020**, https://www.schrodinger.com/products/ desmond
